# Tumor immune microenvironment characterization in clear cell renal cell carcinoma identifies prognostic and immunotherapeutically relevant messenger RNA signatures

**DOI:** 10.1186/s13059-016-1092-z

**Published:** 2016-11-17

**Authors:** Yasin Şenbabaoğlu, Ron S. Gejman, Andrew G. Winer, Ming Liu, Eliezer M. Van Allen, Guillermo de Velasco, Diana Miao, Irina Ostrovnaya, Esther Drill, Augustin Luna, Nils Weinhold, William Lee, Brandon J. Manley, Danny N. Khalil, Samuel D. Kaffenberger, Yingbei Chen, Ludmila Danilova, Martin H. Voss, Jonathan A. Coleman, Paul Russo, Victor E. Reuter, Timothy A. Chan, Emily H. Cheng, David A. Scheinberg, Ming O. Li, Toni K. Choueiri, James J. Hsieh, Chris Sander, A. Ari Hakimi

**Affiliations:** 1 Computational Biology Center, Memorial Sloan Kettering Cancer Center, New York, NY USA; 2 Molecular Pharmacology and Chemistry Program, Memorial Sloan Kettering Cancer Center, New York, NY USA; 3 Urology Service, Department of Surgery, Memorial Sloan Kettering Cancer Center, New York, NY USA; 4 Immunology Program, Memorial Sloan Kettering Cancer Center, New York, NY USA; 5 Department of Medical Oncology, Dana-Farber Cancer Institute, Boston, MA USA; 6 Department of Epidemiology and Biostatistics, Memorial Sloan Kettering Cancer Center, New York, NY USA; 7 Department of Radiation Oncology, Memorial Sloan Kettering Cancer Center, New York, NY USA; 8 Department of Pathology, Memorial Sloan Kettering Cancer Center, New York, NY USA; 9 Sidney Kimmel Comprehensive Cancer Center, Johns Hopkins University School of Medicine, Baltimore, MD USA; 10 Vavilov Institute of General Genetics, Russian Academy of Sciences, Moscow, Russia; 11 Genitourinary Oncology Service, Department of Medicine, Memorial Sloan Kettering Cancer Center, New York, NY USA; 12 Human Oncology & Pathogenesis Program, Memorial Sloan Kettering Cancer Center, New York, NY USA; 13 Department of Medicine, Memorial Sloan Kettering Cancer Center, New York, NY USA; 14 Weill Cornell Medical College, New York, NY USA; 15 Present address: Swim Across America/Ludwig Collaborative Laboratory, Department of Medicine, Memorial Sloan Kettering Cancer Center, New York, NY USA

**Keywords:** Tumor immune microenvironment, Checkpoint blockade, Clear cell renal cell carcinoma (ccRCC), Computational deconvolution, Cancer immunotherapy

## Abstract

**Background:**

Tumor-infiltrating immune cells have been linked to prognosis and response to immunotherapy; however, the levels of distinct immune cell subsets and the signals that draw them into a tumor, such as the expression of antigen presenting machinery genes, remain poorly characterized. Here, we employ a gene expression-based computational method to profile the infiltration levels of 24 immune cell populations in 19 cancer types.

**Results:**

We compare cancer types using an immune infiltration score and a T cell infiltration score and find that clear cell renal cell carcinoma (ccRCC) is among the highest for both scores. Using immune infiltration profiles as well as transcriptomic and proteomic datasets, we characterize three groups of ccRCC tumors: T cell enriched, heterogeneously infiltrated, and non-infiltrated. We observe that the immunogenicity of ccRCC tumors cannot be explained by mutation load or neo-antigen load, but is highly correlated with MHC class I antigen presenting machinery expression (APM). We explore the prognostic value of distinct T cell subsets and show in two cohorts that Th17 cells and CD8^+^ T/Treg ratio are associated with improved survival, whereas Th2 cells and Tregs are associated with negative outcomes. Investigation of the association of immune infiltration patterns with the subclonal architecture of tumors shows that both APM and T cell levels are negatively associated with subclone number.

**Conclusions:**

Our analysis sheds light on the immune infiltration patterns of 19 human cancers and unravels mRNA signatures with prognostic utility and immunotherapeutic biomarker potential in ccRCC.

**Electronic supplementary material:**

The online version of this article (doi:10.1186/s13059-016-1092-z) contains supplementary material, which is available to authorized users.

## Background

Tumors are complex environments, composed of transformed cells as well as stromal and immune infiltrates. Tumor-infiltrating cells can demonstrate either tumor-suppressive or tumor-promoting effects, depending on the cancer type or the tumor model. For instance, regulatory T cells (Tregs) and tumor associated macrophages (TAMs) have been associated with pro-tumor functions [1–3], whereas CD8^+^ T cells have been associated with improved clinical outcomes and response to immunotherapy [4–8]. Antitumor activity of antigen-specific CD8^+^ T cells may underlie the efficacy of immune checkpoint blockade therapy [9–11] as such CD8^+^ T cells have been shown to increase in quantity and activity after treatment with these drugs.

CD8^+^ T cells are activated by peptide antigens presented on major histocompatibility class I (MHC-I) molecules. A CD8^+^ T cell can proliferate when its T cell receptor (TCR) recognizes antigens presented by MHC-I on a target cell, leading to an antigen-specific immune response that kills antigen-bearing cells [12]. All nucleated cells express antigen presenting machinery (APM) genes that code for MHC-I subunits and proteins necessary to process antigens and load them onto MHC-I. The APM genes can be upregulated by type II interferon (IFNγ), which is secreted by activated CD8^+^ T cells and other immune infiltrates. Upregulation of APM genes can lead to a cytotoxic feed-forward loop: more antigen presentation increases the number of T cells that find their cognate antigens, which in turn increases IFNγ release, antigen presentation, and cytotoxicity. Yet identification of CD8^+^ T cells alone is not sufficient to characterize the cytotoxic potential of the complex tumor microenvironment. The net inflammatory nature of the tumor can better be understood by quantifying the infiltration levels of diverse immune cell types.

Tumor immune infiltrates have largely been characterized by tissue-based approaches such as immunohistochemistry (IHC) and flow cytometry. These approaches are limited by a number of factors including the number of cell types that can be assayed simultaneously and the amount of tissue required. Computational techniques applied to gene expression profiles of bulk tumors can rapidly provide a broader perspective on the intratumoral immune landscape [13, 14]. Single sample gene set enrichment analysis (ssGSEA) has previously been successfully implemented to profile the overall immune and stromal infiltration levels across multiple cancer types [15]. Deconvolution methods such as CIBERSORT [16] and DeconRNA-Seq [17] have also recently been developed, but either have not yet been validated for RNA sequencing (RNA-Seq) data or require reference expression vectors for each individual tumor-infiltrating immune cell population that are currently unavailable.

Clear cell renal cell carcinoma (ccRCC) has been shown to be a highly immune-infiltrated tumor in multiple clinical and genomic studies [15, 18]. A recent study found that cytolytic activity index (CYT), defined as the geometric average of *GZMA* and *PRF1* expression, was the highest in ccRCC when compared to 17 other human cancers [13]. The spontaneous regression seen in up to 1% of ccRCC cases is also thought to be largely immune-mediated [19]. Additionally, ccRCC was historically one of the first malignancies to respond to immunotherapy and continues to be among the most responsive [20–23]. However, the mechanisms underlying high immune infiltration, spontaneous remissions, and response to immunotherapy in this malignancy remain poorly understood.

The success of immune checkpoint blockade in melanoma and non-small cell lung carcinoma (NSCLC) has largely been attributed to the high mutation burden in these tumors [10, 11]. A higher number of tumor mutations is expected to result in greater numbers of MHC binding neo-antigens, which have been proposed to drive tumor immune-infiltration and response to immunotherapy [9, 10, 13, 24–26]. However, the modest mutation load of ccRCC compared with other immunotherapy-responsive tumor types [27] challenges the notion that neo-antigens alone can drive immune infiltration and response to immunotherapy in these tumors.

As depicted in the workflow in Additional file 1: Figure S1a, we employed 24 immune cell type-specific gene signatures from Bindea et al. [14] (Additional file 1: Figure S1b) to computationally infer the infiltration levels in tumor samples (Step 1). We validated the gene signatures and our inference methodology using a ccRCC cohort from our institution (Step 2). We then defined a T cell infiltration score (TIS), an overall immune infiltration score (IIS), and an APM score to highlight the immune response differences between ccRCC [28] and 18 other tumor types profiled by The Cancer Genome Atlas (TCGA) research network (Step 3). Next, we characterized the immune-infiltration patterns in ccRCC patients by using the levels of 24 immune cells, angiogenesis, and expression of immunotherapeutic targets such as PD-1, PD-L1, and CTLA-4 (Step 4). We then interrogated the impact of geographic intratumoral heterogeneity and clonality on immune infiltration. Next, we investigated a suite of mechanisms that could potentially drive tumor immune-infiltration and explain the observed infiltration patterns in ccRCC. We validated our findings in an independent multi-platform ccRCC dataset [29] (Step 5). Finally, in a small series of Nivolumab-treated patients, we observed that our signatures correlate with response to checkpoint blockade therapy in ccRCC (Step 6). This integrative study utilizing rich whole-exome, whole-transcriptome, proteomic, and clinical data substantially improves our understanding of the tumor microenvironment in ccRCC and establishes an approach that can easily be extended to other human cancers.

## Results

### In silico decomposition of the tumor-immune microenvironment

We quantified the relative tumor infiltration levels of 24 immune cell types by interrogating expression levels of genes in published signature gene lists [14]. The signatures we used comprised a diverse set of adaptive and innate immune cell types and contained 509 genes in total (Additional file 2: Table S1). Of these genes, 98.4% (501) were used uniquely in only one signature (Additional file 1: Figure S2). Due to the interconnectedness between immune cell infiltration and the antigen presenting machinery (APM), we also defined a seven-gene APM signature that consisted of MHC class I genes (HLA-A/B/C, B2M) and genes involved in processing and loading antigens (TAP1, TAP2, and TAPBP). Messenger RNA (mRNA)-based scores for these signatures were computed separately for each sample using ssGSEA [30]. ssGSEA measures the per sample overexpression level of a particular gene list by comparing the ranks of the genes in the gene list with those of all other genes.

We employed this approach to computationally assess the infiltration levels of immune cell types and APM gene expression levels in 7567 tumor and 633 normal samples from 19 different cancer types profiled by TCGA (Additional file 2: Table S2). To achieve a more focused view of the immune infiltration landscape in human cancers, we defined two aggregate scores: (1) the overall immune infiltration score (IIS) from both adaptive and innate immune cell scores; and (2) the T cell infiltration score (TIS) from nine T cell scores (CD8^+^ T, Th1, Th2, Th17, Treg, T effector memory, T central memory, T helper, and T cells) (see “Methods”). We computed the TIS and IIS of each sample in the study as the sum of the relevant individual scores.

### Validation of the immune cell scoring methodology

Immune cell gene signatures were established by Bindea et al. [14] using three gene expression datasets [31–33] generated from sorted immune cell populations. Before validating these signatures on independent datasets, we first sought to confirm their discriminatory power on the datasets used to establish them and asked whether the expression of these genes separated immune cell populations into groups that were consistent with hematopoietic lineages. To this end, we obtained the microarray expression values for these genes, normalized with GCRMA [34] and corrected for batch effects using ComBat [35] (Additional file 1: Figure S3, see “Methods”). We then computed the principal components (PCs) of the batch-effect corrected dataset as a linear combination of the sorted immune cell types. This PC analysis successfully separated the cells into groups consistent with their hematopoietic lineage, suggesting adequate discrimination power for the signature genes (Additional file 1: Figure S4). More specifically, PC1 and PC2 achieved the separation of the following four groups: (1) macrophages and dendritic cells (DC); (2) B cells, NK cells (CD56dim and CD56 bright), CD8^+^, and CD4^+^ T cells; (3) Th1, Th2, T gamma delta, and T follicular helper cells; (4) mast cells, neutrophils, and eosinophils. The separation between CD8^+^ and CD4^+^ T cells was greatly enhanced if batch effect correction and PC analysis were performed with only the signatures genes of sorted T cell subpopulations (Additional file 1: Figure S5, see “Methods”).

**Fig. 1:**
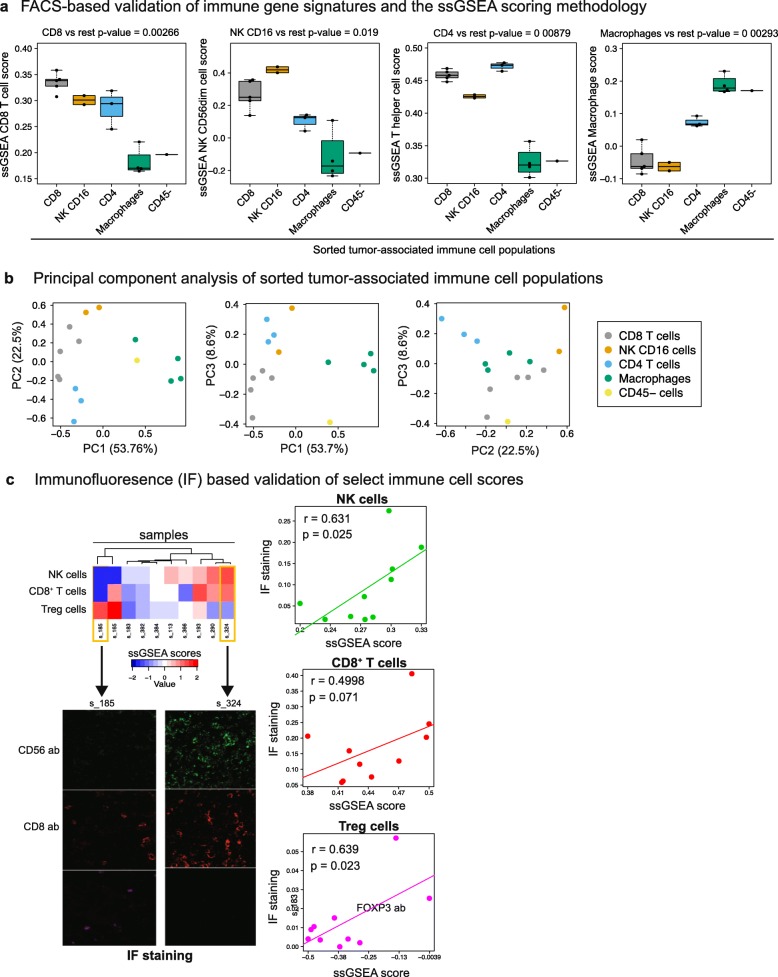
In vitro validation of the immune cell scoring method. **a** Immune cell populations were sorted from ccRCC patient specimens, and profiled for RNA-Seq gene expression. ssGSEA scores were computed for each sample using Bindea et al. signatures. Each ssGSEA score was the highest for the corresponding tumor-associated immune cell population and also had a significant difference from the other sorted populations (*p* values are provided above each figure). **b** Principal component analysis (PCA) of sorted tumor-associated immune cell populations. PCs were computed as a linear combination of 29 immune microenvironment variables (Additional file 2: Table S3). **c** Immunofluorescence (IF) validation of ssGSEA scores in an MSKCC cohort. The *top left panel* shows the unsupervised clustering of ssGSEA scores for NK, *CD*8^+^ T, and Treg cells in the 10 patients. IF staining for two samples at the opposite ends of the *heatmap* is shown in the *bottom left panel* (CD56, CD8, and FOXP3 antibodies respectively). The association of the immune infiltrate levels inferred by these two orthogonal methods (ssGSEA and IF) is shown in the *right panel*. The IF score (*y-axis*) represents the ratio of CD56, CD8, and FOXP3 positive cells versus total cells (DAPI-stained) for a given sample and was determined as the average across three representative regions on the slide

Next, we validated the gene signatures and the ssGSEA methodology in a series of in vitro and in silico tests. The first test involved sorting immune cell populations with fluorescence activated cell sorting (FACS) and generating RNA-Seq gene expression profiles of the sorted populations. To this end, we obtained ccRCC patient specimens and sorted prevalent tumor-infiltrating immune populations such as CD8^+^ T cells (n = 5), NK CD16^+^ cells (n = 2), CD4^+^ T cells (n = 3), and macrophages (n = 4) as well as non-immune CD45^–^ cells (n = 1). We then generated ssGSEA scores for all sorted samples using Bindea et al. signatures (Additional file 2: Table S3) and observed that each signature (CD8^+^ T cell, NK CD56dim cell, T helper cell, and macrophage signature, respectively) was able to identify the corresponding sorted population as being significantly higher than the other sorted populations (Fig. 1a) (Note that NK CD16^+^ cells are equivalent to NK CD56dim cells). Expectedly, the magnitude of the difference between the first and second highest immune population varied as a function of the phenotypic difference between the two populations. For instance, CD8^+^ T cells were most similar to NK CD16^+^ cells, another immune population with cytotoxic properties. Nevertheless, the first three PCs of ssGSEA scores were able to distinguish all tumor-associated immune populations as distinct clusters (Fig. 1b, Additional file 2: Table S3).

The second in vitro validation test involved comparing mRNA-based ssGSEA scores with levels of immunofluorescence(IF)-stained immune cells from 10 MSKCC primary ccRCC tumors (see “Methods” for sample preparation). IF staining was performed for three immune cell types that are extensively studied with immunohistochemistry: CD8^+^ T cells (anti-CD8 antibody), natural killer (NK) cells (anti-CD56 antibody), and regulatory T cells (Tregs) (anti-FOXP3 antibody). Notwithstanding that IF is a semi-quantitative technique, we observed significant correlations between IF immune cell infiltration estimates and ssGSEA scores (Fig. 1c). The Spearman correlation for the NK, Treg and CD8^+^ T cell populations were 0.631 (*p* = 0.025), 0.639 (*p* = 0.023), and 0.4998 (*p* = 0.071), respectively. Higher correlation levels may be precluded by the spatial heterogeneity of immune cell infiltrates and random sampling effects between the tissue sections used for IF staining and RNA-Seq.

**Fig. 2:**
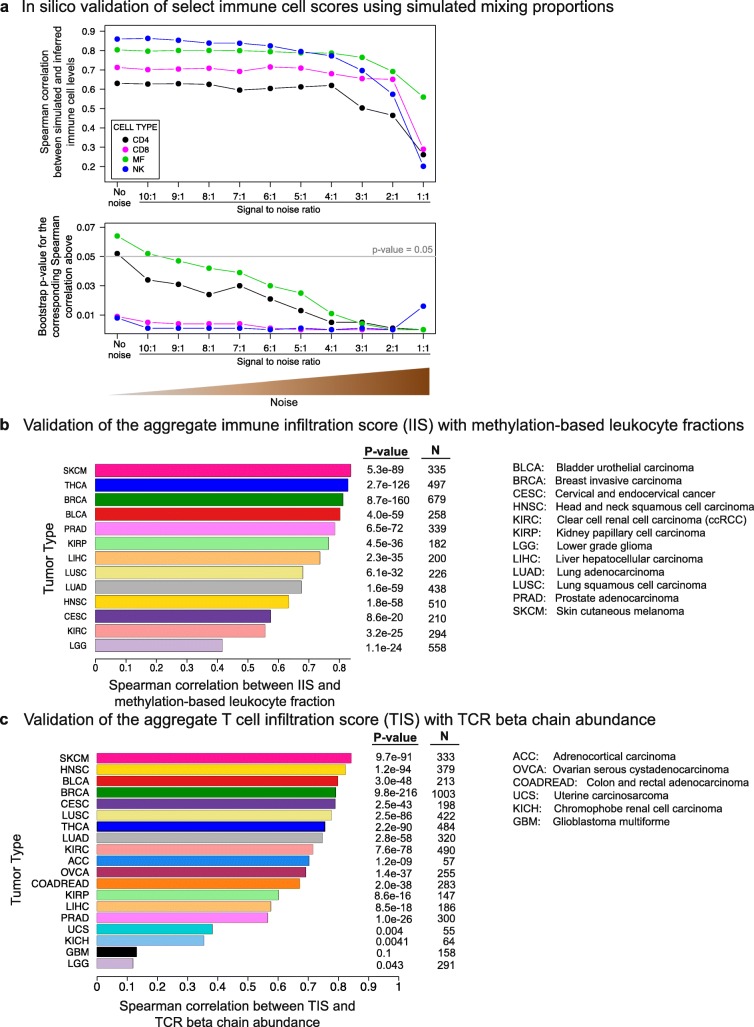
In silico validation of the immune cell scoring method. **a** In silico validation of immune cell scores using simulated mixing proportions. RNA-Seq profiles of FACS-sorted NK cells, macrophages, *CD*4^+^ and *CD*8^+^ T cells, and non-immune *CD*45^–^ cells were mixed with known proportions to obtain a “clean” mixture. Noise was added at varying SNRs. Mixing levels were then inferred by ssGSEA from the “clean” and noisy mixtures. The Spearman correlations between the simulated and inferred levels (*top panel*) and the bootstrap *p* values for these correlation values (*bottom panel*) are shown on the *y-axes* (Additional file 1: Figure S18 and “Methods” for the calculation of the bootstrap *p* values). **b** Validation of IIS with methylation-based leukocyte fractions. Spearman correlations between the two orthogonal scores are shown on the *x-axis* for 13 tumor types. **c** Validation of TIS with TCR beta chain abundance. Both scores are computationally inferred from RNA-Seq data but employ different approaches to measure T cell levels. Spearman correlations are shown on the *x-axis* for 19 tumor types

We next performed an in silico validation test to ask whether our methodology could successfully infer simulated, i.e. known, mixing proportions of immune cell types at varying noise levels. To this end, we first utilized the RNA-Seq data from sorted tumor-infiltrating cells and generated a reference expression profile for each one of the sorted immune cell populations (CD8^+^ T cells, NK CD16^+^ cells, CD4^+^ T cells, and macrophages) as well as for non-immune CD45^–^ cells (see “Methods”). Next, we simulated the tumor microenvironment by linearly mixing these five reference RNA-Seq profiles: The mixing proportions used in the linear combinations summed to 1 and were simulated from a uniform (0,1) distribution. Two hundred in silico mixture samples obtained in this manner formed the “clean” (i.e. no noise) dataset. To obtain the “noisy” datasets, Gaussian noise was added at signal-to-noise ratios (SNR) ranging from a slightly noisy 10:1 to an extremely noisy 1:2 SNR. Two hundred samples were generated at each noise level. ssGSEA was then run on all mixture samples with the CD8^+^ T, T helper, macrophage, and NK CD56dim signatures from the Bindea et al. set. We observed that the Spearman correlations between the simulated and inferred mixing levels remained stable and above 0.6 for all four cell types (bootstrap *p* values < 0.05, see “Methods”) in a long SNR range from 9:1 to 4:1 (Fig. 2a). Given the low noise levels of RNA-Seq relative to microarrays, the actual SNR in an RNA-Seq experiment would likely not be lower than 4:1. Thus, the SNR analysis indicated that ssGSEA-based immune decomposition is robust to the potential technical and/or experimental sources of noise in the system.

The second in silico test involved the validation of the two aggregate scores: IIS and TIS. IIS was validated with leukocyte fractions computationally inferred from available TCGA DNA methylation data in 13 cancer types (see “Methods”). The fractions obtained using this orthogonal data type were highly concordant with the RNA-Seq based IIS. Out of 13 tumor types, 10 exhibited Spearman correlations greater than 0.6 and all 13 had highly significant *p* values (Fig. 2b, Additional file 1: Figure S6 left column). As expected, IIS levels were often strongly negatively correlated with tumor purity as inferred by ABSOLUTE [36] (Additional file 1: Figure S6 right column). The other aggregate score utilized in this study, TIS, was validated with T cell receptor (TCR) beta chain abundance data computationally inferred from RNA-Seq data in [37]. Out of the 19 tested cancer types, 17 had highly significant correlation values (brain cancers GBM and LGG did not), the majority of which were greater than 0.6 (Fig. 2c, Additional file 1: Figure S7).

We attempted to compare the immune cell scores from CIBERSORT [16] with our ssGSEA scores (see “Methods”) even though CIBERSORT has not yet been validated for RNA-Seq data. We observed that CIBERSORT returned zero for the majority of samples in multiple cell types, whereas ssGSEA by design returns approximately Gaussian values for any signature. This difference coupled with the differences in cell sorting strategies led to poor or moderate correlations for the majority of immune cell populations (Additional file 2: Table S9). In cases where CIBERSORT did not return zeroes and Bindea et al. were attempting to describe the same cells, we observed relatively stronger levels of concordance (CD8 T cells, T follicular helper cells, and Tregs; Pearson r = 0.725, 0.395, 0.353; *p* value = 6.9e-33, 1.2e-8, 4.6e-7 respectively) (Additional file 2: Table S9).

These independent validation results show that our in silico decomposition is a reliable method to infer immune infiltration levels in tumor samples.

### The T cell infiltration spectrum across 19 human cancer types

**Fig. 3:**
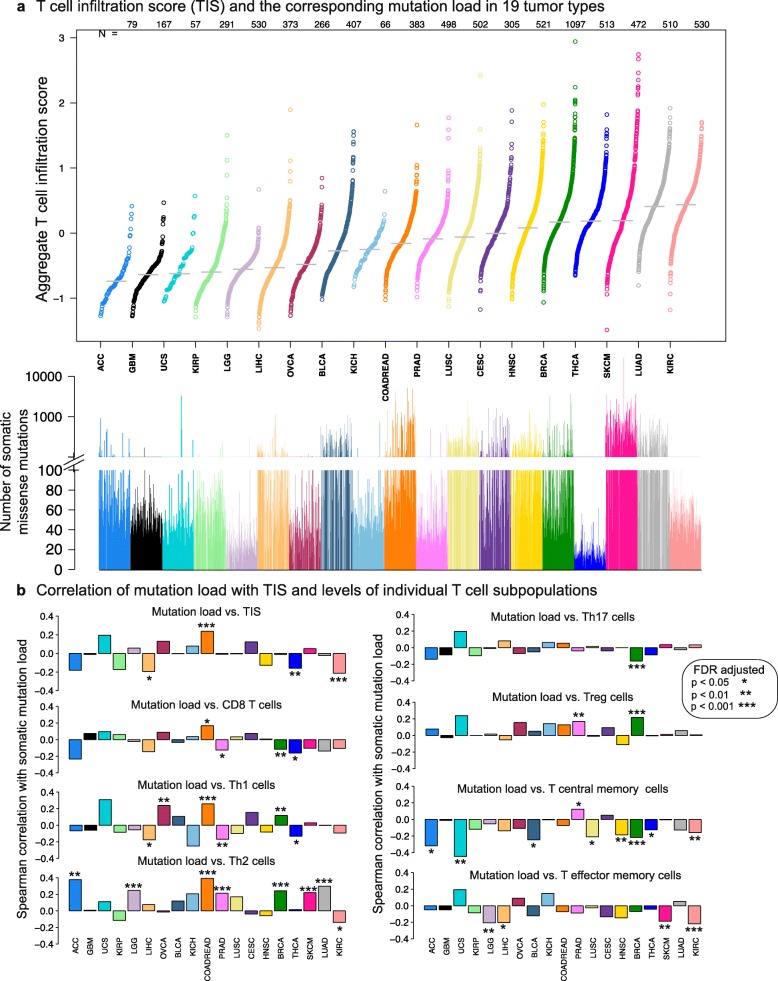
Analysis of T cell infiltration in 19 tumor types. **a** T cell infiltration scores (TIS) and the corresponding mutation load in 19 tumor types. TIS is an aggregate score obtained as the average of nine distinct T cell subset scores (*CD*8^+^ T, Th1, Th2, Th17, Treg, T effector memory, T central memory, T helper, and T cells). Each *circle* in the *top panel* shows the TIS for a tumor sample. In the *bottom panel*, the *vertical bar* corresponding to each *circle* shows the number of somatic missense mutations. Tumor types are ordered from *left* to *right* according to increasing median TIS (medians indicated by *horizontal gray bars*). **b** Correlation of mutation load with TIS and levels of individual T cell subpopulations. Spearman correlation coefficients are computed between number of somatic missense mutations and ssGSEA-based immune cell infiltration levels. Coefficients are plotted on the *y-axis* in *bar plots* and *asterisks* are added to indicate level of significance, as denoted in the *legend*. Tumor types are ordered in the same order as in Fig. 3a

The TIS and IIS of each sample in the 19 studied cancer types were computed as the sum of the individual scores from the relevant immune subpopulations. We observed that ccRCC and lung adenocarcinoma (LUAD) represented the highest end of the TIS and IIS spectrum (Fig. 3a for TIS and Additional file 1: Figure S8 for IIS). A pan-cancer view of the levels of individual T cell subpopulations that make up the TIS variable is presented in Additional file 1: Figure S9.

Missense mutations within tumor cells are a known source of neo-antigens that can initiate a T cell dependent immune response [38]. Previous studies have reported a significant correlation between “total number of mutations” and cytolytic activity index (CYT) in a pan-cancer context [13]. However, synonymous mutations do not give rise to neo-antigens, therefore the correlations between “number of missense mutations” and CYT are more relevant to study when investigating the immunogenicity of tumor types. We observed that, across 18 cancer types, only glioma and stomach adenocarcinoma had significant correlations between CYT and number of missense mutations after correction for multiple hypothesis testing (Additional file 1: Figure S10c). When only the 5th to the 95th percentile of the missense mutation counts was used as implemented in [13], the number of cancer types with significant CYT versus mutation count correlations increased to a modest four (Additional file 1: Figure S10d).

Consistent with CYT findings, we also observed a lack of consistent positive pan-cancer correlations between TIS levels in tumors and the corresponding numbers of somatic missense mutations (Fig. 3b, top left panel). On the contrary, there was a greater number of tumor types with significant negative correlations between these two variables; an observation which held true for CD8, central memory and effector memory T cells as well (Fig. 3b). One notable exception was colorectal adenocarcinoma (COADREAD) where the hypermutated subpopulation had elevated levels of TIS (r = 0.303, *p* value = 3.6 × 10^–7^, n = 271) [39] (Fig. 3a). It is not obvious whether the negative correlations arise due to a direct relationship between mutated neoepitopes and T cells or due to an unknown confounding variable. We speculate that immunoedited [40] tumors which have gone through equilibrium and escape can lead to divergence in the association between mutation burden and T cell infiltration. For instance, tumors which acquire the ability to suppress T cell activation may continue to accumulate mutations as immune infiltration decreases.

In contrast to CD8 and memory T cells, Th2 and Treg cell levels generally showed a positive correlation with mutation load (Fig. 3b). These correlations could be indicative of an immunosuppressive environment enriched in Treg and/or Th2 cells where tumors have escaped elimination by the immune system despite bearing a large number of potentially immunogenic mutations.

**Fig. 4:**
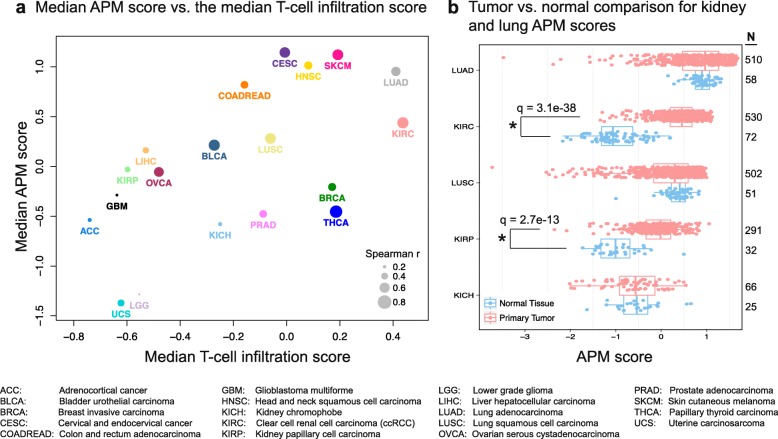
Pan-cancer analysis of TIS association with antigen presenting machinery (APM) gene expression. **a** The association between the median APM score and the median T cell infiltration score across 19 tumor types. The sizes of the *circles* are proportional to the within-cohort Spearman correlation between TIS score and APM score. KIRC and LUAD are among the highest not only for APM score but also for the APM–TIS correlation. **b** The APM score differences between tumors and adjacent normal tissue in kidney and lung neoplasms. Each *circle* is the APM score of a tumor (*red*) or an adjacent normal (*blue*) sample. No significant tumor-normal differences are observed in lung adenocarcinoma (LUAD), lung squamous cell carcinoma (LUSC), or kidney chromophobe (KICH) at α = 0.05. However, clear cell and papillary renal cell carcinoma (KIRC and KIRP) tumors significantly overexpress APM genes. The Benjamini–Hochberg adjusted *p* values are reported in the *figure* (Mann–Whitney test)

Immune infiltration is expected to increase the expression of APM genes in the tumor through paracrine signaling and mRNA generated by the infiltrating cells. Therefore, we investigated the correlation between the TIS and APM scores across the tested tumor types. As expected, the median TIS and the median APM score in the 19 cohorts showed a strong correlation (Spearman r = 0.611, *p* = 5.5 × 10^–3^), where ccRCC and LUAD were again among the highest with respect to the within-cohort TIS-APM correlation (Fig. 4a). Cancer types with low within-cohort correlations included GBM, LGG, ACC, and KICH. APM levels in these cancer types are indeed most strongly correlated with macrophages or subpopulations of dendritic cells (activated, immature, or total DCs) (Additional file 1: Figure S24).

Interestingly, a comparison of the APM expression between the tumor and normal tissue for kidney (clear cell, chromophobe, and papillary sub-histologies) and non-small cell lung tumors (adenocarcinoma and squamous cell) revealed that the tumor-normal difference was highly significant for ccRCC (q = 3.1 × 10^–38^, Mann–Whitney test) and papillary RCC (q = 2.7 × 10^–13^, Mann–Whitney test) but not significant for other tumor types (Fig. 4b). Notably, the tumor-normal difference for the APM score was the most pronounced in ccRCC compared with 14 other cancer types (Additional file 1: Figure S11) (no normal samples were available in the TCGA dataset for the other cancers). APM expression of ccRCC tumors did not show a positive association with either grade (Spearman r = –0.11, *p* = 0.02, n = 421) or stage (Spearman r = –0.14, *p* = 0.004, n = 422). Moreover, the grade-specific and stage-specific differences of APM expression levels were weak (*p* = 0.0704 and 0.0037, respectively, ANOVA) (Additional file 1: Figure S12). These results indicate that APM upregulation in ccRCC is likely an intrinsic ccRCC phenomenon and not dependent of tumor necrosis or other features associated with aggressive disease.

In a survey of the other immune cell types, we found that the unique features of ccRCC immune infiltration extends to high levels of CD8^+^ T cells, plasmacytoid DCs (pDC), T cells, cytotoxic cells, and neutrophils; and low levels of Th2 and Treg cells compared with the other 18 cancer types (Additional file 1: Figure S13).

### Immune-infiltrate decomposition in ccRCC reveals three distinct patient clusters

In our effort to characterize the microenvironment of ccRCC tumors, we expanded our repertoire of 24 immune cell types to also include an angiogenesis signature [41] (Additional file 2: Table S1) and immunotherapeutic targets PD-1 (*PDCD1*), PD-L1 (*CD274*), and CTLA-4 (*CTLA4*). Angiogenesis is well established to be a characteristic component of immune inflammation [42] and ccRCC is known to have high angiogenic capacity due to constitutive activation of the hypoxia-inducible factor pathway [43]. We confirmed the high angiogenesis levels in ccRCC via a comparison against 18 other tumor types explored in this study (Additional file 1: Figure S13).

**Fig. 5:**
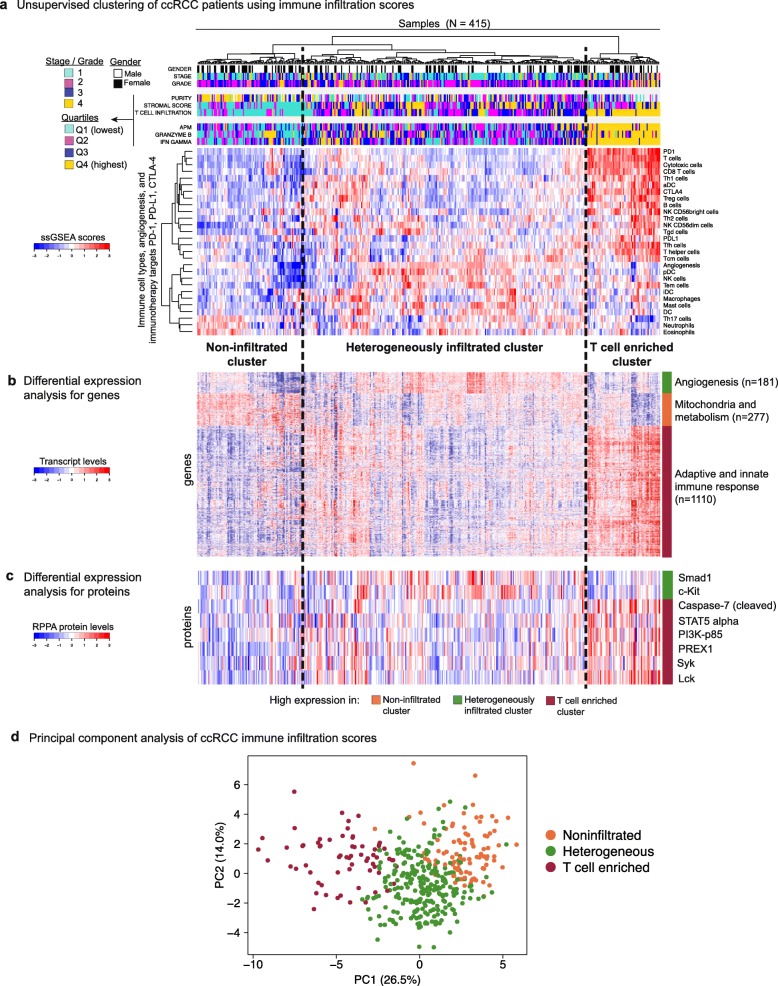
Characterization of immune infiltration clusters in ccRCC. **a** Unsupervised clustering of 415 ccRCC patients from the TCGA cohort using ssGSEA scores from 24 immune cell types, three immunotherapy targets (PD-1, PD-L1, CTLA-4), and angiogenesis. Hierarchical clustering was performed with Euclidean distance and Ward linkage. We discover three distinct immune infiltration clusters, here termed (1) non-infiltrated, (2) heterogeneously infiltrated, and (3) T cell enriched. The T cell enriched cluster is characterized by tumors with high APM scores and high granzyme B and interferon gamma mRNA expression levels. **b** Differential expression analysis with Mann–Whitney test for all genes in the TCGA RNA-Seq dataset excluding signature genes. Only genes that are significantly overexpressed in one cluster at a q-value cutoff of 5 × 10^–5^ are shown. Pathway analysis using DAVID [44] reveals that the genes overexpressed in the three clusters (n = 1110, 181, and 277, respectively) are enriched in (1) adaptive and innate immune response, (2) angiogenesis, and (3) mitochondrial and metabolic processes. **c** Differential expression analysis with Mann–Whitney test for all proteins in the TCGA reverse phase protein array (RPPA) dataset. Only proteins that are significantly overexpressed in one cluster at a q-value cutoff of 0.01 are shown. This analysis recapitulates the significant differences in immune response in the T cell enriched cluster and in angiogenesis in the heterogeneously infiltrated cluster. **d** PCA of the immune infiltration scores in ccRCC. The three clusters most likely reflect distinct biology

**Fig. 6:**
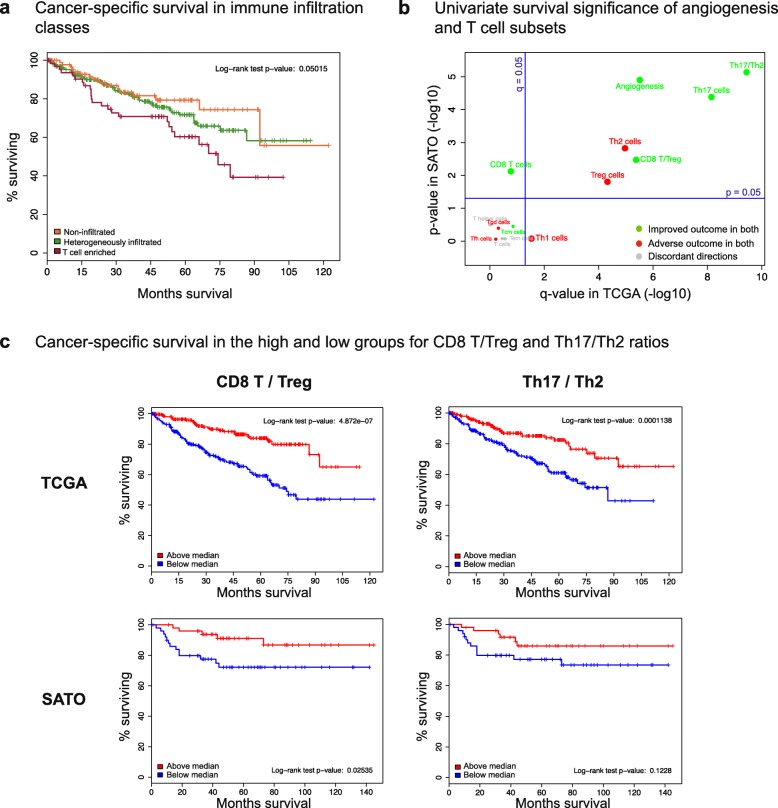
Prognostic significance of ccRCC immune infiltration classes and distinct T cell subsets. **a** Kaplan–Meier curves for cancer-specific survival in ccRCC immune infiltration classes. The T cell enriched class has the poorest survival whereas the non-infiltrated class is associated with better outcomes (log-rank test *p* value = 0.05). **b** Prognostic significance of angiogenesis and distinct T cell subsets in ccRCC. Univariate Cox proportional-hazards was used to regress ssGSEA scores on cancer-specific survival. The resultant *p* values in the TCGA dataset were adjusted for multiple hypothesis testing, log-transformed, and then plotted against the log-transformed *p* values from the SATO dataset. Survival associations concordant in both datasets are denoted in *green* and *red* for improved and poor outcome respectively. Discordant associations are denoted in *gray. P* values from the SATO dataset are not adjusted for multiple hypothesis testing since this is the validation cohort. **c** Kaplan–Meier curves for cancer-specific survival in the above-median and below-median groups for the *CD*8^+^ T/Treg and Th17/Th2 ratios. The median values for these two ratios are able to stratify both the TCGA and the SATO cohorts into groups with significant survival differences

Using the ssGSEA scores from the expanded panel of 28 immune-related and inflammation-related gene signatures, we performed unsupervised clustering on the TCGA cohort of 415 patients (see “Methods”). Strikingly, this analysis revealed three distinct clusters that predominantly separated according to levels of T cell infiltration and APM gene expression, here termed the (1) T cell enriched (n = 65, 15.7%), (2) heterogeneously infiltrated (n = 257, 61.9%), and (3) non-infiltrated clusters (n = 93, 22.4%) (Fig. 5a). We observed that the T cell enriched tumors had markedly high expression of granzyme B (*GZMB*) and interferon-gamma (*IFNG*), effector molecules prominently associated with T cell response. Despite high levels of T cell infiltration and effector molecules, patients in the T cell enriched class had the poorest cancer-specific survival whereas the non-infiltrated group fared the best (*p* = 0.05; log-rank test) (Fig. 6a). Coupled with the observation that inhibitory checkpoint molecules PD-1 and CTLA-4 are also expressed at high levels in the T cell enriched class, this finding suggests that effector T cells in the tumor microenvironment may not be able to exert their pro-survival effects due to being offset by inhibitory cells/molecules and factors such as exhaustion and/or anergy.

An orthogonal measurement of tumor purity by the DNA-based ABSOLUTE algorithm [36] confirmed that the non-infiltrated group was the purest cluster (mean 0.640) and the T cell enriched group was the least pure cluster (mean 0.436) (*p* < 2 × 10^–16^, ANOVA). We then assessed the stromal content of samples using the RNA-based ESTIMATE algorithm [15] and investigated its association with the clusters. We found that the non-infiltrated cluster demonstrated the lowest stromal scores whereas the heterogeneous and T cell enriched clusters displayed mixed degrees of stromal content (*p* = 4 × 10^–7^, ANOVA).

In order to validate that the three immune infiltration clusters are not unique to the TCGA ccRCC cohort, we utilized a separate publicly available dataset of 101 ccRCC tumors for which comparable multi-platform data were available [29] and refer to it as the SATO dataset from here on. A random forest classifier was trained on the TCGA cohort using the ssGSEA scores of 28 immune-related variables. This classifier was used to predict the immune infiltration class for each SATO patient (see “Methods”). The heatmap of the same 28 immune features in the SATO dataset confirmed the existence of the three classes as well as the elevated expression levels of APM, granzyme B, and interferon-gamma in the T cell enriched cluster (Additional file 1: Figure S14a).

To further characterize the clusters’ unique molecular features, we next performed an unbiased analysis of differential gene and protein expression between the clusters. We excluded the signature genes and performed pathway analysis [44] for the genes significantly overexpressed in one of the clusters (q < 5 × 10^–5^, Mann–Whitney test). We observed that the T cell enriched group had significant overexpression of both adaptive and innate immunity genes (Fig. 5b and Additional file 2: Table S4A). On the other hand, the non-infiltrated group had significant overexpression of metabolism-related and mitochondria-related genes (Additional file 2: Table S4B), while the heterogeneously infiltrated group had overexpression of angiogenesis-related genes (Additional file 2: Table S4C) (q < 5 × 10^–5^, Mann–Whitney test). These findings were again validated in the SATO dataset (Additional file 1: Figure S14b, Additional file 2: Table S5A–C). We next utilized the TCGA reverse phase protein array (RPPA) dataset for the differential protein expression analysis. We consistently observed overexpression of immune-related proteins, such as Lck and Syk, for the T cell enriched group; and an overexpression of angiogenesis-related proteins, such as Smad1 [45, 46] and c-Kit [47–49], for the heterogeneously infiltrated group (q < 0.01, Mann–Whitney test) (Fig. 5c). A proteomic dataset for the SATO cohort was not available.

PCA on the ccRCC immune infiltration scores showed that the three clusters defined above cannot be explained by a one-dimensional infiltration gradient and most likely reflect distinct biology (Fig. 5d). Even though non-infiltrated and heterogeneously infiltrated tumors are not as well distinguished from each other as they are from the T cell enriched group, the evidence from differential gene and protein expression analyses indicate that these clusters are likely distinct as they have unique biology with respect to pathways such as those in angiogenesis and mitochondria/metabolism.

The T cell enriched cluster in the TCGA dataset exhibited two subclusters, here termed TCa (n = 39, 60%) and TCb (n = 26, 40%) (Additional file 1: Figure S15a), with different immune cell infiltration and gene expression profiles. Gene set enrichment analysis with DAVID [44] and ClueGO [50] revealed that the genes overexpressed in TCa (q < 5 × 10^–5^, Mann–Whitney test) were associated with metabolic and mitochondrial processes (Additional file 1: Figure S15b, Additional file 2: Table S5A). The genes overexpressed in TCb (q < 5 × 10^–5^, Mann–Whitney test) were enriched for processes related to cell cycle, extracellular matrix (ECM), and cellular proliferation (Additional file 1: Figure S15b, Additional file 2: Table S5B). We also found that these two subclusters had prognostic differences (Additional file 1: Figure S15c), with the TCb patients having worse cancer-specific survival than the TCa patients (*p* = 0.0162, log-rank test). Moreover, the TCb subcluster had significantly higher macrophage infiltration (*p* = 5.7 × 10^–4^) and stromal score (*p* = 4.6 × 10^–4^, Mann–Whitney test) with a moderate correlation between these two variables (Spearman r = 0.418, *p* = 5.8 × 10^–4^, n = 65). This correlation generalized to the entire TCGA ccRCC cohort (Spearman r = 0.561, *p* < 2 × 10^–16^, n = 415), suggesting the possibility of macrophage recruitment by stromal cells [51] (Additional file 1: Figure S16). These results confirm the biologically distinct characteristics of the TCa and TCb subclusters within the T cell enriched group.

### T cell infiltration levels are associated with clinical outcomes

We found that tumor immune-infiltration in ccRCC was associated with distinct clinicopathologic features. Male patients (*p* = 0.018), higher stage (*p* = 0.006), and higher grade (*p* = 0.003) tumors were over-represented in the T cell enriched class compared to the non-infiltrated and heterogeneously-infiltrated groups (Fisher’s exact test). We next investigated the univariate significance of each T cell subset and angiogenesis as a predictor of cancer-specific survival. Cox proportional-hazards regression showed that, in both the TCGA (n = 415) and SATO (n = 101) datasets, the levels of Th17 cells and angiogenesis were strongly associated with favorable outcomes, whereas Th2 and Treg cells were associated with adverse outcomes (Fig. 6b) consistent with previous reports [18, 41, 52–55]. To optimize prognostic discrimination, we explored Th17 ratios with other immune subtypes and identified the Th17/Th2 ratio as the most predictive in both the TCGA and SATO cohorts (Fig. 6b, c). Moreover, we observed that CD8^+^ T cell levels alone were not significantly associated with improved survival in the TCGA cohort, but the frequently used CD8^+^ T/Treg ratio was (Fig. 6b, c).

Additional analyses demonstrated that previously identified prognostic features such as tumor stage and molecular ccRCC subtype (ccA/ccB) [56] were associated with similarly prognostic immune infiltration scores. In particular, Treg and Th17 infiltration levels had negative and positive association, respectively, with tumor stage (q = 6.1 × 10^–8^ for both, ANOVA) (Additional file 1: Figure S17). Treg and Th2 infiltration levels were higher in ccB (n = 175) subtype tumors (q = 3.9 × 10^–9^ and 1.2 × 10^–8^, Mann–Whitney test) compared with ccA (n = 205), which was previously shown to have better prognosis relative to ccB [56] (Additional file 1: Figure S18). In contrast, Th17 and CD8^+^ T cell infiltration levels were higher in ccA tumors (q = 2.8 × 10^–12^ and 5.8 × 10^–6^, Mann–Whitney test).

### Association of immune infiltration patterns with intratumor heterogeneity and subclonality

**Fig. 7:**
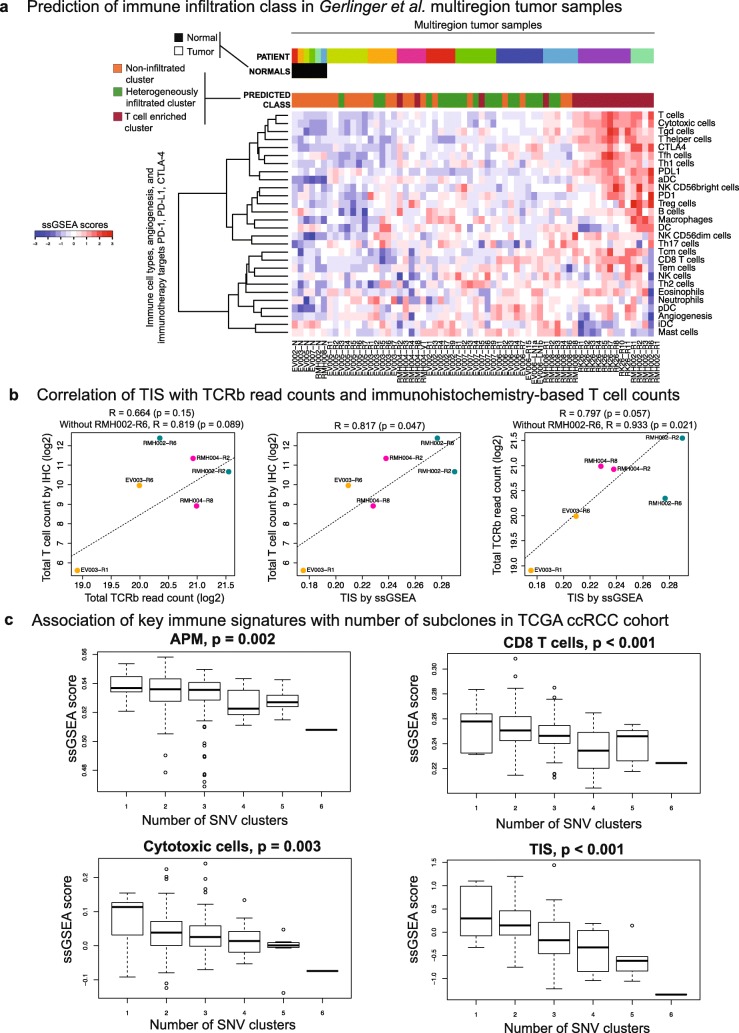
Association of ccRCC immune infiltration patterns with intratumor heterogeneity. **a** The immune infiltration class for each Gerlinger et al. multiregion tumor sample was predicted with a random forest classifier trained on the TCGA ccRCC cohort. The *y-axis* shows immune cell types and immunotherapy targets ordered according to Ward linkage in hierarchical clustering. The *x-axis* shows normal and multiregion tumor samples with a supervised order. Six normal samples are on the *far left* and tumor samples from each patient are grouped together. Patients are ordered according to increasing average infiltration level from *left* to *right*. Tumor samples within each patient are ordered according to alphabetical order. **b** Comparison of TIS with TCRb read counts and immunohistochemistry-based T cell counts. *Left*: The *scatter plot* and Pearson correlation of TCRb read counts with IHC-based T cell counts from [58] when restricted to the six samples that also have microarray expression data. A *linear regression line* is fitted through the data after exclusion of the outlier RMH002-R6 as in [58]. *Middle*: The *scatter plot* and Pearson correlation of IHC-based T cell counts with the ssGSEA-based aggregate TIS. A *linear regression line* is fitted through the data. *Right*: The *scatter plot* and Pearson correlation TCRb read counts with the ssGSEA-based aggregate TIS. A *linear regression line* is fitted through the data after exclusion of the outlier RMH002-R6. **c** SciClone clonality analysis for TCGA ccRCC samples. The *x-axis* shows the number of single nucleotide variant (SNV) clusters for each tumor where 1 corresponds to clonal tumors and higher number of clusters indicate subclonal architecture. *P* values are derived from trend tests between the number of SNV clusters and ssGSEA scores. The fraction of samples for each SNV cluster number is 4.6% for one cluster (n = 9), 55.7% for two clusters (n = 108), 27.8% for three clusters (n = 54), 7.7% for four clusters (n = 15), 3.6% for five clusters (n = 7), 0.5% for six clusters (n = 1)

We next investigated whether the immune infiltration classes predicted by our mRNA-based decomposition algorithm were robust to intratumoral heterogeneity. We obtained a microarray gene expression dataset from the Gerlinger et al. [57] ccRCC multiregion tumor study (referred to as GERLINGER from here on). This dataset includes 56 tumor and six normal samples from nine ccRCC patients. The authors sampled several tumor regions from each patient to investigate intratumor heterogeneity. We computed the ssGSEA-based immune cell infiltration scores and also the aggregate TIS for these samples, and applied the TCGA-based random forest classifier to predict the immune infiltration class for each sample (Fig. 7a). Interestingly, tumors with high T cell infiltration levels (RK26, RMH002) had highly similar immune infiltration profiles in most sampled regions; and all regions were predicted to be in the T cell enriched category. In contrast, tumors with relatively lower levels of T cells showed immune intratumor heterogeneity and had regions predicted to be in multiple different immune infiltration categories. For instance, regions in tumors RMH008 and EV007 were found to contain members in all three immune infiltration classes (T cell enriched, heterogeneously infiltrated, or non-infiltrated).

T cell receptor *β*-chain (TCRb) read counts from ultra-deep TCR sequencing and total T cell counts from immunohistochemistry (IHC) were also available for a subset of the GERLINGER microarray samples (n = 6) [58] (Additional file 2: Table S7). These two types of T cell abundance estimates have previously been shown to have a statistically significant correlation across 14 samples [58], despite RMH002-R6 being a strong outlier in terms of IHC-based T cell counts. We observed that the significance of the correlation was lost when the analysis was restricted to the six samples that also had microarray data (Fig. 7b, left panel) regardless of whether RMH002-R6 was included in the correlation computation (*p* = 0.15 and 0.089 with and without RMH002-R6, respectively). However, the ssGSEA-based TIS had at least borderline significant correlation with both of these variables despite the small number of samples (*p* = 0.047 for the correlation with IHC-based T cell counts and 0.057 for the correlation with total TCRb read counts) (Fig. 7b, middle and right panels). Moreover, the scatter plots with TIS interestingly showed that the IHC-based T cell count for RMH002-R6 was not an outlier (Fig. 7b, middle panel), but the total TCRb read count for the same sample was (Fig. 7b, right panel) (*p* value increases from 0.057 to 0.021 for the correlation between TIS and total TCRb read count when RMH002-R6 is removed). This finding suggested that the discordance of the T cell abundance estimates for this sample may not be due to an over-representation of T cells in the FFPE section as speculated in [58], but may be due to an underperformance of the steps involved in ultra-deep sequencing of TCRb reads from bulk tumor DNA. Yet, this is not certain as spatial heterogeneity of T cell infiltrates and random sampling effects confound all such comparisons.

A recent study on NSCLC reported an inverse relationship between T cell infiltration and subclonal architecture [59]. We performed clonality assessment on the TCGA ccRCC cohort using SciClone [60] (see “Clonality assessment” in “Methods”); and consistent with the NSCLC study, found that more clonal tumors (i.e. tumors with fewer subclones) had higher levels of CD8^+^ T cells, cytotoxic cells, APM, and TIS (Fig. 7c). Clonality for the SATO ccRCC cohort was also assessed using SciClone (see “Methods”) and the trends for the inverse association between immune infiltration and subclonal architecture were recapitulated in this dataset although *p* values did not reach significance and the trends were rather modest (Additional file 1: Figure S19). Both the TCGA and SATO results held true even when the immune scores were adjusted for purity (Additional file 1: Figure S20).

### Baseline elevation in TIS and APM in ccRCC patients responding to nivolumab

**Fig. 8:**
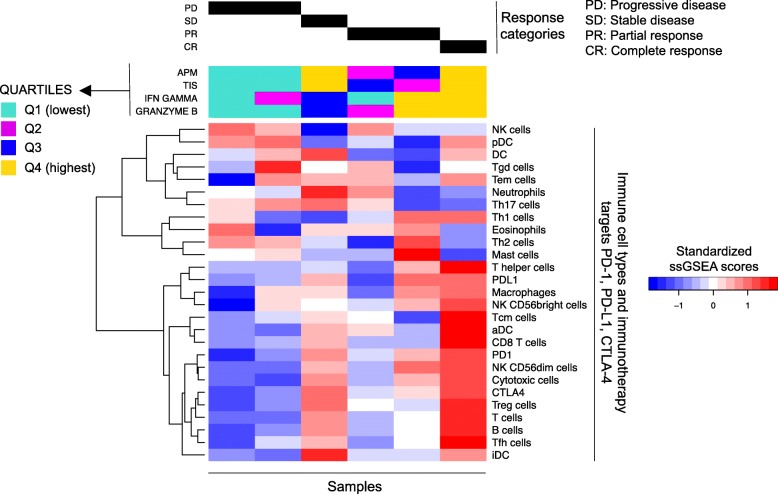
Immune infiltration profiles in nivolumab-treated ccRCC patients. RNA-Seq profiles of six ccRCC patients were generated and the patients were then treated with the checkpoint inhibitor nivolumab (anti-PD1). T cell infiltration as well as APM, IFNG, and GZMB levels are generally high in responders (complete response, partial response, or stable disease) and the highest levels are observed in the patient with complete response

Given the relationships we have identified between distinct immune cell subsets, APM, and clinical status, we next used RNA-Seq to ask whether there is a relationship between the baseline immune landscape and response to immunotherapy. Nivolumab (anti-PD-1) is FDA-approved for the treatment of advanced RCC, so we investigated the pretreatment immune profile of patients treated with this agent using a hypothesis-generating set of six patients. We found that both TIS and APM were elevated in responding patients (those with a partial or complete response to nivolumab) whereas they were in the lowest quartile for patients with progressive disease on nivolumab (Fig. 8). A similar pattern was observed when examining the relative expression of T cell effector genes IFNG and GZMB. This correlation should be substantiated in a larger cohort to determine if it has predictive power in determining response to PD-1 blockade.

### Association of immune infiltration with genomic alterations and neo-antigens

In light of our evidence suggesting the presence of immunologically distinct subsets of ccRCC tumors, we investigated mutation load and recurrent genomic alterations as potential drivers of the observed T cell infiltration. The tumors from the non-infiltrated class harbored slightly more somatic missense mutations than the T cell enriched class (the median number of somatic missense mutations in the non-infiltrated group was 36.5 versus 33 in the T cell enriched group; q = 0.07, ANOVA). Out of the 11 driver genes commonly mutated in ccRCC, only *PBRM1* was mutated at significantly different rates between the three populations (Additional file 1: Figure S21a; higher in non-enriched versus T cell enriched q = 0.04; higher in heterogeneous versus T cell enriched q = 0.04; Fisher’s exact). However, this observation was not validated in the SATO dataset. None of the common arm-level CNVs observed in ccRCC tumors were found at different rates between the three groups (Additional file 1: Figure S21b).

Cancer neo-antigens have been demonstrated to drive T cell infiltration of tumors in murine models of cancer [38, 61]. We hypothesized that the abundance or quality of cancer neo-antigens might differ between our tumor classes. To address this theory, we determined the HLA-A, HLA-B, and HLA-C alleles of each ccRCC TCGA patient using OptiType [62]. We then predicted the protein alterations expected to result from missense mutations in each tumor and identified those predicted to bind to MHC-I molecules (see “Methods”). We found no significant difference in the median MHC-I binding count (Additional file 1: Figure S22a) or median binding affinity (Additional file 1: Figure S22b) of neo-antigens between the three classes of TCGA tumors. We also found no significant difference in the fraction of tumors with non-silent somatic mutations in an expanded set of APM genes (Additional file 2: Table S8A-C). These results suggest that factors other than genomic alterations may be contributing to the immune infiltration of ccRCC tumors.

### ImmunExplorer web application

We have created a publicly available web application (http://kidneyimmune.chenghsiehlab.org/) that allows users to interactively visualize and perform integrated analysis of immune cell type levels, RNA-Seq, and clinical outcomes from the TCGA and Sato ccRCC datasets.

## Discussion

In this analysis, we present a computational approach based on overexpression of gene signatures for profiling the immune infiltration patterns in bulk tumor samples. Our methodology is different from deconvolution methods such as CIBERSORT [16] and DeconRNA-Seq [17] in that no regression or quadratic programming is involved, and only the ranks of the genes are used to infer relative cell levels. Hence, our approach does not “deconvolve” the mRNA expression data, but simply “decomposes” the immune infiltrate in the tumor microenvironment into levels of individual immune cell populations.

Compared to CIBERSORT and DeconRNA-Seq, our decomposition method has the advantages of (1) being compatible with both microarray and RNA-Seq platforms, and (2) not requiring reference expression vectors, which actually reduce the robustness of a method due to the fact that even small changes in the reference vectors may lead to substantial differences in the output when the deconvolution goal is cast into an optimization problem as in CIBERSORT and DeconRNA-Seq. Immune cell reference expression vectors are highly structured, static snapshots of the transcriptional programs of cell populations, and are highly likely to show inter-laboratory differences due to immune cells’ stimulation method, polarization state, activation state, exhaustion, or anergy. In contrast, the most abundantly expressed genes for a given cell type show little difference across different conditions. Therefore, gene signatures as in our approach offer a flexible yet principled approach to arrive at robust results.

One caveat to gene signatures is that two of them are defined by only a single gene in this study: Tregs (FOXP3) and pDCs (IL3RA). Decomposition of immune cell types with only few signatures genes is likely to be less robust than for immune cell types with many signature genes. Nevertheless, we observed that the pDC score was highly correlated with the angiogenesis score (a 40-gene signature) across many cancer types (Additional file 1: Figure S25) and this association has a known mechanism whereby pDCs induce angiogenesis [63]. Also, we were able to validate the Treg scores with immunofluorescence. Therefore, we feel confident that, despite the small number of genes, these signatures are tracking the intended cells.

Our results highlighted the immunotherapy-responsive tumors ccRCC and LUAD as having the highest T cell infiltration median. Moreover, ccRCC, but not LUAD, demonstrated significant upregulation of antigen presentation machinery in comparison with adjacent normal tissue. Preliminary evidence emerging from clinical trials of immune checkpoint blockade therapy suggests that high mutation burdens may be predictive of good responses in NSCLC and melanoma [10, 11]. However, ccRCC is another immunotherapy-responsive tumor despite bearing orders of magnitudes fewer mutations than NSCLC and melanoma. Our data suggest that ccRCC tumors may be responsive to checkpoint blockade because of a potent pre-existing immune infiltration and overall elevated level of antigen presentation and recognition.

Immune checkpoint blockade is generally thought to function by augmenting the activity of T cells subsequent to their priming by activated antigen presenting cells [64]. This suggests that “upstream” immunologic events (such as the stimulation of antigen presenting machinery) occur at baseline, resulting in primed anti-tumor T cells that are, in turn, made capable of controlling tumor growth upon treatment with immune checkpoint blockade. It is thus tempting to speculate that such events (resulting in effective antigen presentation) could be used to screen candidates for immunotherapy in the future. Our results on treatment response to the anti-PD-1 mAb nivolumab (Fig. 8) suggest the hypothesis that a pre-treatment analysis of antigen presenting machinery, and the corresponding T cell infiltrate, could be one method of achieving this. Given that PD-1 blocking mAbs are approved for a growing list of diverse cancers, such results could be applicable well beyond ccRCC.

Unsupervised clustering of ccRCC tumors using immune infiltration levels revealed three clusters of differentially infiltrated tumors, which were subsequently validated in an independent cohort. In particular, we found that the T cell enriched cluster was characterized by high expression levels of immune-response related genes including the immune checkpoint genes PD-1, PD-L1, and CTLA-4. Interestingly, a recent study also identified an aggressive, sunitinib-resistant molecular subtype of metastatic ccRCC with cellular and molecular characteristics similar to the T cell enriched tumors discovered here [65]. These findings across several cohorts of ccRCC patients suggest that a subset of ccRCC tumors may be both highly immune-infiltrated and immunosuppressed, as indicated by elevated expression of immune-checkpoint surface markers. Our findings also underscore the prognostic significance of specific T cell subsets, consistent with previous tissue-based studies of ccRCC and other tumor types [66].

Our in-depth analysis including driver mutations, CNVs, mutation burden, and neo-antigens was not able to identify any molecular mechanisms for the differential immune infiltration in ccRCC clusters. However, the lack of association between immune infiltration and predicted MHC-I binding tumor neo-antigens does not rule out neo-antigens as a driver of immune infiltration. Further, computational techniques for the prediction of immunogenic neo-antigens are not yet mature: most studies focusing on immunogenic epitopes remedy this shortcoming by using a combination of computational, biochemical, and cellular techniques. Others have suggested that the clonality of neo-antigens may drive immune recognition [59] and we consistently observed an inverse correlation between intratumor heterogeneity and immune infiltration in multiple datasets. An important caveat of the clonality analysis is that spatially segregated clones cannot be identified in the TCGA dataset. Overall, our results suggest that genetic alterations, mutation burden, and predicted neo-antigens currently provide an incomplete explanation for the degree of immune infiltration in ccRCC.

Our results illustrate the utility of ssGSEA for inferring immune infiltration levels in tumor specimens. The methodology in this study could directly be extended to the investigation of immune infiltration and its potential drivers in other tumor types and in various clinical settings including response to checkpoint blockade.

## Conclusions

In this study, we report that ccRCC is the most highly T cell infiltrated tumor type when compared with 18 other malignancies, and that the TIS as well as the expression levels of MHC class I APM have potential utility as biomarkers of response to PD-1 blockade therapy. It has previously been shown that ccRCC is an exception to the rule among immunotherapeutically responsive cancer types in that ccRCC has only a modest mutation load [27]. Here, we show for the first time that another unique feature of ccRCC is the upregulation of APM expression in tumor samples compared to adjacent normal tissue.

Our finding that the high T cell infiltration in the tumor persists throughout different geographic regions has possible translational relevance in terms of relying on a single core biopsy to characterize a tumor immune profile. We also present evidence that immune infiltration is negatively associated with number of subclones (lower ITH) in ccRCC, a finding consistent with a recent study [59] that observed the same phenomenon in lung carcinomas.

Ultimately, our approach enables the determination of a diverse array of immune infiltration patterns from small amounts of tissue such as biopsy samples; a strategy which could easily be incorporated into the clinical and trial setting.

## Methods

### Datasets

#### Gene and protein expression data

The pancan normalized gene-level RNA-Seq data for the TCGA cohorts were downloaded from the UC Santa Cruz Cancer Genomics Browser [67] (https://genome-cancer.ucsc.edu/). These cohorts consisted of adrenocortical cancer (ACC, *N*_*tumor*_ = 79, *N*_*normal*_ *=* 0), bladder urothelial carcinoma (BLCA, *N*_*tumor*_ = 407, *N*_*normal*_ *=* 19), lower grade glioma (LGG, *N*_*tumor*_ = 530, *N*_*normal*_ *=* 0), breast invasive carcinoma (BRCA, *N*_*tumor*_ = 1097, *N*_*normal*_ *=* 113), cervical and endocervical cancer (CESC, *N*_*tumor*_ = 305, *N*_*normal*_ *=* 3), colon and rectum adenocarcinoma (COADREAD, *N*_*tumor*_ = 383, *N*_*normal*_ *=* 50), glioblastoma multiforme (GBM, *N*_*tumor*_ = 167, *N*_*normal*_ *=* 5), head and neck squamous cell carcinoma (HNSC *N*_*tumor*_ = 521, *N*_*normal*_ *=* 43), kidney chromophobe (KICH, *N*_*tumor*_ = 66, *N*_*normal*_ *=* 25), kidney clear cell carcinoma (KIRC, *N*_*tumor*_ = 530, *N*_*normal*_ *=* 72), kidney papillary cell carcinoma (KIRP, *N*_*tumor*_ = 291, *N*_*normal*_ *=* 32), liver hepatocellular carcinoma (LIHC, *N*_*tumor*_ = 373, *N*_*normal*_ *=* 50), lung adenocarcinoma (LUAD, *N*_*tumor*_ = 510, *N*_*normal*_ *=* 58), lung squamous cell carcinoma (LUSC, *N*_*tumor*_ = 502, *N*_*normal*_ *=* 51), ovarian serous cystadenocarcinoma (OVCA, *N*_*tumor*_ = 266, *N*_*normal*_ *=* 0), prostate adenocarcinoma (PRAD, *N*_*tumor*_ = 498, *N*_*normal*_ *=* 52), skin cutaneous melanoma (SKCM, *N*_*tumor*_ = 472, *N*_*normal*_ *=* 1), thyroid carcinoma (THCA, *N*_*tumor*_ = 513, *N*_*normal*_ *=* 59), and uterine carcinosarcoma (UCS, *N*_*tumor*_ = 57, *N*_*normal*_ *=* 0).

TCGA ccRCC-specific analyses were performed with the KIRC datasets downloaded from Firebrowse (http://firebrowse.org). RSEM-normalized gene level data and reverse phase protein array (RPPA) data were used for gene and protein expression analyses, respectively. Samples that had RNA-Seq, mutation and clinical data (n = 415) were included in the discovery phase of the immune infiltration clusters.

The Sato et al. [29] Agilent microarray gene expression dataset was downloaded from ArrayExpress (http://www.ebi.ac.uk/arrayexpress/experiments/E-MTAB-1980/) and all samples (n = 101) were included in the analysis. The probe identifiers in the Agilent platform were mapped to HGNC gene symbols and the arithmetic mean across identifiers was used for cases where multiple Agilent identifiers mapped to a single HGNC symbol.

The Gerlinger et al. [57] Affymetrix Human Gene 1.0 ST microarray gene expression dataset was obtained via personal communication with the authors on 10 November 2014. This dataset includes 56 tumor and six normal samples from nine ccRCC patients. All samples were included in our analysis. The probe sets in this Affymetrix platform were mapped to HGNC gene symbols and the geometric mean across probe sets was used for cases where multiple probe sets mapped to a single HGNC symbol.

#### Nivolumab-treated patients

Pre-treatment biopsies of six metastatic ccRCC patients were obtained and RNA-Seq datasets were generated. Reads were aligned with TopHat [68]. Gene quantification was performed with RNA-SeQC [69]. Stratification of the patients was based on objective response to nivolumab by RECIST criteria.

#### TCGA mutation data

PANCAN mutation calls were downloaded from the BROAD Firehose’s stddata_2015_02_04 dataset (http://gdac.broadinstitute.org/). Additional COADREAD mutation calls were obtained from the MSKCC cBio portal [70] via personal communication. These mutation calls were used for all analyses, excluding neo-antigen analysis.

#### Clinical data

Clinical data for the TCGA dataset were obtained from the supplementary files of the ccRCC marker paper [28] (KIRC + Clinical + Data + Jul-31-2012). Vital status was determined from the field “Composite Vital status.” Clinical data for the SATO dataset were obtained through direct communication with the authors. Purity values for SATO samples were computed based on the levels of chromosome 3p loss.

### Gene signatures

Marker genes for immune cell types were obtained from Bindea et al. [14]. Angiogenesis marker genes were obtained from Masiero et al. [41]. A signature of antigen presentation was created based on genes exclusively involved in processing and presentation of antigens on MHC [12]. All signature genes are listed in Additional file 2: Table S1.

### Implementation of ssGSEA

Infiltration levels for immune cell types and activity levels for angiogenesis and antigen presentation were quantified using the ssGSEA [30] implementation in R package gsva [71]. ssGSEA is a rank-based method that computes an overexpression measure for a gene list of interest relative to all other genes in the genome. Normalized RNA-Seq or microarray datasets mentioned above were provided as input without further processing (i.e. no standardization or log transformation). A typical execution is gsva(data, list_of_signatures, method=”ssgsea”). The output for each signature is a near-Gaussian list of decimals that can be used in visualization/statistical analysis without further processing.

### Aggregate TIS and IIS scores

The ssGSEA scores for each individual immune cell type were standardized across all tumor and normal samples in the investigated 19 tumor types (n = 8200). The TIS was defined as the mean of the standardized values for the following T cell subsets: CD8 T, T helper, T, T central and effector memory, Th1, Th2, Th17, and Treg cells. T gamma delta and T follicular helper cells were excluded from TIS and IIS because public gene expression maps from healthy tissues show that certain genes in the T gamma delta signature (C1orf61, FEZ1) and the T follicular helper signature (B3GAT1, HEY1, CHGB, CDK5R1) are expressed at elevated levels in healthy brain tissue [72], which is consistent with previous studies that reported the expression of some T cell specific genes in healthy brain [31].

The overall immune infiltration score for a sample was similarly defined as the mean of the standardized values for macrophages, DC subsets (total, plasmacytoid, immature, activated), B cells, cytotoxic cells, eosinophils, mast cells, neutrophils, NK cell subsets (total, CD56bright, CD56dim), and all T cell subsets used in the computation of TIS.

### Flow cytometry and RNA-Seq profiling for in vitro validation of gene signatures

We obtained ccRCC patient specimens at MSKCC and sorted tumor-associated macrophages (n = 4), NK CD16^+^ cells (n = 2), CD8^+^ T cells (n = 5), and CD4^+^ T cells (n = 3) using the sorting markers *CD*45^+^ *CD*3^–^ *CD*56^–^ *CD*14^+^, *CD*45^+^ *CD*3^–^ *CD*56^+^ *CD*16^+^, *CD*45^+^ *CD*3^+^ *CD*8^+^, and *CD*45^+^ *CD*3^+^ *CD*4^+^, respectively. *CD*45^–^ non-immune cells were also sorted from one ccRCC specimen. The antibodies used for cell sorting were: CD14 (HCD14; Biolegend #325608), CD8a (HIT8a; Biolegend #300926), CD45 (2D1; eBioscience 11-9459-42), CD4 (SK3; eBioscience 8048-0047-025), CD16 (3G8; Biolegend 302008), CD56 (HCD56; Biolegend 318318), and CD3 (7D6; Invitrogen MHCD0317).

RNA-Seq data for each sample were generated using an Ion Proton system. FASTQ files were mapped to the target genome using the rnaStar aligner [73] that maps reads genomically and resolves reads across splice junctions. We used the two-pass mapping method outlined in Engström et al. [74] in which the reads are mapped twice. The first mapping pass uses a list of known annotated junctions from Ensemble. Novel junctions found in the first pass are then added to the known junctions and a second mapping pass is done. After mapping, we computed the expression count matrix from the mapped reads using HTSeq [75] and one of several possible gene model databases. The raw count matrix generated by HTSeq was then normalized using the R/Bioconductor package DESeq [76].

This dataset is deposited in Gene Expression Omnibus with accession number GSE84697.

### Multiplex immunofluorescence staining and RNA-seq profiling for in vitro validation of immune cell scores

Unstained pathologic slides of 10 renal tumors from previously untreated patients who underwent either radical or partial nephrectomy for sporadic, resectable ccRCC were obtained and reviewed by a genitourinary pathologist. Paraffin-embedded tissue sections were de-waxed with xylene and rehydrated by gradient ethanol solutions. Antigen retrieval was then performed and the sections were subsequently blocked by bovine serum albumin plus serum with the addition of mouse monoclonal anti-human CD8 (Dako, clone C8/144B, catalogue #M7103 [77]), CD56 (Thermo scientific, clone 56C04, catalogue #MS-1149-P1 [78]) and FOXP3 (Abcam, clone 236A/E7, catalogue #ab20034 [79]). The sections were incubated with HRP-conjugated anti-mouse antibodies. TSA plus kits (Perkin Elmer) were used according to the manufacturer’s instructions. Finally, a Leica upright confocal microscope was used to capture images. In order to quantify the degree of cellular infiltration, the individual positive cells for CD56, CD8, and FOXP3 were counted in three representative regions of each tumor. The ratio of CD56, CD8, and FOXP3 positive cells versus total cells (DAPI-stained) were determined.

RNA-Seq was performed for all samples and raw output BAMs were converted back to FASTQ using PICARD Sam2Fastq. Maps were then mapped to the human genome using STAR aligner [73]. The genome used was HG19 with junctions from ENSEMBL (GRCh37.69_ENSEMBL) and a read overhang of 49. Then any unmapped reads were mapped to HG19 using BWA MEM (version 0.7.5a). The two mapped BAMs were then merged and sorted and gene level counts were computed using htseq-count (options -s y -m intersection-strict) and the same gene models as used in the mapping step [75]. This dataset was previously deposited in Gene Expression Omnibus with accession number GSE74734 [80].

### In silico validation of the ssGSEA immune cell scoring methodology using simulated mixing proportions

In order to robustly validate the ability of ssGSEA to quantify infiltrating immune cells from whole tumor RNA-Seq, we generated realistic in silico mixtures of tumor and infiltrating cell RNA expression. These mixtures emulate the gene expression profile obtained from bulk RNA-Seq of impure tumor specimens. The steps of this validation consisted of: (1) generating reference mRNA expression vectors for tumor-infiltrating immune cell populations; (2) creating noiseless or noisy linear combinations of these “pure” expression vectors using known mixing proportions; (3) running the ssGSEA method on in silico mixtures to obtain the inferred immune cell levels; (4) computing, for each cell type and at each noise level, the Spearman correlation (point estimate) between the known mixing proportions and the inferred levels; and (5) generating an empirical null distribution for the Spearman correlations to obtain bootstrap *p* values associated with the point estimates. We elaborate on the details of these steps below.

1. Generating reference mRNA expression vectors for tumor-infiltrating immune cells
  Few expression profiles of tumor-infiltrating immune cell populations exist in the literature. Thus, we utilized the four key immune cell populations and one non-immune cell population (*CD*45^–^) we sorted from ccRCC tumor specimens, performed RNA-Seq, and generated novel reference mRNA expression vectors defined as the mean of the RNA-Seq readout for each gene across the samples (4 macrophage, 2 NK CD16^+^, 5 CD8^+^ T, 3 CD4^+^ T samples, and 1 *CD*45^–^ non-immune sample).
2. Generating in silico mixtures with simulated mixing proportions:
  The “clean” dataset
    The cell types that we have a reference gene expression vector for are macrophages, NK cells, *CD*8^+^, and *CD*4^+^ T cells, and the non-immune *CD*45^–^ cells. An in silico mixture that would realistically simulate the gene expression profile of the tumor microenvironment can be obtained by linearly combining the immune cell reference expression vectors with that of non-immune *CD*45^–^ cells. We created 200 such in silico mixture samples by randomly generating mixing proportions from a Uniform(0,1) distribution (point (5) below) and then computing linear combinations of 20,032 genes in the reference expression vectors of the five cell types. This dataset of 200 in silico samples and 20,032 genes constitutes the noiseless dataset that will be referred to as the “clean” dataset from here on.
  The “noisy” datasets
    Since the RNA-Seq readout from a tumor specimen may include both biological and technical noise, we tested the performance of our decomposition pipeline in “noisy” datasets as well as in the “clean” dataset. We tested 10 different noise levels ranging from a slightly noisy SNR of 10:1 to an extremely noisy setting of SNR 1:1. For an **S**:1 noise level, we added Gaussian noise to each gene in a “clean” sample with mean 0 and standard deviation equal to the mRNA readout of the gene divided by **S**. Each one of the 10 noisy datasets again has 200 samples and 20,032 genes.
3. Measuring the performance of the ssGSEA methodology:
  We implemented our ssGSEA decomposition pipeline on both the “clean” and the noisy datasets with the signatures for “macrophages,” “NK cells,” “CD*8* ^*+*^ T cells,” and “T helper cells.” The Bindea et al. [14] signature set did not have a signature for *CD*4^+^ T cells, but had an umbrella signature for T helper cells that would be valid for all *CD*4^+^ T cells. We then computed the Spearman correlation between the inferred levels (ssGSEA scores) of these cell types in the 200 samples and the known mixing proportions from the simulations. Note that the decomposition on even the “clean” dataset has an “impurity” component as the expression from *CD*45^–^ cells is also integrated into the mixture samples. The Spearman correlations were stable and above 0.6 for all four cell types in a long SNR range from 9:1 to 4:1 (Fig. 2a).
  Comparing the four cell types, the correlation values are the highest for NK cells (greater than 0.8 until SNR 4:1) and the lowest for *CD*4^+^ T cells. The high number of polarization and activation states in the sorted *CD*4^+^ T cells might be creating challenges against obtaining a *CD*4^+^ reference expression profile that will universally be highly robust. However, the deficiency in performance is only in relation to the other three cell types; the bootstrap *p* values for the *CD*4^+^ T cell Spearman correlations are statistically significant (*α* = 0.05) as explained below.
4. Obtaining bootstrap *p* values for the observed Spearman correlations:
  Even though the point estimates for the Spearman correlations as computed in point (2) above remain high in noisy settings, this does not provide information regarding the significance of the point estimates. To this end, we simulated an empirical null distribution for these correlation values by generating 1000 random gene signatures for each one of the four cell types (macrophages, NK cells, CD8^+^ T cells, and T helper cells) for a total of 4000 random signatures. The number of genes in each random signature was equal to the number of genes in the corresponding “real” signature. Thus, each random signature for macrophages, NK cells, CD8^+^ T cells, and T helper cells, respectively, contained 33, 35, 37, and 24 genes randomly chosen from the 20,032 genes in the RNA-Seq dataset.
  We next ran ssGSEA on both the “clean” and the noisy datasets 1000 times, where each run was performed with a different set of random signatures for the four cell types. Thus, each run yielded 200 inferred values for a particular cell type, which were then used to compute the Spearman correlation with the true mixing proportions. The 1000 Spearman correlations obtained in this way formed the empirical null distribution for that cell type. The *p* value for each observed Spearman correlation was computed as the fraction of correlations from random signatures that were as large as or larger than the observed correlation (Additional file 1: Figure S23).
5. The algorithm for generating mixing proportions:
  Objective: Simulate five random numbers that follow the Uniform(0,1) distribution and sum to 1.
  Note: If *X ~ U*(0,1), then *PDF*(*x*) = 1 and *CDF*(*x*) = *x*
  Step 1: Generate four random numbers from *CDF*(*x*), i.e. *U*(0,1).
  Step 2: Sort the four random numbers in ascending fashion
  Step 3: Compute the differences between consecutive numbers (three difference values for four random numbers)
  Return: The smallest random number is the first mixing proportion. The differences between consecutive random numbers form mixing proportions 2, 3, and 4. The last mixing proportion is the difference between 1 and the largest random number.

### Orthogonal validation of IIS with methylation-based leukocyte fractions

We estimated the fraction of leukocytes using the assumption that the beta value of a tumor sample i in a DNA methylation probe k is a weighted arithmetic mean of representative values from (1) leukocytes and (2) cancer cells. To make the estimation more robust, we accounted only for those probes (the leukocyte methylation signature) where the leukocyte and tumor methylation difference was extreme. We used a similar approach as described in Carter et al. [36]. All probes were ranked by the difference between mean beta values in leukocyte and tumor samples. The leukocyte methylation signature consisted of the top 1000 probes L_h_ (leukocyte high methylated probes) and the bottom 1000 probes L_l_ (leukocyte low methylated probes).

Let T_ik_ denote the beta value for a probe k in a tumor sample i. Let B_k_ be a representative value of leukocyte methylation and equal the average beta value of leukocyte samples for each probe. Let T_k_ be a representative value of tumor methylation and equal the minimum observed beta value across all tumor samples for the L_h_ probes and the maximum for the L_l_ probes. Thus T_k_ represents the methylation level of the theoretically purest tumor sample. Then, the fraction f_*ik*_ of the leukocyte component for sample i and probe k is given by the following: T_ik_ = B_k_f_*ik*_ + T_k_(1 - f_*ik*_), hence f_*ik*_ = (T_ik_ - T_k_)/(B_k_ - T_k_). The leukocyte fraction f_*i*_ for a sample is then calculated as the mode (e.g. argmax of the density) of the estimated distribution of all f_*ik*_ for the leukocyte methylation signature. The reference DNA methylation levels for leukocytes were derived by Reinius et al. [81] from the DNA methylation profile of peripheral blood mononuclear cells (PBMCs) in six healthy donors.

### Principal component test for Bindea et al. signatures

We performed an internal test for the immune cell gene signatures on the three HG-U133A microarray datasets [31–33] originally used by Bindea et al. [14] to derive the signatures. The combined dataset had a total of 46 samples from 14 unique immune cell types. We first performed background correction and quantile normalization on the CEL files using GCRMA [34]. We then performed two consecutive PCAs to investigate the separation of (1) all 14 immune cell types, and (2) only the T cell subpopulations among the set of 14 cell types.

1. PC separation of all immune cell types: we reduced the GCRMA-normalized dataset to the signature genes by mapping the Affymetrix U133A probeset identifiers to HGNC symbols with the R biomaRt package [82] and filtering out the zero variance probesets. A total of 840 probesets remained, corresponding to the 501 unique genes used in the immune cell signatures. A PCA on the normalized and reduced dataset revealed batch effects from the three data sources (Additional file 1: Figure S3, top panel). We corrected for batch effects using the non-parametric option in ComBat [35] (Additional file 1: Figure S3, bottom panel) and subsequently performed PCA on the 46 samples to investigate the separation of immune cell types by the first two PCs (Fig. 1a).
2. PC separation of six T cell subpopulations: we reduced the GCRMA-normalized dataset to the 19 T cell subpopulation samples and only the T cell related signature genes in a similar manner to point (1). A total of 400 probesets remained, corresponding to the 225 unique T cell subpopulation signature genes. Batch effects were corrected using the non-parametric option in ComBat [35] and PCA was subsequently performed on the 19 samples to investigate the separation of T cell subpopulations (Additional file 1: Figure S5).

### Comparison between CIBERSORT and ssGSEA immune scores

We obtained CIBERSORT values for the TCGA KIRC cohort using the web tool https://cibersort.stanford.edu/ on 26 August 2016. The RNA-Seq dataset was provided as input and the algorithm was run with 1000 permutations (the highest option available). The quantile normalization (QN) option was disabled as the RSEM pipeline for TCGA RNA-Seq datasets included QN. Samples with a global *p* value > 0.05 were removed and the remaining 194 samples were used in the comparison with ssGSEA. We calculated the Pearson correlation between Bindea et al. signatures and CIBERSORT values for all relevant cell types (Additional file 2: Table S9).

### Clonality assessment

The number of subclones for TCGA and SATO ccRCC samples was calculated using the R package SciClone (version 1.0.7) [60] with default parameters. For SATO samples, the depth of coverage was assumed to be at least 100×. Three of the SATO samples had an insufficient number of copy-number neutral variants.

### HLA typing and HLA-binding neoepitope prediction

Whole-exome sequences for the TCGA KIRC tumors were downloaded using cgquery (https://gdc.cancer.gov/). Whole-exome sequences for the SATO dataset were downloaded from the European Genome-phenome Archive (https://www.ebi.ac.uk/ega/studies/EGAS00001000509). BAM files containing whole-exome sequences from normal and/or tumor samples were processed to obtain fastq files. Reads that aligned to HLA-A, HLA-B, or HLA-C genes using RazerS3 [83] (http://www.seqan.de/projects/razers/) were passed as input to OptiType v1.0 [62] (https://github.com/FRED-2/OptiType). Discrepancies in HLA typing were resolved by consensus or exclusion. A MAF files containing missense mutations for each TCGA patient was obtained from cBioPortal (http://www.cbioportal.org/). A MAF file containing missense mutations for each SATO patient was obtained from the publication [29]. Samtools (v 0.1.19) and snpEff (v3. 5C) were used to identify the protein context surrounding each missense mutation from a canonical set of human transcripts in (Hg GRCh37.74). All 9 and 10-mers overlapping the missense mutations were extracted and NetMHCPan [84] was used to predict their affinity to alleles of MHC-I.

### Statistical methods

#### Hypothesis tests

Two-sided Mann–Whitney and Fisher’s exact tests were performed with the R functions wilcox.test and fisher.test, respectively. These tests are appropriate as they are non-parametric (distribution-free). One-way ANOVA tests were performed with the R function aov for purity, stromal infiltration, and immune infiltration scores. This test is appropriate as the variance of the scores is similar between the immune infiltration clusters and ssGSEA scores from gsva [71] are approximately normal. *P* values were adjusted for multiple hypothesis testing using the R function p.adjust with the “fdr” option.

#### Unsupervised clustering

The unsupervised clustering for tumor samples, immune cell types, genes, and proteins was performed with hierarchical clustering, Ward linkage, and Euclidean distance.

#### Random forest prediction of immune infiltration class for SATO patients

A random forest classifier was trained on the TCGA cohort of 415 patients with 10,000 trees and otherwise default values in the R package randomForest [85]. Training error on the TCGA cohort was 0%. This classifier was applied to the ssGSEA scores of the SATO and GERLINGER cohorts to obtain class predictions. The random forest R object and the code to predict the class of a new sample are available upon request.

#### Survival analysis

*P* values in Fig. 6b were obtained from univariate Cox proportional-hazards regression models using the R package survival. Chi-square test statistics in Kaplan–Meier curves (Fig. 6a, c, Additional file 1: Figure S15c) were computed using log-rank tests.

#### Ratio of cell counts

ssGSEA-based infiltration scores do not follow a discrete count distribution, but are unimodal and approximately normal [71]. Therefore, ratios of cell counts cannot be determined by simple division of the ssGSEA scores. However, if *a* and *b* represent two cell counts, the log of the ratio *a*/*b* is equal to log(*a*) – log(*b*). Thus, the difference of two ssGSEA scores represents a ratio of cell counts. The CD8^+^ T/Treg and Th17/Th2 ratios in Fig. 6b and c denote the numeric difference between the ssGSEA scores for these cell types.

## Additional files

Additional file 1: Figures S1–S25. Supplementary figures. (PDF 15387 kb)
Additional file 2: Tables S1–S9. Supplementary tables. (XLSX 3868 kb)
