# Erratum to: Tumor immune microenvironment characterization in clear cell renal cell carcinoma identifies prognostic and immunotherapeutically relevant messenger RNA signatures

**DOI:** 10.1186/s13059-017-1180-8

**Published:** 2017-03-01

**Authors:** Yasin Şenbabaoğlu, Ron S. Gejman, Andrew G. Winer, Ming Liu, Eliezer M. Van Allen, Guillermo de Velasco, Diana Miao, Irina Ostrovnaya, Esther Drill, Augustin Luna, Nils Weinhold, William Lee, Brandon J. Manley, Danny N. Khalil, Samuel D. Kaffenberger, Yingbei Chen, Ludmila Danilova, Martin H. Voss, Jonathan A. Coleman, Paul Russo, Victor E. Reuter, Timothy A. Chan, Emily H. Cheng, David A. Scheinberg, Ming O. Li, Toni K. Choueiri, James J. Hsieh, Chris Sander, A. Ari Hakimi

**Affiliations:** 1 Computational Biology Center, Memorial Sloan Kettering Cancer Center, New York, NY USA; 2 Molecular Pharmacology and Chemistry Program, Memorial Sloan Kettering Cancer Center, New York, NY USA; 3 Urology Service, Department of Surgery, Memorial Sloan Kettering Cancer Center, New York, NY USA; 4 Immunology Program, Memorial Sloan Kettering Cancer Center, New York, NY USA; 5 Department of Medical Oncology, Dana-Farber Cancer Institute, Boston, MA USA; 6 Department of Epidemiology and Biostatistics, Memorial Sloan Kettering Cancer Center, New York, NY USA; 7 Department of Radiation Oncology, Memorial Sloan Kettering Cancer Center, New York, NY USA; 8 Department of Pathology, Memorial Sloan Kettering Cancer Center, New York, NY USA; 9 Sidney Kimmel Comprehensive Cancer Center, Johns Hopkins University School of Medicine, Baltimore, MD USA; 10 Vavilov Institute of General Genetics, Russian Academy of Sciences, Moscow, Russia; 11 Genitourinary Oncology Service, Department of Medicine, Memorial Sloan Kettering Cancer Center, New York, NY USA; 12 Human Oncology & Pathogenesis Program, Memorial Sloan Kettering Cancer Center, New York, NY USA; 13 Department of Medicine, Memorial Sloan Kettering Cancer Center, New York, NY USA; 14 Weill Cornell Medical College, New York, NY USA; 15 Present address: Swim Across America/Ludwig Collaborative Laboratory, Department of Medicine, Memorial Sloan Kettering Cancer Center, New York, NY USA

## Erratum

After publication of this article [1] we noticed that authors Yasin Şenbabaoğlu and Ron S. Gejman were omitted as equal contributors. This has now been corrected in the author list.

The publisher apologises for these errors.
