# Supplementary material for: Tumor immune microenvironment characterization in clear cell renal cell carcinoma identifies prognostic and immunotherapeutically relevant messenger RNA signatures
Source: Genome Biol. 2016 Nov 17;17:231. doi: 10.1186/s13059-016-1092-z (PMC5114739; doi:10.1186/s13059-016-1092-z)
Supplement: Additional file 1: Figures S1–S25. — Supplementary figures. (PDF 15387 kb) [file 13059_2016_1092_MOESM1_ESM.pdf]

Supplementary Figure 1

a. Workflow for tumor-immune infiltrate profiling

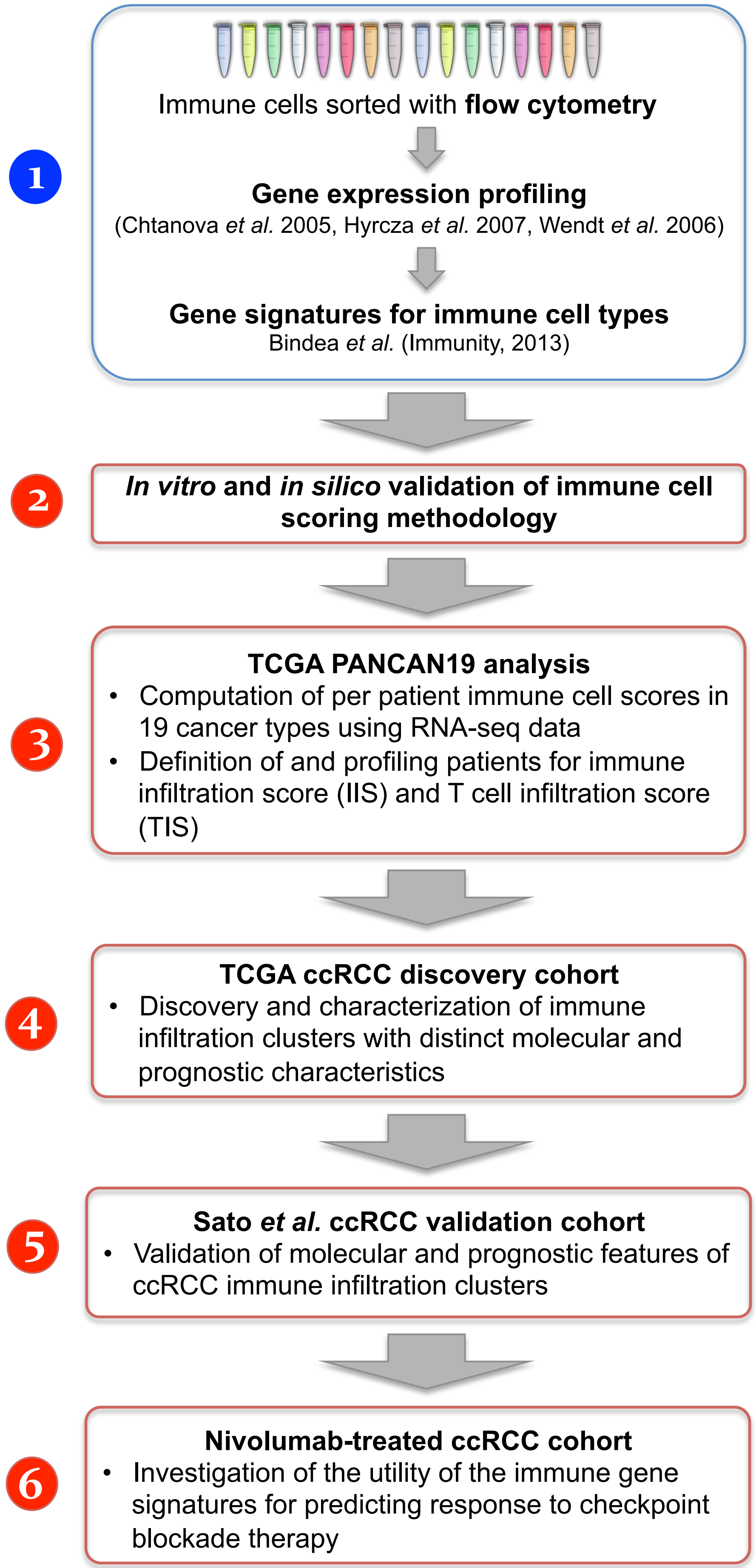

b. Investigated immune cell types and gene signatures

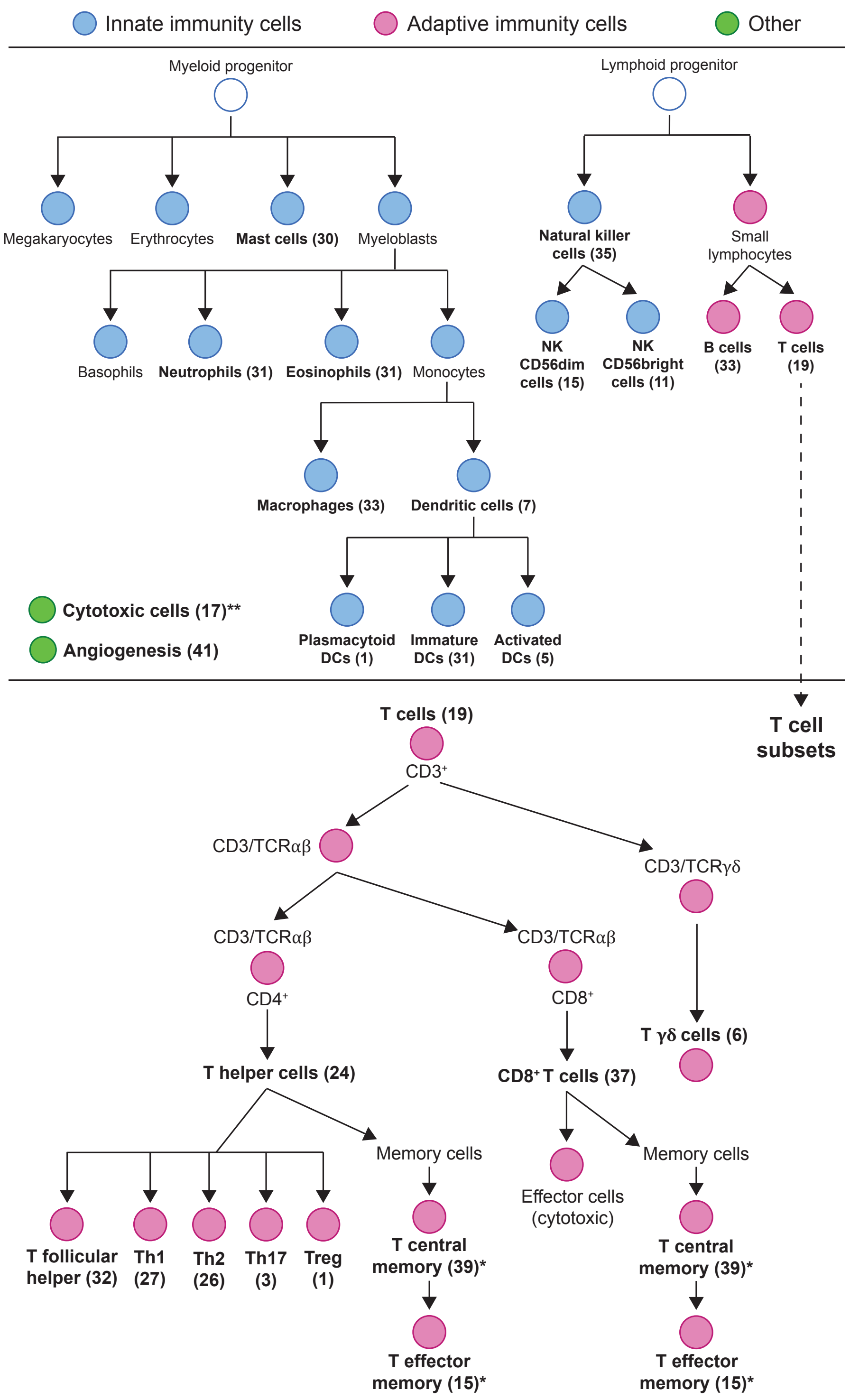

* T central and effector memory cells were not gated for CD4 or CD8.
** The cytotoxic cell signature includes genes overexpressed in CD8+ T cells, T γδ cells, and NK cells.

Figure S1. Workflow and the investigated immune cell types. (a) Workflow for tumor immune-infiltrate profiling. **(b)** The investigated immune cell types are shown (**bold**) with two hierarchical trees: innate and adaptive immunity cell types (top panel), and distinct T cell subsets (bottom panel). The number of genes in each signature is displayed in parentheses next to the studied cell type.

Supplementary Figure 2

	aDC (5)	Angiogenesis (41)	APM genes (7)	B cells (33)	CD8 T cells (37)	CTLA-4	Cytotoxic cells (17)	Dendritic cells (7)	Eosinophils (31)	Immature DCs (31)	Macrophages (33)	Mast cells (30)	Neutrophils (31)	NK CD56bright cells (11)	NK CD56dim cells (15)	NK cells (35)	PD-1	Plasmacytoid DCs (1)	PD-L1	T cells (19)	T helper cells (24)	Tcm cells (39)	Tem cells (15)	Tfh cells (32)	Tgd cells (6)	Th1 cells (27)	Th17 cells (3)	Th2 cells (26)	Treg cells (1)
aDC		0	0	0	0	0	0	0	0	0	0	0	0	0	0	0	0	0	0	0	0	0	0	0	0	0	0	0	0
Angiogenesis			0	0	0	0	0	0	0	0	0	0	0	0	0	0	0	0	0	0	0	0	0	0	0	0	0	0	0
APM genes				0	0	0	0	0	0	0	0	0	0	0	0	0	0	0	0	0	0	0	0	0	0	0	0	0	0
B cells					0	0	0	0	0	0	0	0	0	0	0	0	0	0	0	0	0	0	0	0	0	0	0	0	0
CD8 T cells						0	1	0	0	0	0	0	0	0	0	0	0	0	0	0	0	0	0	0	0	0	0	0	0
CTLA-4							0	0	0	0	0	0	0	0	0	0	0	0	0	0	0	0	0	0	0	1	0	0	0
Cytotoxic cells								0	0	0	0	0	0	0	0	0	0	0	0	0	0	0	0	0	0	0	0	0	0
Dendritic cells									0	0	0	0	0	0	0	0	0	0	0	0	0	0	0	0	0	0	0	0	0
Eosinophils										0	0	0	0	0	0	0	0	0	0	0	0	0	0	0	0	0	0	0	0
Immature DCs											0	0	0	0	0	0	0	0	0	0	0	0	0	0	0	0	0	0	0
Macrophages												0	0	0	0	1	0	0	0	0	0	0	0	0	0	0	0	0	0
Mast cells													0	0	0	0	0	0	0	0	0	0	0	0	0	0	0	0	0
Neutrophils														0	0	0	0	0	0	0	0	0	0	0	0	0	0	0	0
NK CD56bright cells															0	1	0	0	0	0	0	0	0	0	0	0	0	0	0
NK CD56dim cells																0	0	0	0	0	0	0	0	0	0	0	0	0	0
NK cells																	0	0	0	0	0	0	0	0	0	1	0	0	0
PD-1																		0	0	0	0	0	0	1	0	0	0	0	0
Plasmacytoid DCs																			0	0	0	0	0	0	0	0	0	0	0
PD-L1																				0	0	0	0	0	0	0	0	0	0
T cells																					3	0	1	0	0	0	0	0	0
T helper cells																						0	1	0	0	0	0	0	0
Tcm cells																							0	0	0	0	0	0	0
Tem cells																								0	0	0	0	0	0
Tfh cells																									0	0	0	0	0
Tgd cells																										0	0	0	0
Th1 cells																											0	0	0
Th17 cells																												0	0
Th2 cells																													0
Treg cells																													

Figure S2. Number of genes shared between signatures. The number in each box denotes the number of genes in common between the corresponding signatures. 98.4% (501) of these genes were used uniquely in only one signature

Supplementary Figure 3

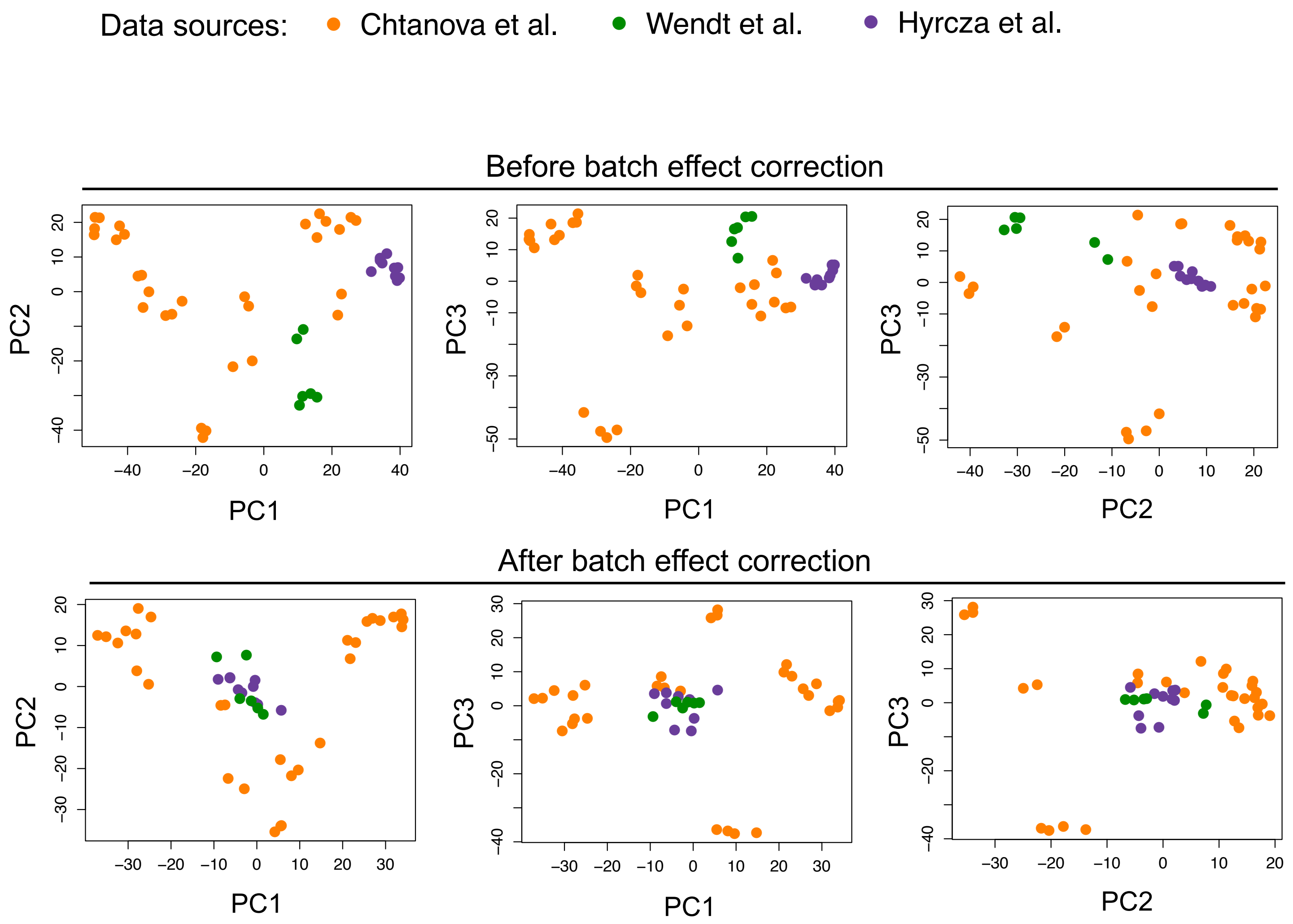

Figure S3. Batch effect correction for the three microarray datasets used to derive immune cell gene signatures. PC analysis on the GCRMA-normalized microarray expression data using 501 signature genes revealed batch effects from the three data sources (top panel). Batch effects were corrected using the nonparametric option in ComBat (bottom panel). After batch-effect correction, cell types of similar lineages but from different data sources clustered together. Cell type labels are given in the batch-effect-corrected PC plot in Figure 1a.

Supplementary Figure 4

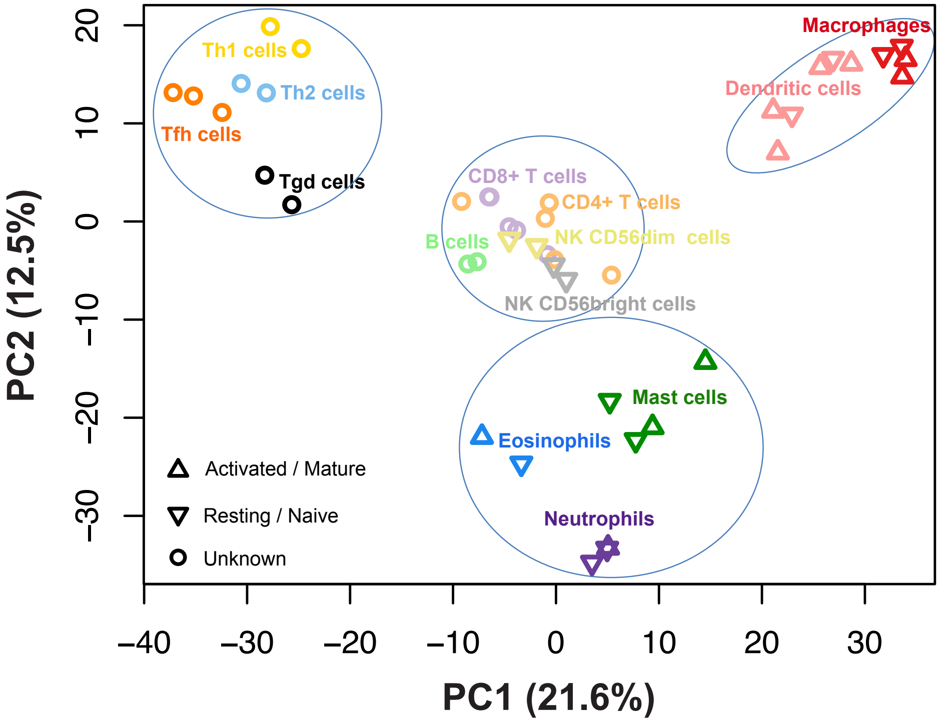

Figure S4. PC separation of 14 immune cell types in training data using transcript levels of signature genes. Microarray gene expression data were generated from immune cell types sorted by magnetic or fluorescent activated cell sorting[31-33] and used in Bindea et al to derive the signatures. Other cell types in Bindea et al., such as the ones for which signature genes are based on biological knowledge (such as Tregs and Th17 cells) or the ones that are umbrella terms (such as T helper cells and cytotoxic cells) could not be included in the PC analysis due to absence of microarray data.

Supplementary Figure 5

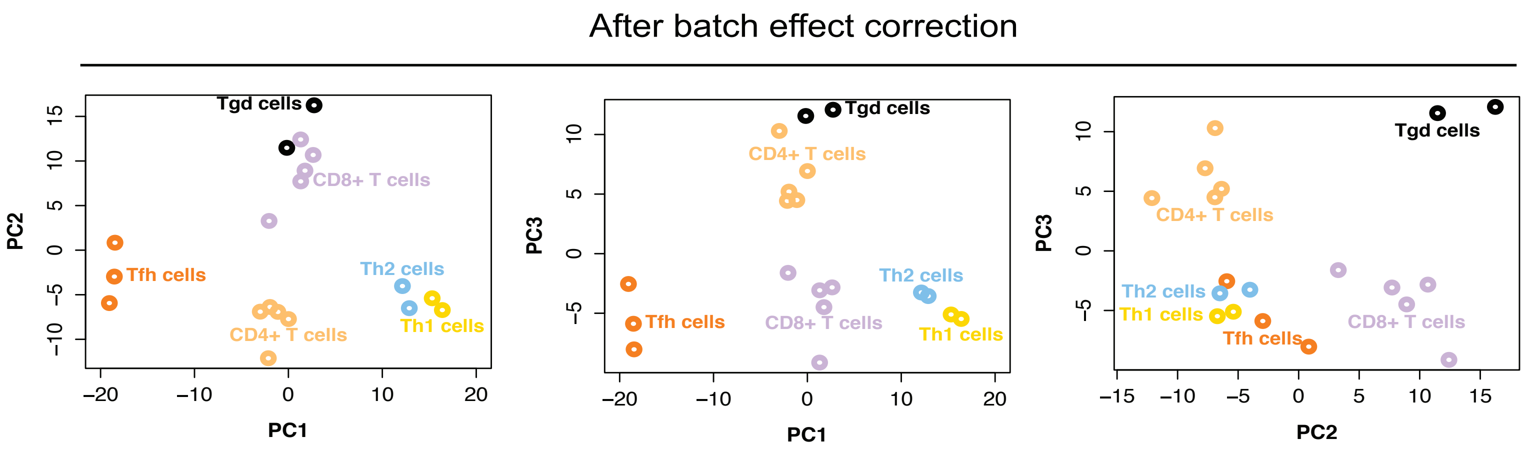

Figure S5. PC separation of T cell subpopulations using signature genes. Microarray gene expression data generated from sorted immune cell types were normalized with GCRMA and filtered to keep only T cell subpopulation samples and signature genes for these subpopulations. Batch effect correction was then performed with ComBat before running PC analysis on the samples. Signature genes achieve a robust separation of T cell subpopulations.

Supplementary Figure 6

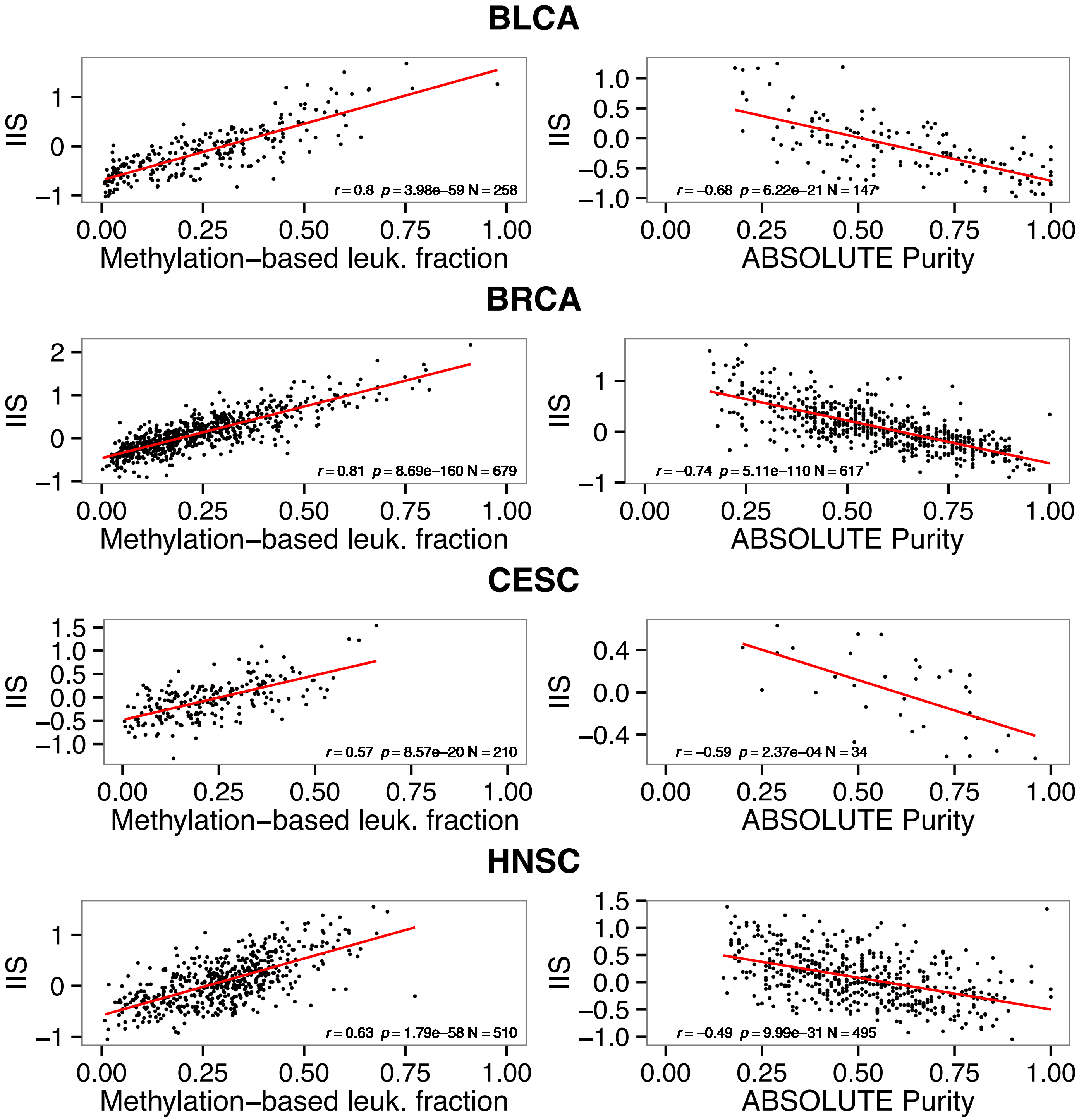

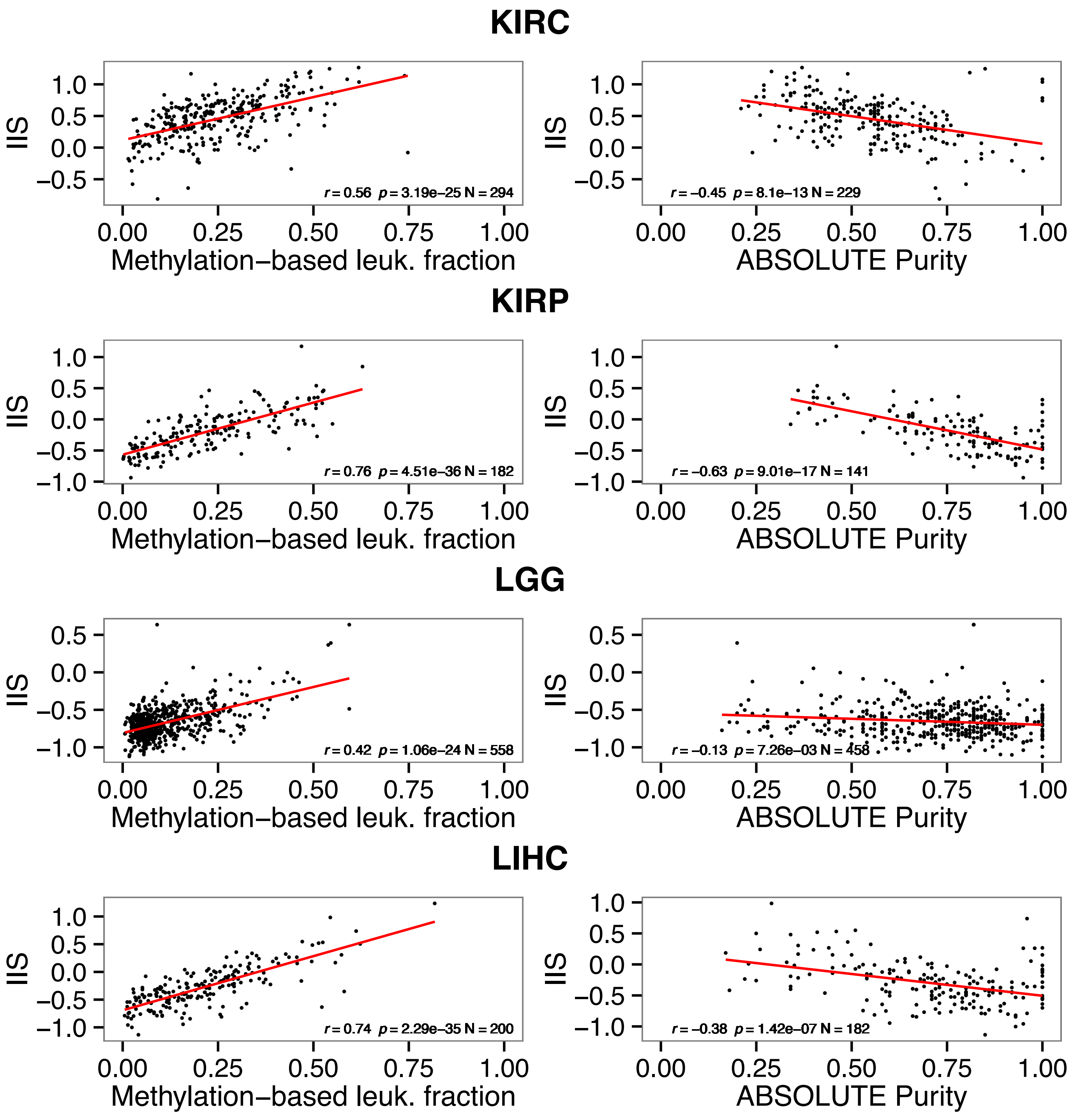
KIRC
r = 0.56 p = 3.19e−25 N = 294
r = −0.45 p = 8.1e−13 N = 229
KIRP
r = 0.76 p = 4.51e−36 N = 182
r = −0.63 p = 9.01e−17 N = 141
LGG
r = 0.42 p = 1.06e−24 N = 558
r = −0.13 p = 7.26e−03 N = 458
LIHC
r = 0.74 p = 2.29e−35 N = 200
r = −0.38 p = 1.42e−07 N = 182
IIS
Methylation−based leuk. fraction
ABSOLUTE Purity
0.00
0.25
0.50
0.75
1.00

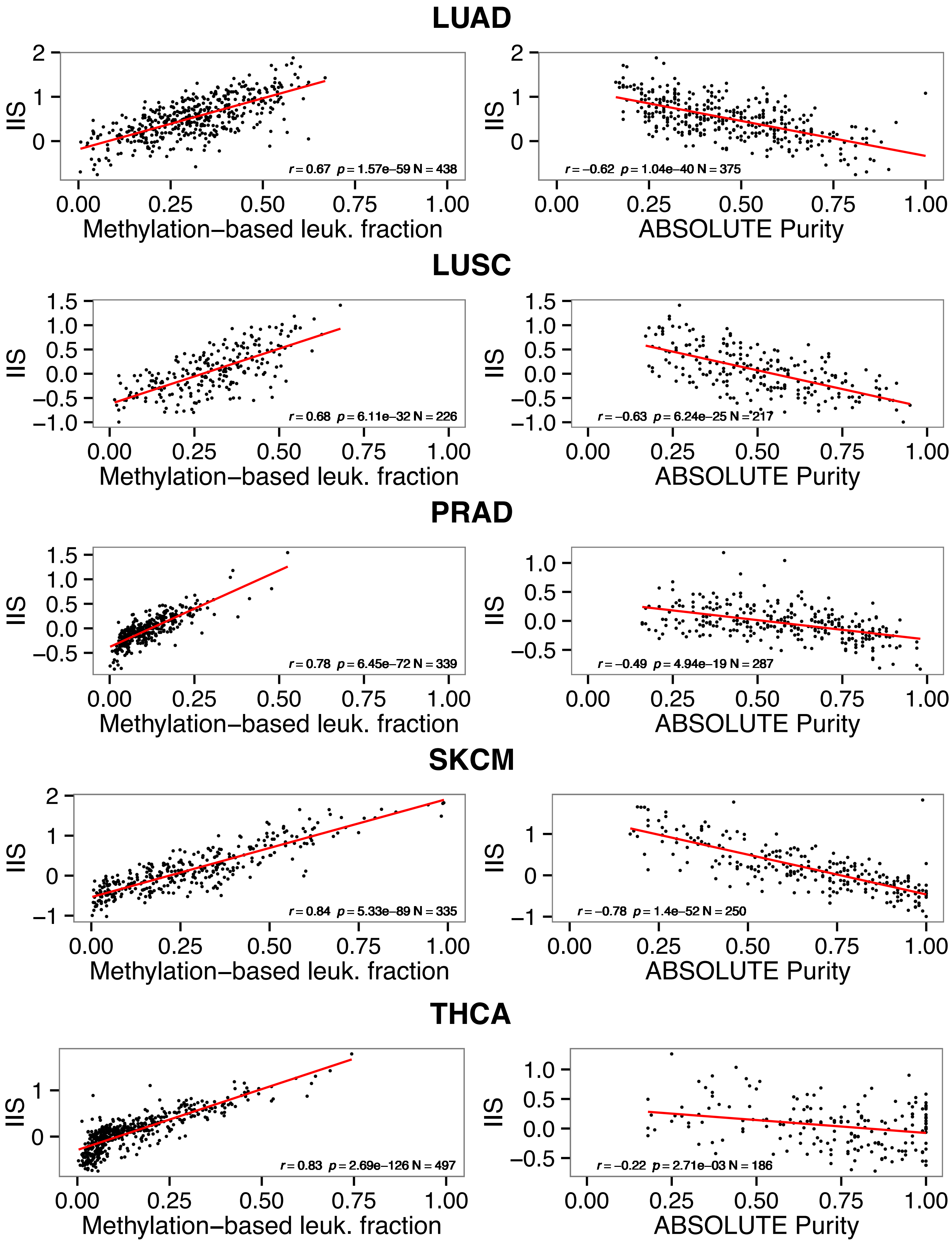
LUAD
IIS
Methylation−based leuk. fraction
r = 0.67 p = 1.57e−59 N = 438
ABSOLUTE Purity
r = −0.62 p = 1.04e−40 N = 375
LUSC
r = 0.68 p = 6.11e−32 N = 226
r = −0.63 p = 6.24e−25 N = 217
PRAD
r = 0.78 p = 6.45e−72 N = 339
r = −0.49 p = 4.94e−19 N = 287
SKCM
r = 0.84 p = 5.33e−89 N = 335
r = −0.78 p = 1.4e−52 N = 250
THCA
r = 0.83 p = 2.69e−126 N = 497
r = −0.22 p = 2.71e−03 N = 186

Figure S6. Overall immune infiltration score (IIS) correlations with methylation-based leukocyte fraction and tumor purity. (Left) The scatter plots of IIS vs. methylation-based leukocyte fraction for 13 tumor types, and (Right) the scatter plots of IIS vs. tumor purity estimates from the ABSOLUTE algorithm. Spearman correlations and the corresponding p-values are shown in each plot. The red lines indicate the regression line for y~x.

Supplementary Figure 7

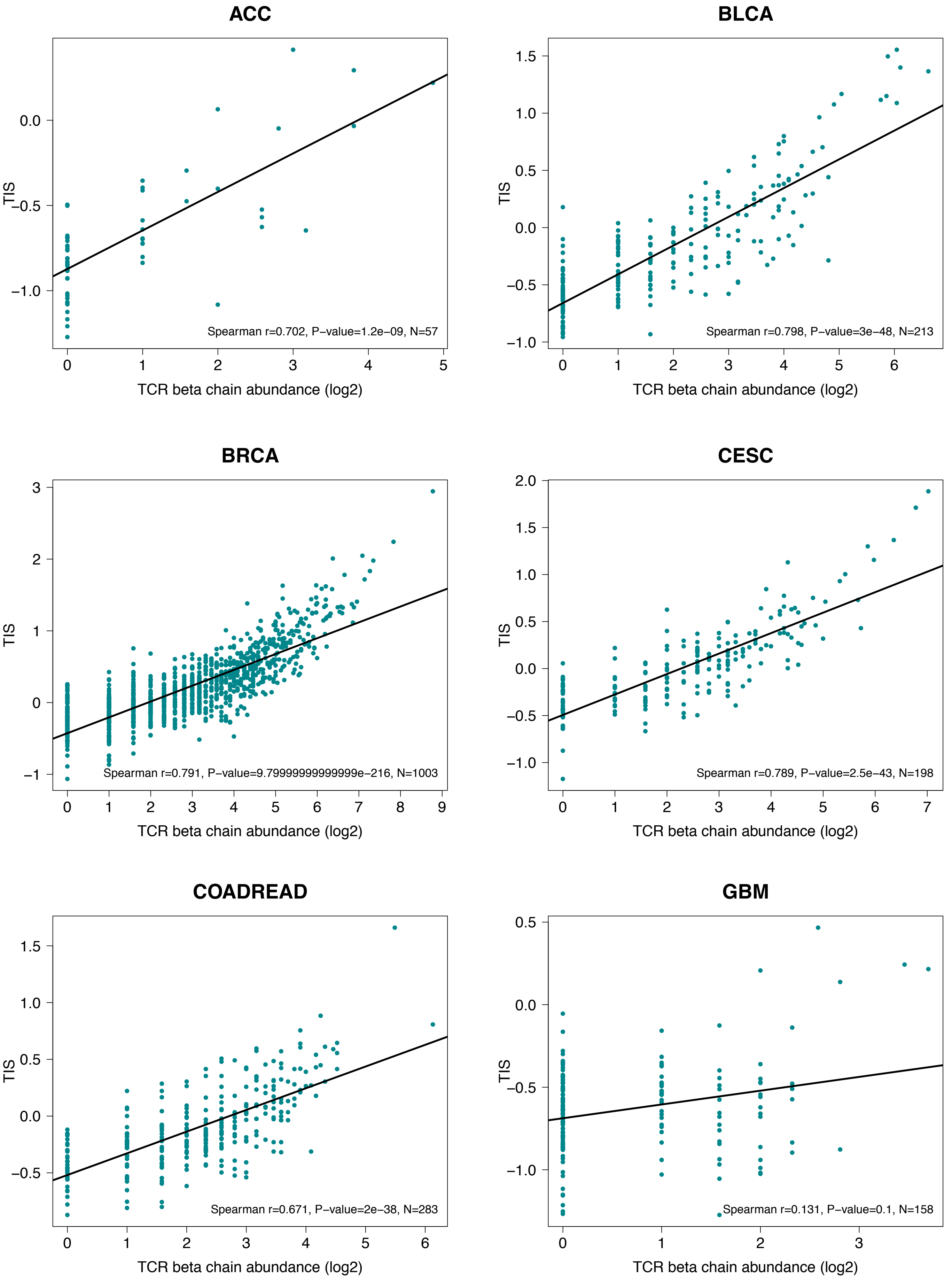

Supplementary Figure 5 (continued)

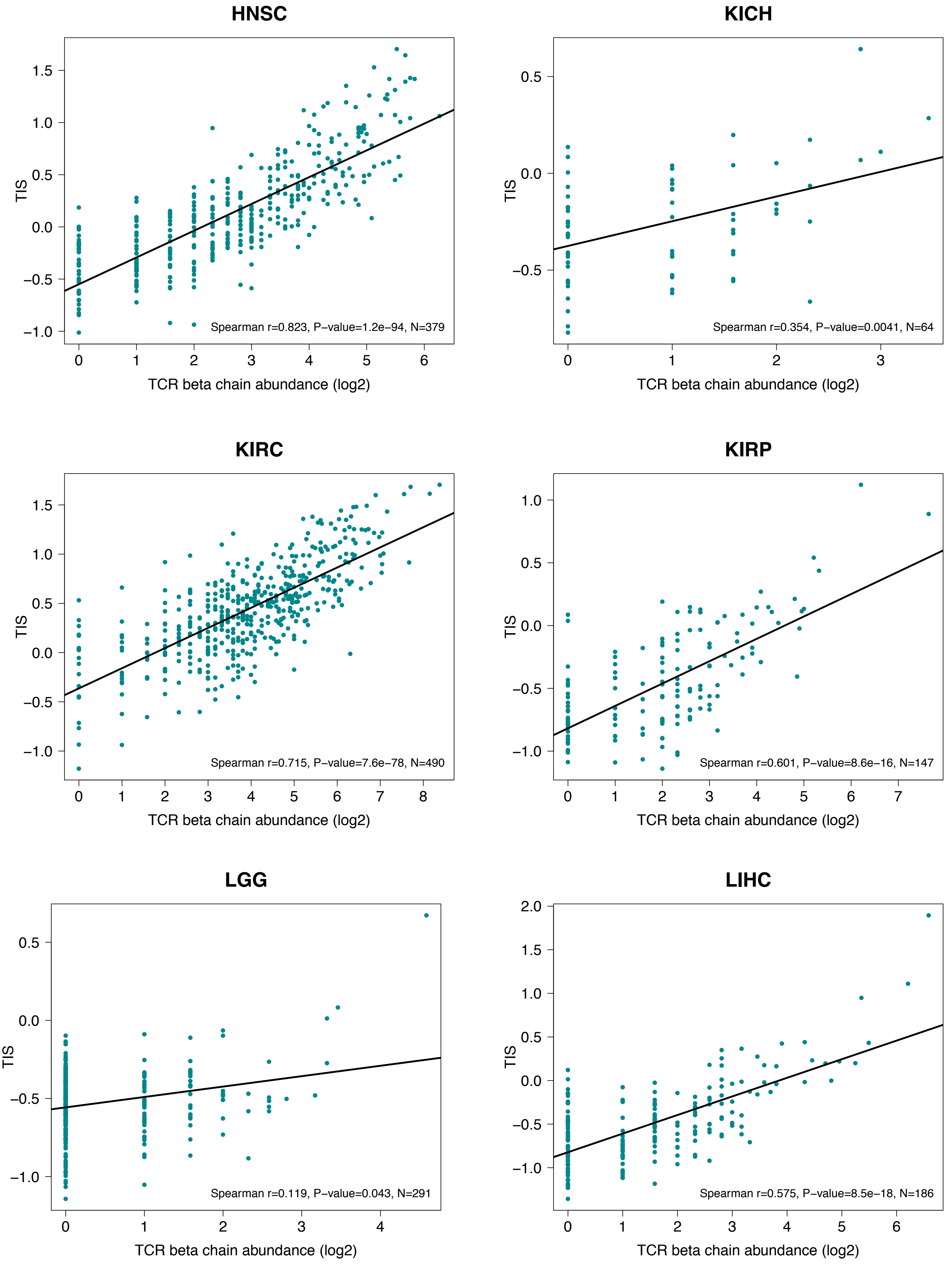

Supplementary Figure 5 (continued)

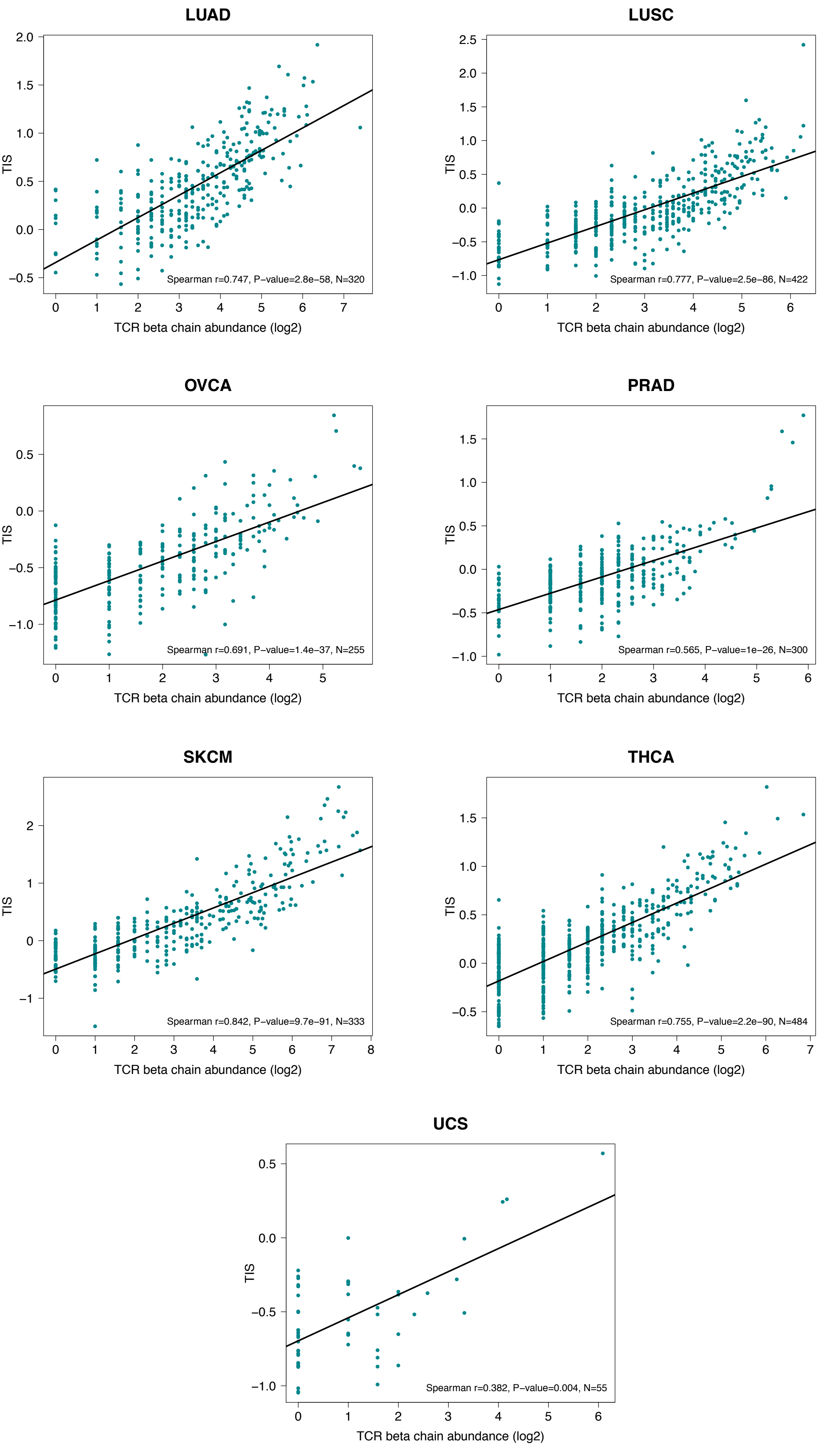

Figure S7. T cell infiltration score (TIS) correlations with TCR beta chain abundance. The scatter plots of TIS vs. TCR beta chain abundane for 19 tumor types. Spearman correlations and the corresponding p-values are shown in each plot. The black lines indicate the regression line for y~x.

Supplementary Figure 8

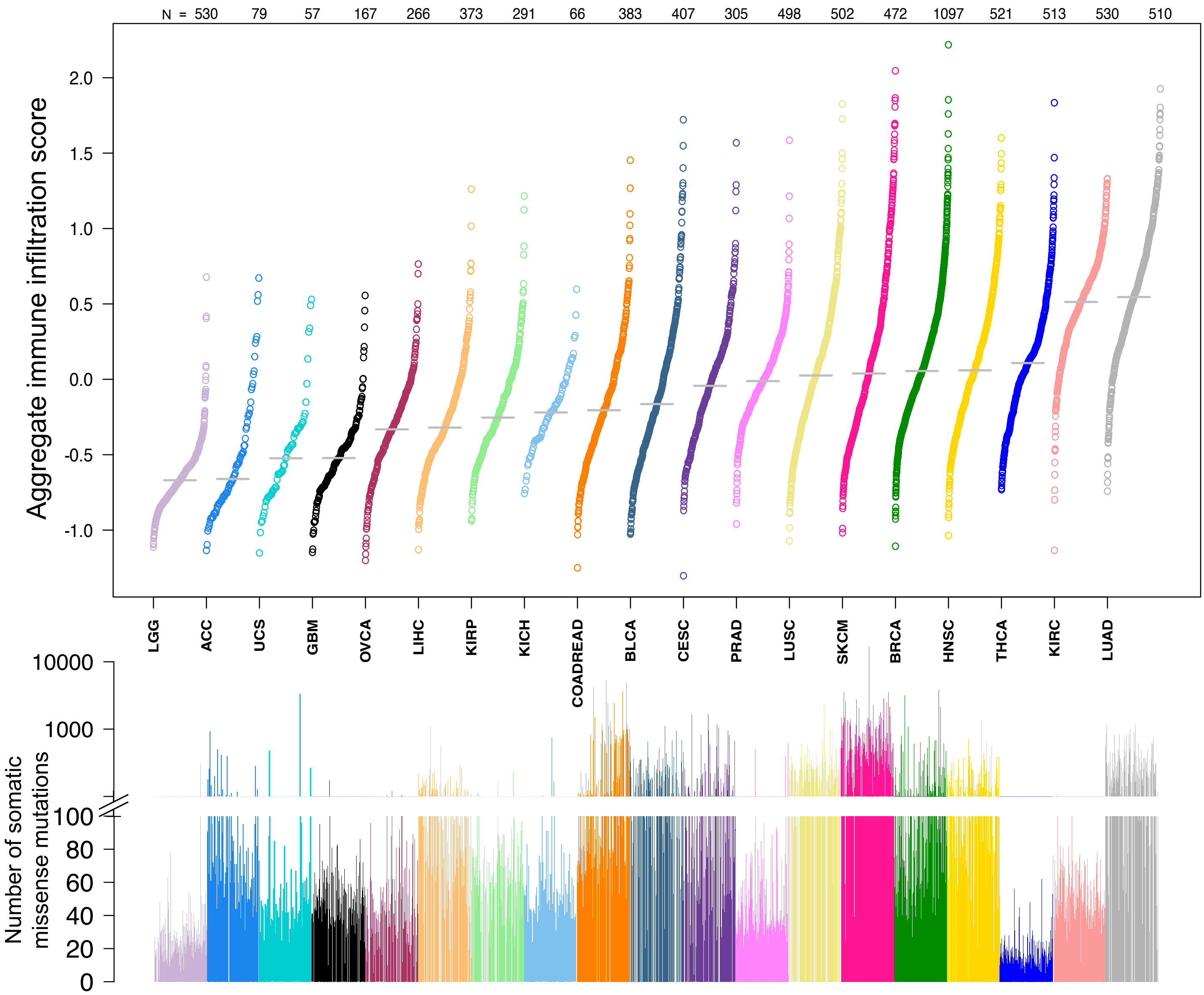

Figure S8. Pan-cancer analysis of overall immune infiltration score (IIS). Overall immune infiltration score (IIS) (top panel) and the corresponding total number of somatic missense mutations (bottom panel) for 19 tumor types. Each dot represents an individual tumor sample. Tumor types are ordered from left to right according to increasing median IIS (medians indicated by horizontal gray bars).There is little relationship between IIS and the quantity of somatic missense mutations.

Supplementary Figure 9

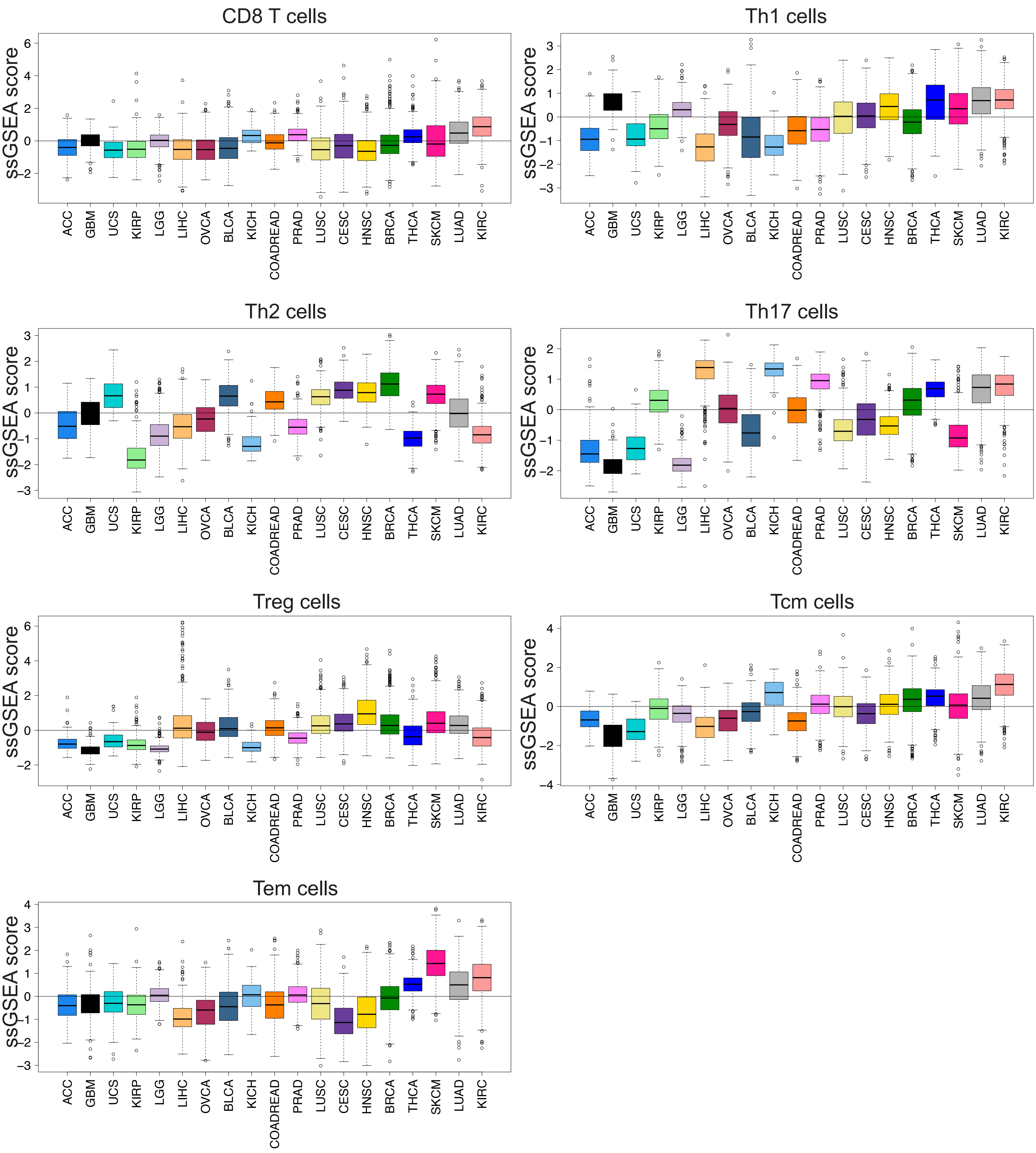

Figure S9. T cell subpopulations used in the aggregate TIS score. Pan-cancer comparisons of T cell subpopulation infiltration levels: CD8 T, Th1, Th2, Th17, Treg, Tcm, and Tem cells (Tcm: T central memory, Tem: T effector memory). The order of tumor types is adopted from the TIS order in Figure 3a.

Supplementary Figure 10

a. Cytolytic index vs Total mutation count
All data

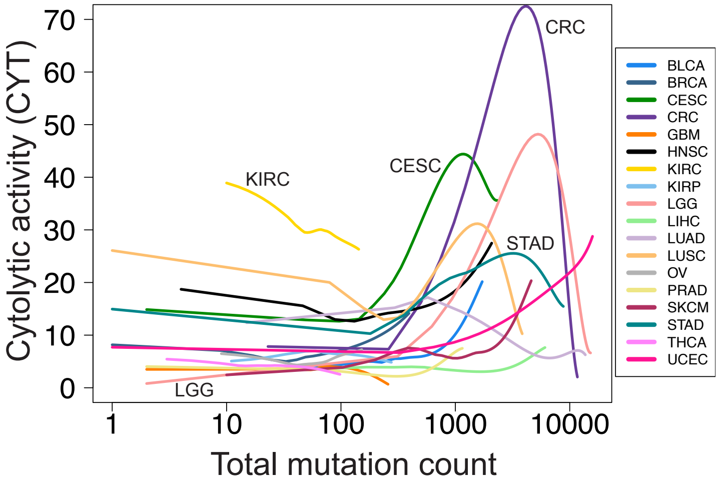

CTYPE	SPEARMAN R	P-VALUE	N	FDR adjusted P-VALUE
BLCA	0.127	0.140967	136	0.230673249
BRCA	0.058	0.1124855	760	0.220164051
CESC	0.184	0.0105416	193	0.03162482
CRC	0.178	0.0087662	217	0.031558304
GBM	0.029	0.7273103	147	0.770093265
HNSC	0.004	0.9444187	294	0.944418682
KIRC	-0.070	0.1617942	406	0.242691317
KIRP	-0.049	0.5292275	167	0.680435366
LGG	0.275	7.85E-05	201	0.001413054
LIHC	-0.038	0.5978837	196	0.717460487
LUAD	0.230	0.0026862	168	0.024176074
LUSC	0.130	0.0853977	176	0.219593999
OV	0.076	0.2976012	188	0.412063164
PRAD	-0.027	0.6696339	258	0.753338173
SKCM	0.163	0.1182719	93	0.220164051
STAD	0.173	0.0049145	263	0.029487249
THCA	-0.087	0.1223134	314	0.220164051
UCEC	0.168	0.0085613	244	0.031558304

b. Cytolytic index vs Total mutation count
From 5th to 95th percentile

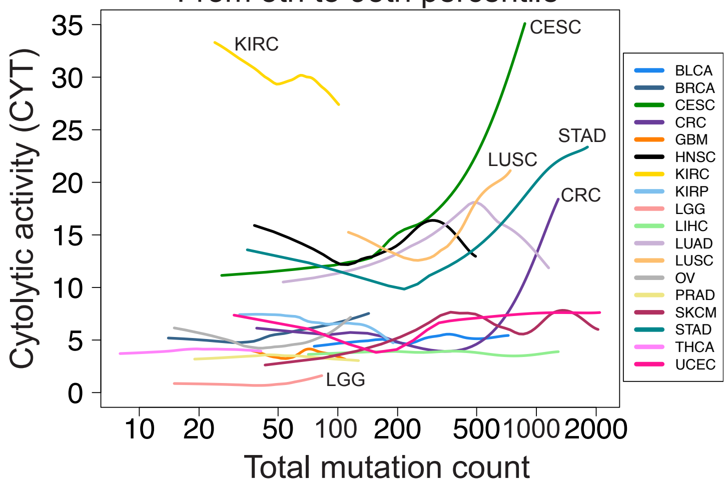

CTYPE	SPEARMAN R	P-VALUE	N	FDR adjusted P-VALUE
BLCA	0.083	0.36620526	122	0.599244967
BRCA	0.099	0.00983373	684	0.038338884
CESC	0.172	0.02332047	173	0.0699614
CRC	0.183	0.01064969	195	0.038338884
GBM	0.055	0.53279187	131	0.64540174
HNSC	0.009	0.88626338	265	0.886263379
KIRC	-0.036	0.48882526	365	0.64540174
KIRP	-0.067	0.40983564	152	0.614753456
LGG	0.225	0.00228847	181	0.038338884
LIHC	-0.035	0.64634248	176	0.727135292
LUAD	0.219	0.00722622	150	0.038338884
LUSC	0.143	0.07288714	159	0.174207406
OV	0.073	0.34495165	170	0.599244967
PRAD	-0.026	0.6890309	233	0.72956213
SKCM	0.172	0.12089467	83	0.24178933
STAD	0.173	0.00791566	235	0.038338884
THCA	-0.037	0.53783478	283	0.64540174
UCEC	0.119	0.07742551	220	0.174207406

c. Cytolytic index vs Missense mutation count
All data

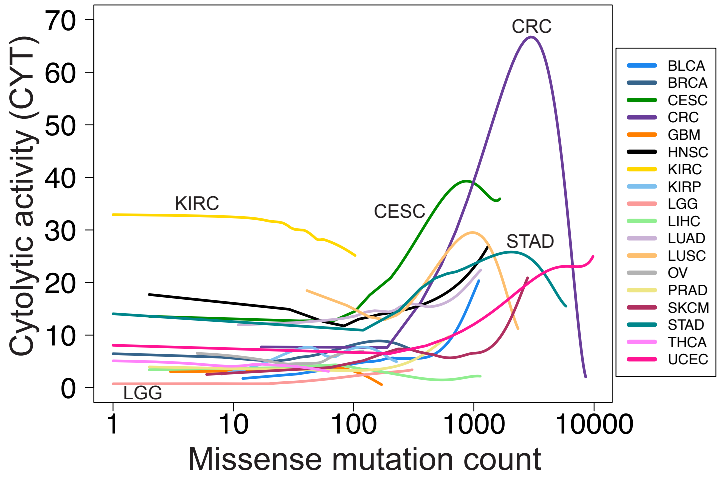

CTYPE	SPEARMAN R	P-VALUE	N	FDR adjusted P-VALUE
BLCA	0.174	0.0483568	129	0.087042291
BRCA	0.078	0.0324694	747	0.073056058
CESC	0.184	0.0112564	190	0.05401169
CRC	0.170	0.0120026	217	0.05401169
GBM	0.030	0.7204445	147	0.720444482
HNSC	0.033	0.5948809	270	0.669241023
KIRC	-0.096	0.0575921	392	0.094241554
KIRP	-0.072	0.3636483	160	0.436377953
LGG	0.278	9.21E-05	192	0.00165795
LIHC	-0.172	0.0166488	193	0.057829417
LUAD	0.184	0.0391102	126	0.078220371
LUSC	0.168	0.0270049	174	0.069441254
OV	0.068	0.3516601	188	0.436377953
PRAD	-0.030	0.6322553	258	0.669446765
SKCM	0.162	0.1210953	93	0.181642993
STAD	0.241	0.0006771	196	0.006093713
THCA	-0.067	0.2365885	312	0.327584107
UCEC	0.150	0.0192765	244	0.057829417

d. Cytolytic index vs Missense mutation count
From 5th to 95th percentile

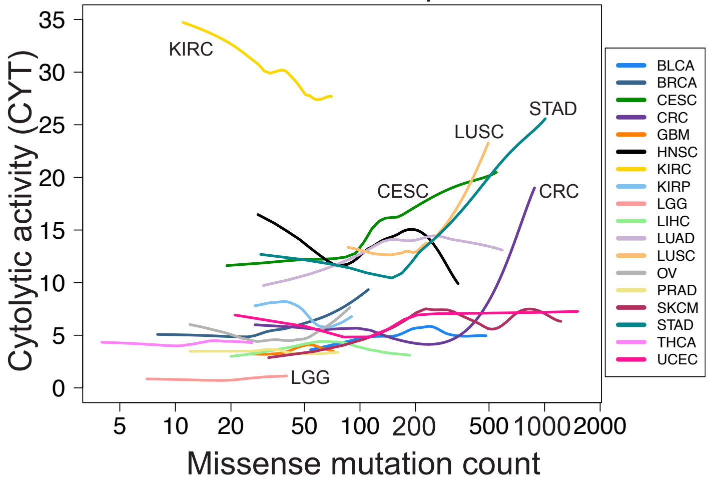

CTYPE	SPERMAN R	P-VALUE	N	FDR adjusted P-VALUE
BLCA	0.105	0.26194165	115	0.336782117
BRCA	0.114	0.00286943	680	0.025824859
CESC	0.149	0.0527055	170	0.158116486
CRC	0.190	0.00782021	195	0.035190946
GBM	0.104	0.23597264	131	0.326731343
HNSC	0.003	0.96400108	244	0.964001076
KIRC	-0.068	0.20464324	352	0.306964859
KIRP	-0.150	0.07193297	144	0.161849181
LGG	0.201	0.00762423	176	0.035190946
LIHC	-0.117	0.12427578	173	0.203902288
LUAD	0.146	0.12351287	112	0.203902288
LUSC	0.177	0.02732257	156	0.098361238
OV	0.055	0.47687753	169	0.572253034
PRAD	-0.041	0.53808469	232	0.605345273
SKCM	0.170	0.12460695	83	0.203902288
STAD	0.236	0.00159275	177	0.025824859
THCA	-0.032	0.59083449	280	0.625589461
UCEC	0.124	0.0676565	219	0.161849181

Figure S10. Local regression curves and correlations between cytolytic activity index (CYT) and mutation counts. (a-b) Local regression curves and correlations between CYT and total mutation count **(a)** when the entire range of the mutation data is used **(b)** when only the 9th to 95th percentile of the mutation data is used as implemented in [13]. **(c-d)** Local regression curves and correlations between CYT and number of somatic missense mutations **(c)** when the entire range of the mutation data is used **(d)** when only the 9th to 95th percentile of the mutation data is used. Cancer types where the correlations is significantly different from zero at 0.05 alpha level after multiple hypothesis correction are highlighted in green. CYT and total mutation data were obtained from [13], and missense mutation data were obtained from the Feb 4, 2015 output of Firehose.

Supplementary Figure 11

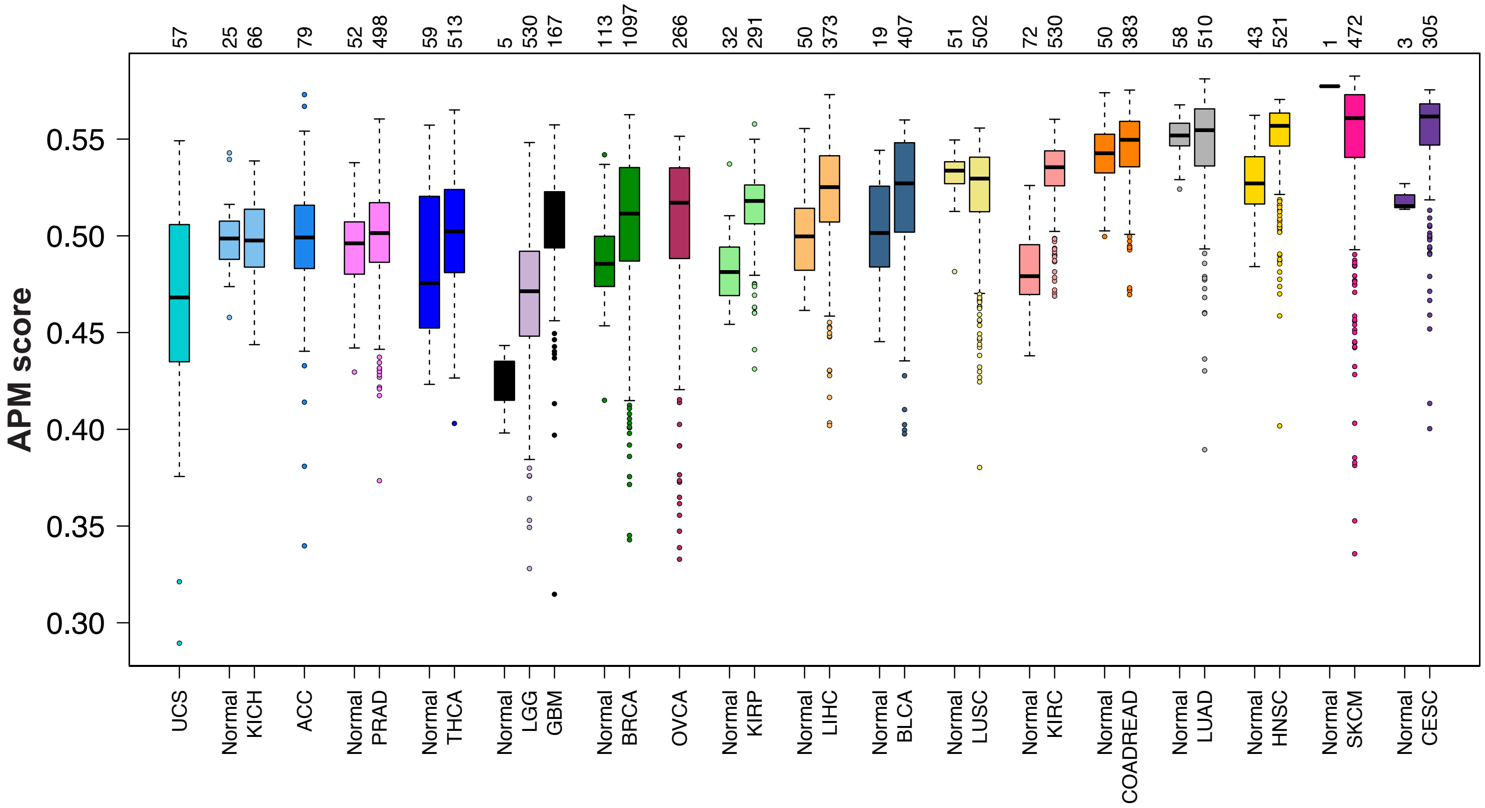

Cancer Type	FDR-adjusted p-value
ACC	NA
**BLCA**	**6.6E-03**
**BRCA**	**1.6E-14**
**CESC**	**1.6E-02**
**COADREAD**	**3.4E-02**
**GBM**	**6.9E-04**
**HNSC**	**1.3E-12**
KICH	8.7E-01
**KIRC**	**3.1E-38**
**KIRP**	**2.7E-13**
**LGG**	**3.6E-03**
**LIHC**	**5.1E-08**
LUAD	5.9E-01
LUSC	8.9E-02
OVCA	NA
**PRAD**	**1.2E-02**
SKCM	NA
**THCA**	**1.6E-03**
UCS	NA

Figure S11. Pan-cancer tumor-normal differences for APM. APM scores for cancer types are shown with boxplots adjacent to the relevant normal tissue. Tumor-normal differences are compared with Mann-Whitney tests, and p-values are corrected for multiple hypothesis testing with the Benjamini & Hochberg method. TCGA RNA-seq data are used for the analysis.

Supplementary Figure 12

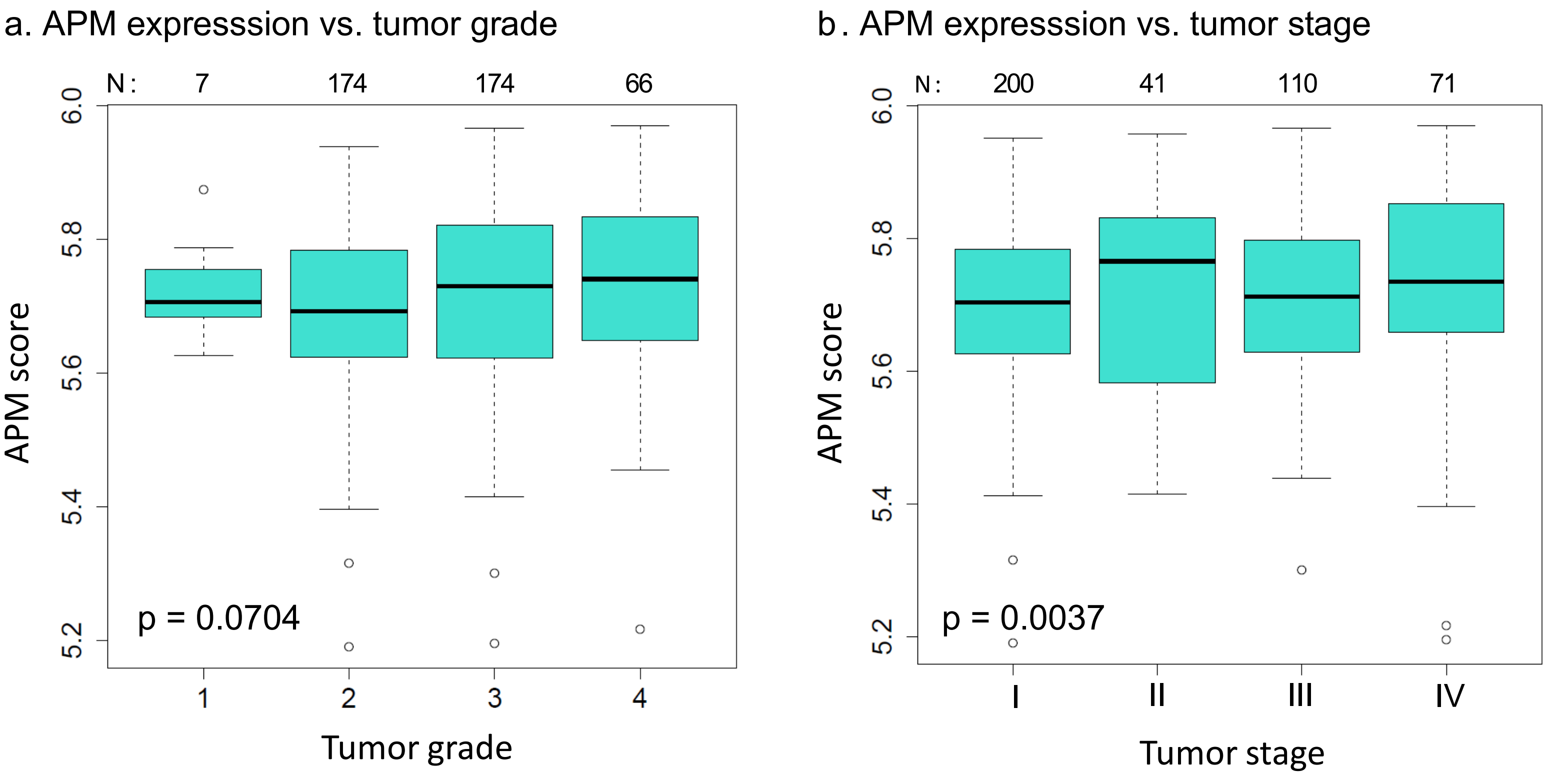

Figure S12. Grade– and stage–specific APM expression. We investigated the association of antigen presentation machinery gene expression with (a) tumor grade and (b) tumor stage. Even though the grade and stage groups showed significant differences at $\alpha=0.1$ ($p = 0.07$ and 0.004 respectively, ANOVA), a linear trend between APM and these variables does not exist. A positive association could suggest that APM expression increases with necrosis.

Supplementary Figure 13

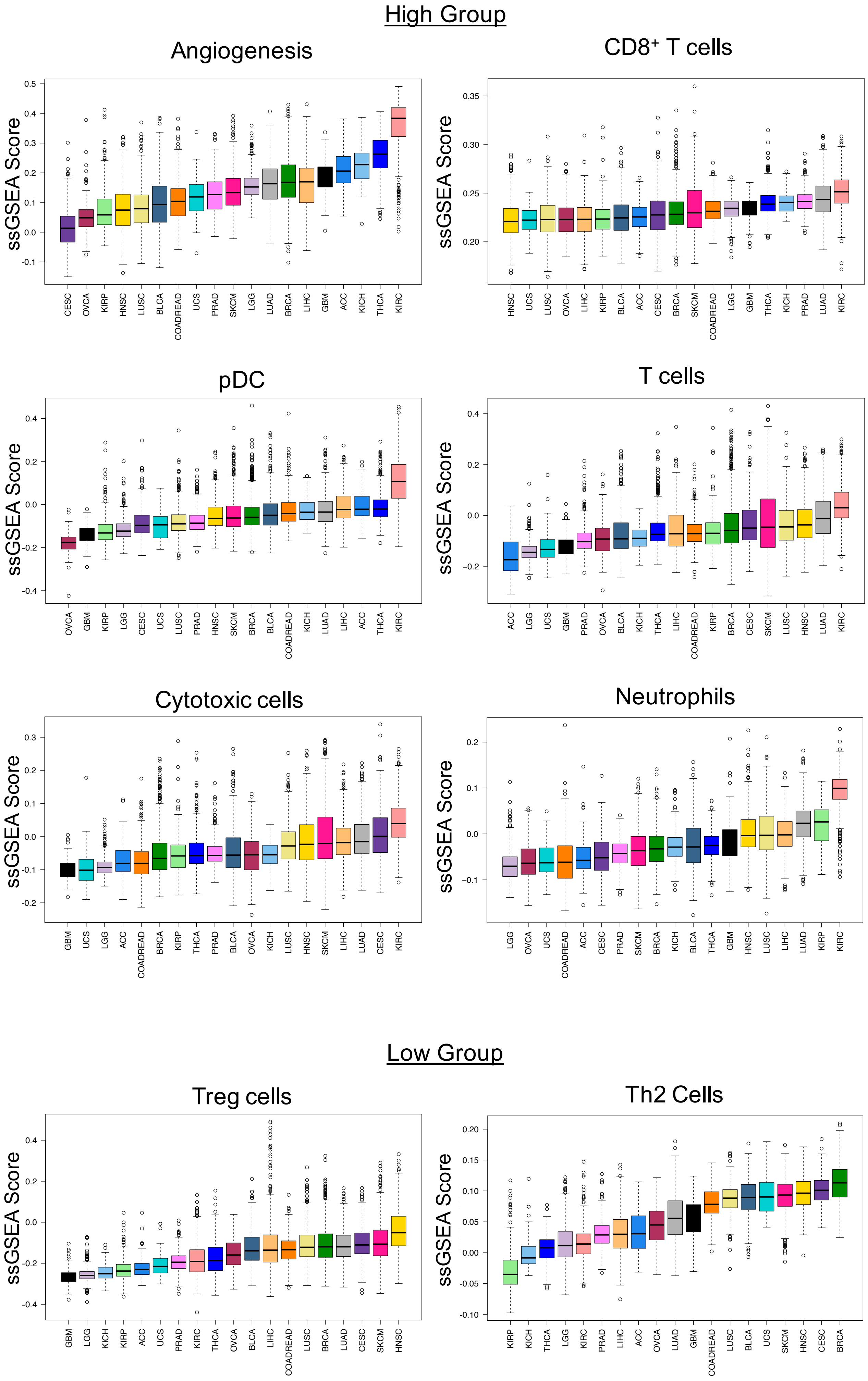

Figure S13. The signatures where ccRCC is among the highest or lowest across 19 cancer types. Analysis of immune cell and angiogenesis levels across 19 human cancers. ccRCC tumors stand out from others by having elevated levels of angiogenesis, several T cell signatures (T cells, $CD8^{+}$ T cells) along with pDCs, cytotoxic cells and neutrophils. ccRCC tumors are relatively poorly infiltrated by Tregs and Th2 cells.

Supplementary Figure 14

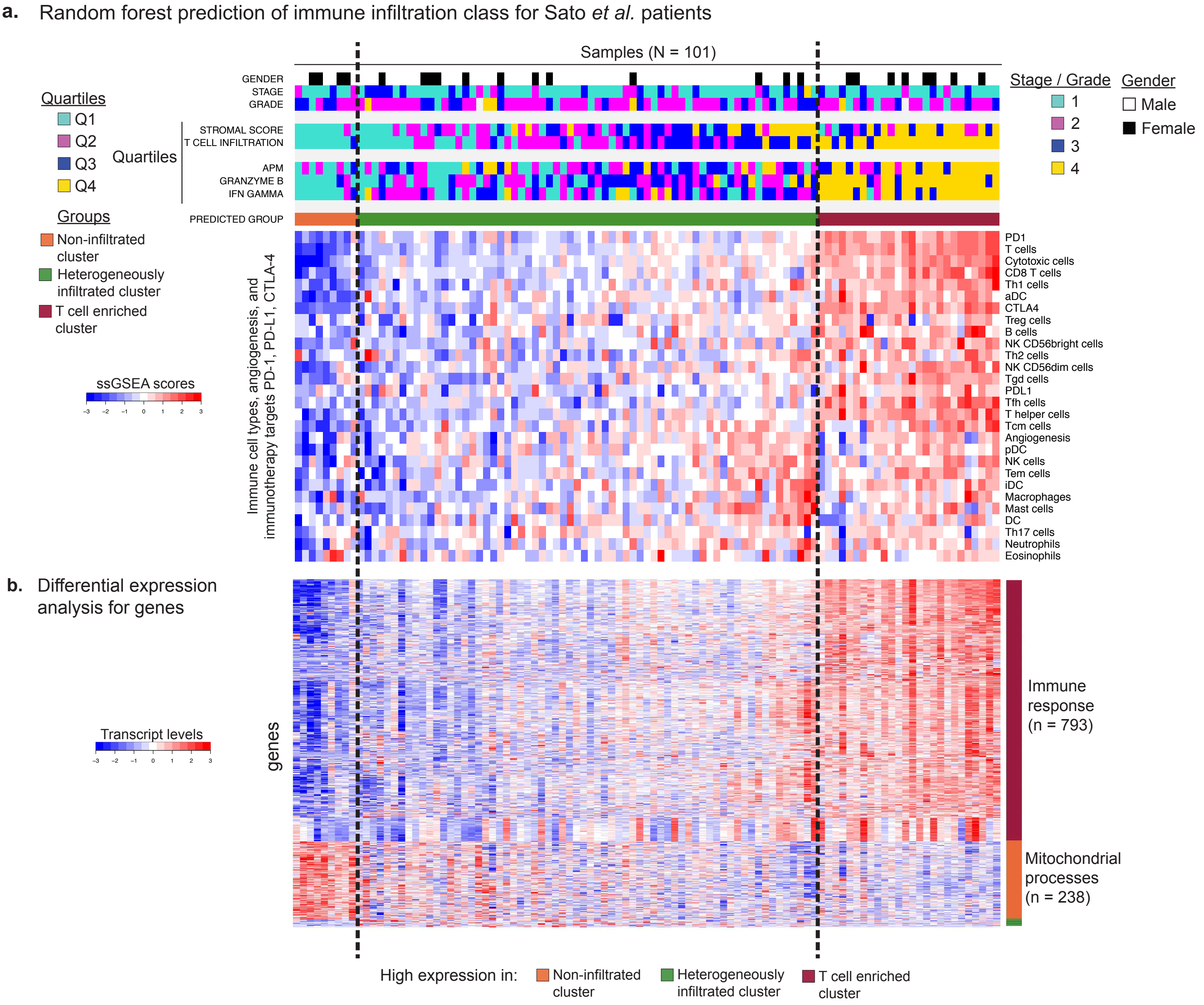

Figure S14. Validation of ccRCC immune infiltration classes with the SATO dataset. (a) A random forest classifier trained on the TCGA ccRCC cohort was used to predict the immune infiltration class for 101 patients in the SATO cohort. As was observed in TCGA ccRCC tumors (Figure 5a), T cell enriched tumors show higher expression of antigen presentation machinery genes, granyzme B and interferon gamma. The order of samples in each class from left to right is by increasing immune infiltration score (IIS). The order along the y-axis is adopted from the TCGA ccRCC heatmap in Figure 5a. (b) Heatmap of genes overexpressed in each immune infiltration class (p-value threshold 0.01). The order along the y-axis is obtained by hierarchical clustering with Euclidean distance and Ward linkage. DAVID gene set enrichment analysis reveals that T cell enriched tumors have overexpression of immune response genes while non-infiltrated tumors have overexpression of mitochondrial genes. These results validate the findings in the TCGA ccRCC cohort.

Supplementary Figure 15

a. Subclustering within the T cell enriched cohort

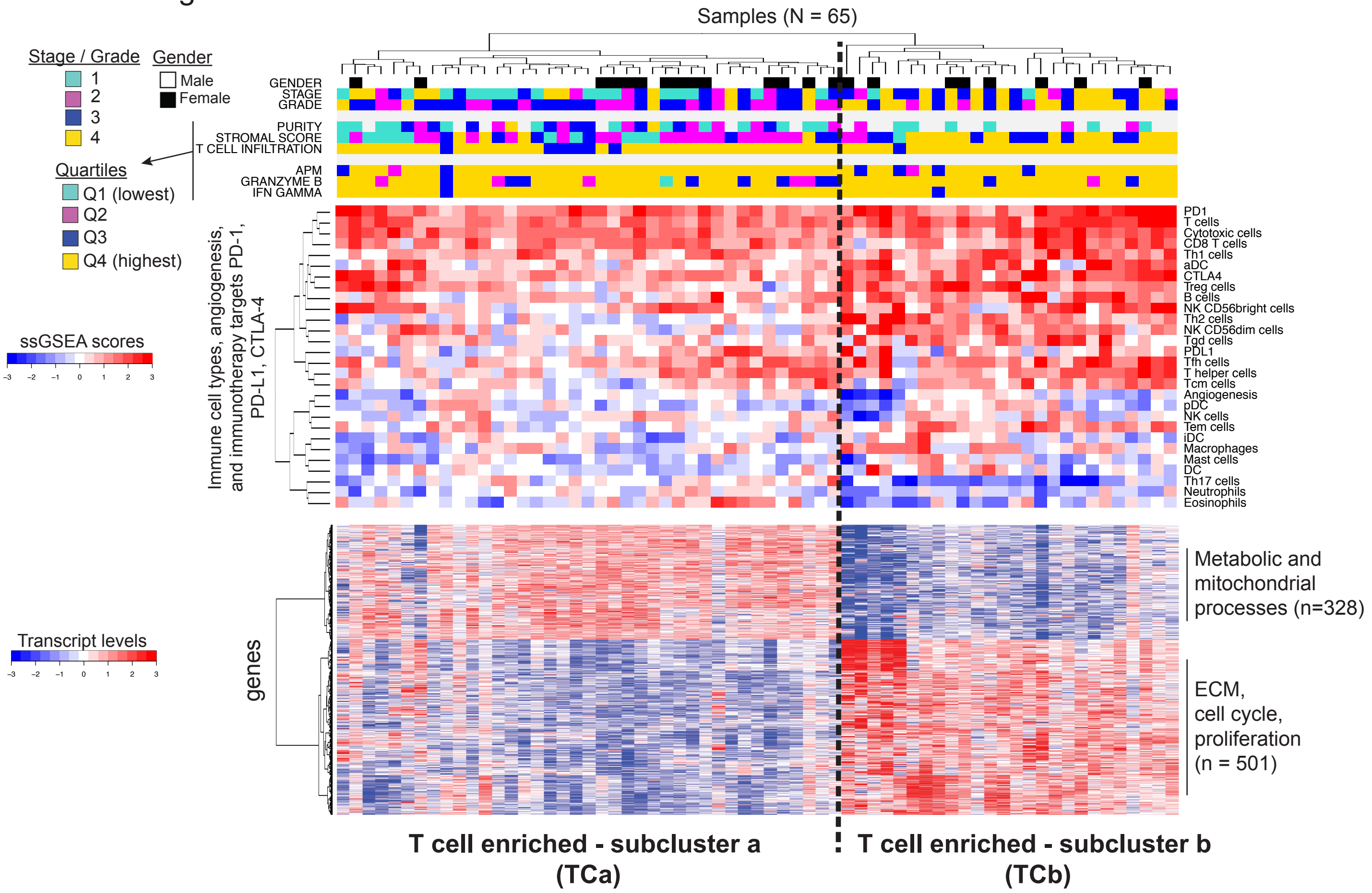

b. Network analysis for overexpressed genes in TCa and TCb

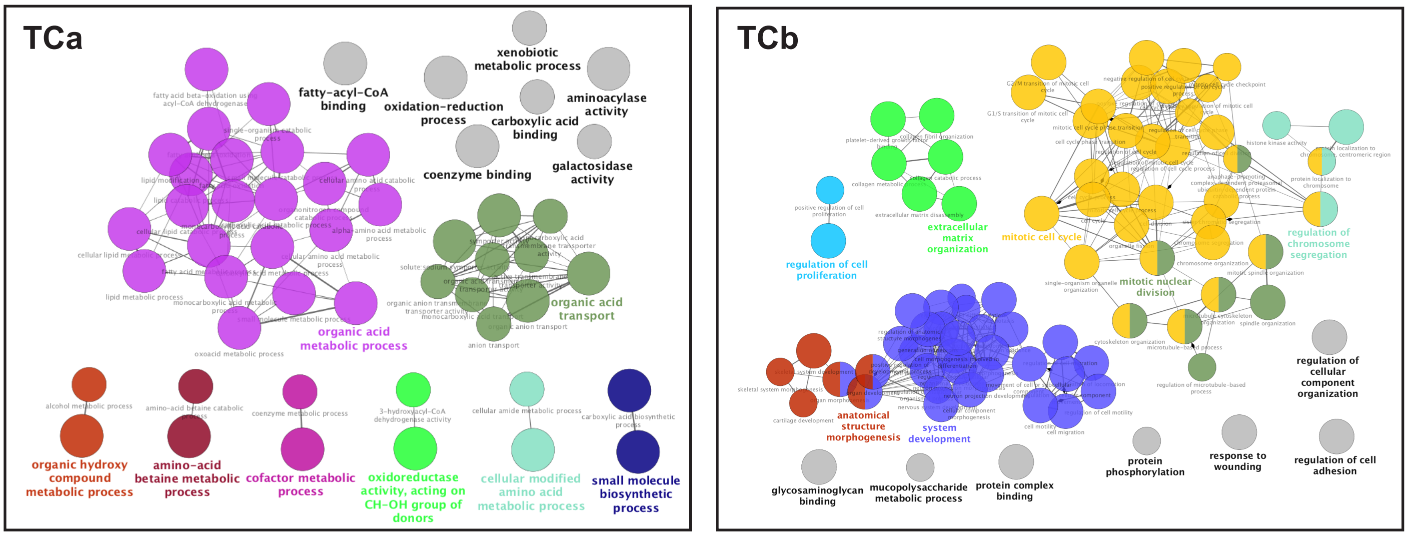

c. Kaplan-Meier curves for cancer-specific survival in TCa and TCb

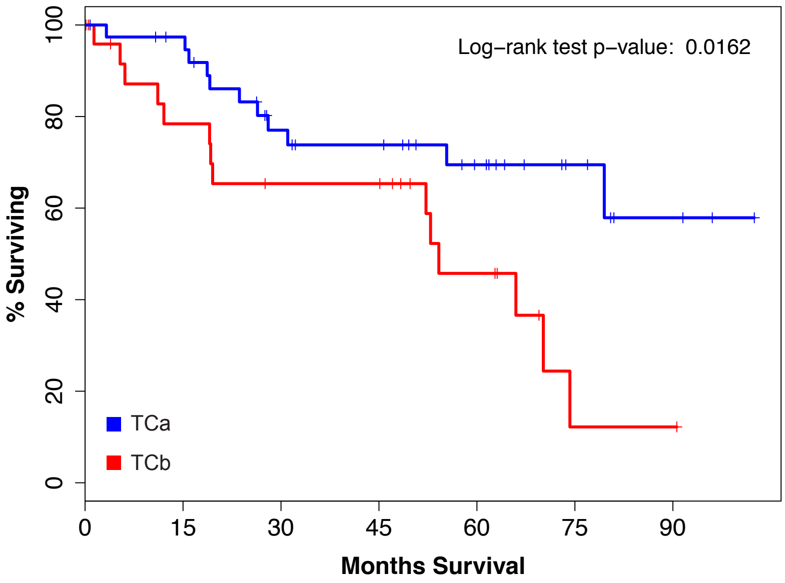

Figure S15. Subclustering within the T cell enriched cohort demonstrates gene expression and survival differences. (a) Hierarchical clustering within the T cell enriched cohort revealed two distinct subclusters, here termed TCa and TCb, that had differences in immune cell levels such as macrophages as well as in grade, stage, and stromal score (top panel). Hierarchical clustering was performed with Euclidean distance and Ward linkage. Differential gene expression analysis was performed with Mann-Whitney tests (bottom panel). Only genes that are significantly overexpressed in one cluster at a q-value cutoff of $5x10^{-5}$ are shown. Pathway analysis using DAVID[44] reveals that the genes overexpressed in TCa and TCb (N = 328 and 501 respectively) are enriched in 1) metabolic and mitochondrial processes; and 2) extracellular matrix (ECM), cell cycle and cell proliferation respectively. **(b)** Network analysis with ClueGO[50] highlights the upregulation of metabolic processes in TCa, and the upregulation of ECM, cell cycle, cell proliferation in TCb. **(c)** Kaplan-Meier curves for cancer-specific survival in the TCa and TCb patients. Patients in the TCa subcluster have significantly better survival (log-rank test p-value = 0.016)

Supplementary Figure 16

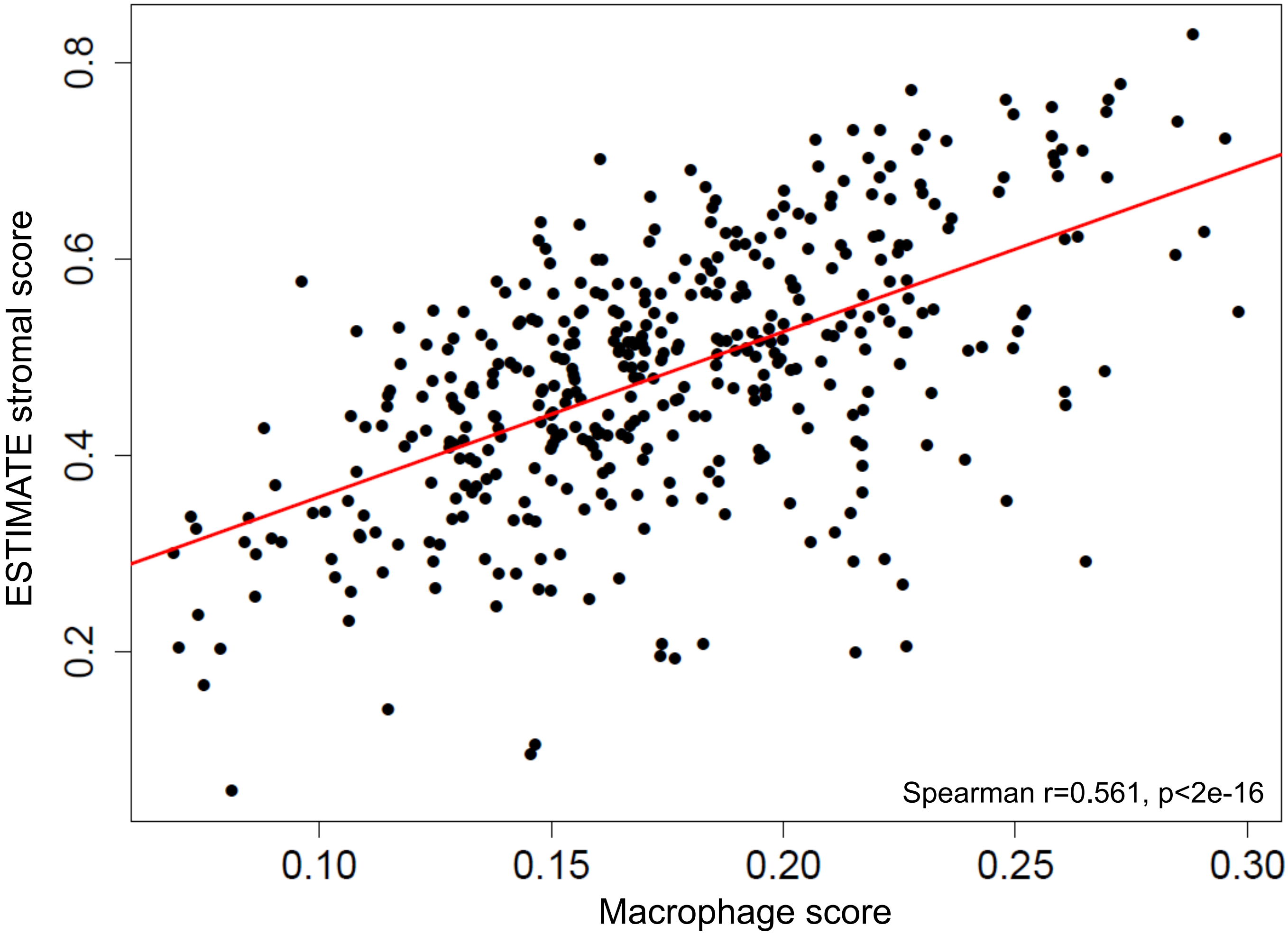

Figure S16. The correlation between the macrophage and ESTIMATE stromal scores in ccRCC. We investigated the association between the macrophage scores in ccRCC and the stromal scores calculated with the gene signature in ESTIMATE. These scores were positively correlated across the entire TCGA ccRCC cohort (Spearman r = 0.561, $p < 2x10^{-16}$).

Supplementary Figure 17

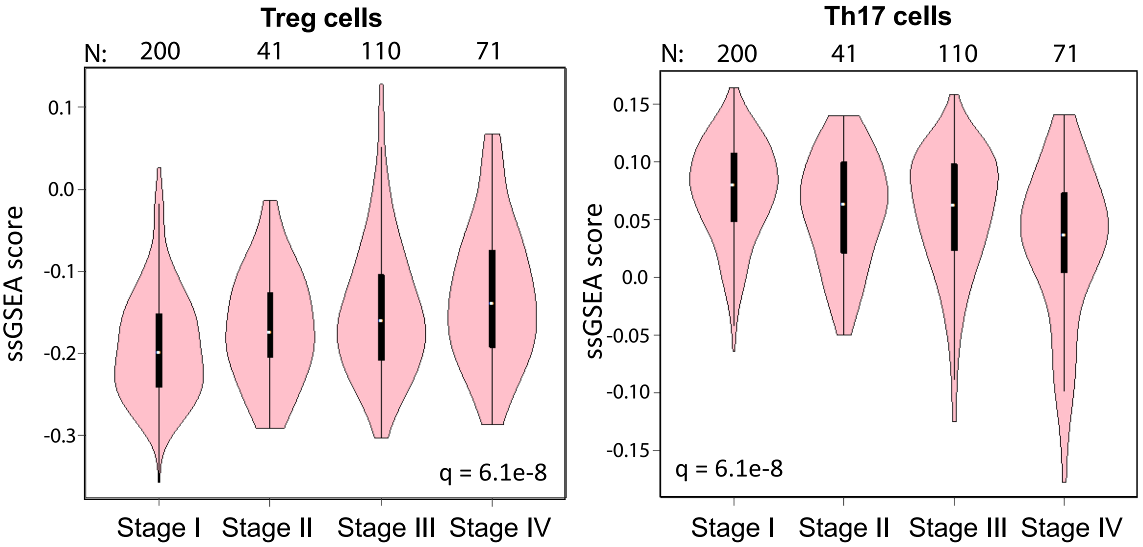

Figure S17. Significant associations between immune cell infiltration levels and tumor stage. Tumor stage is positively associated with Treg cell infiltration (left panel) and negatively associated with Th17 cells (right panel) in the TCGA ccRCC cohort. Adjusted p (i.e. q) values are shown here as all 24 immune cell types were tested against stage.

Supplementary Figure 18

a. TCGA

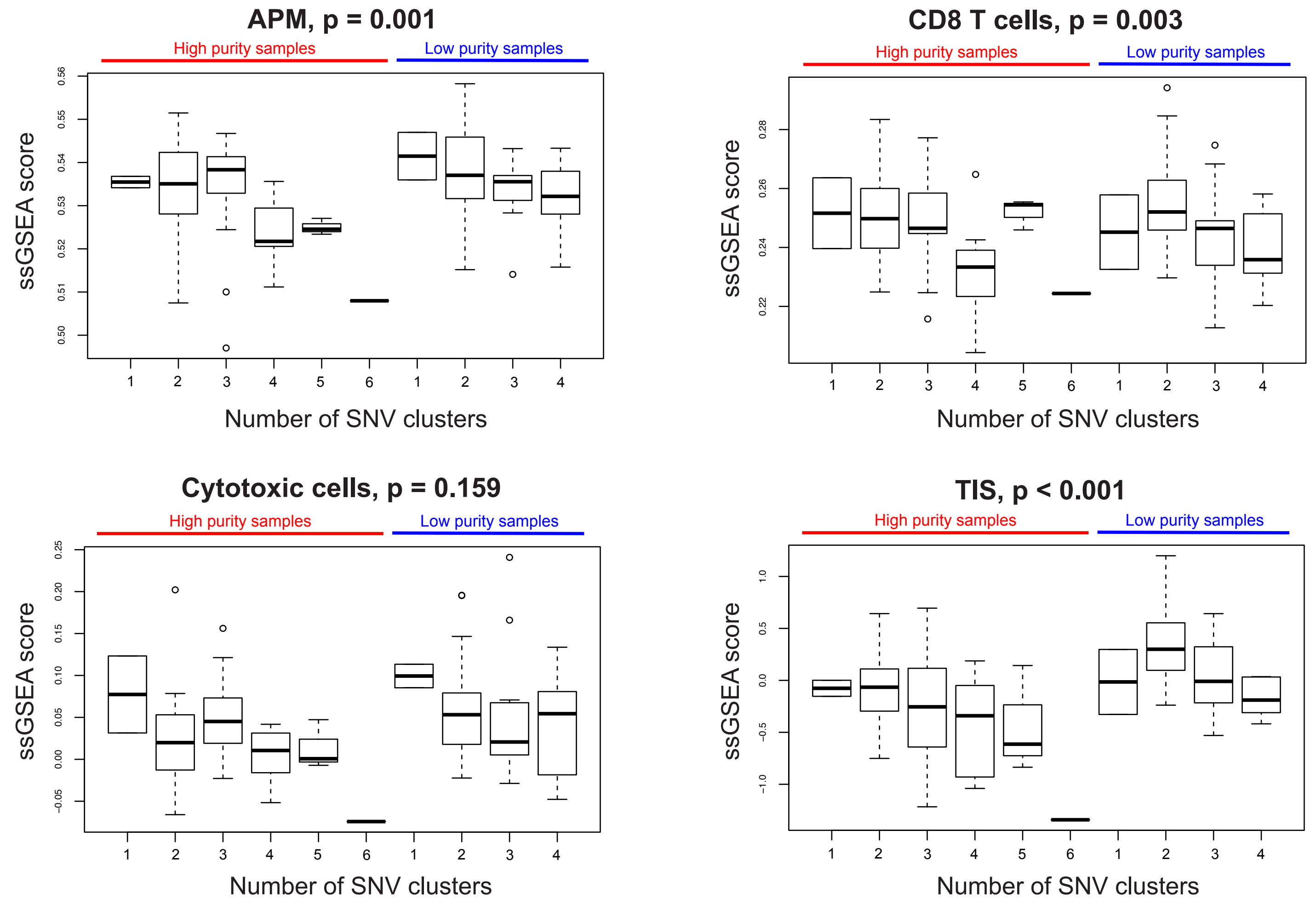

b. SATO

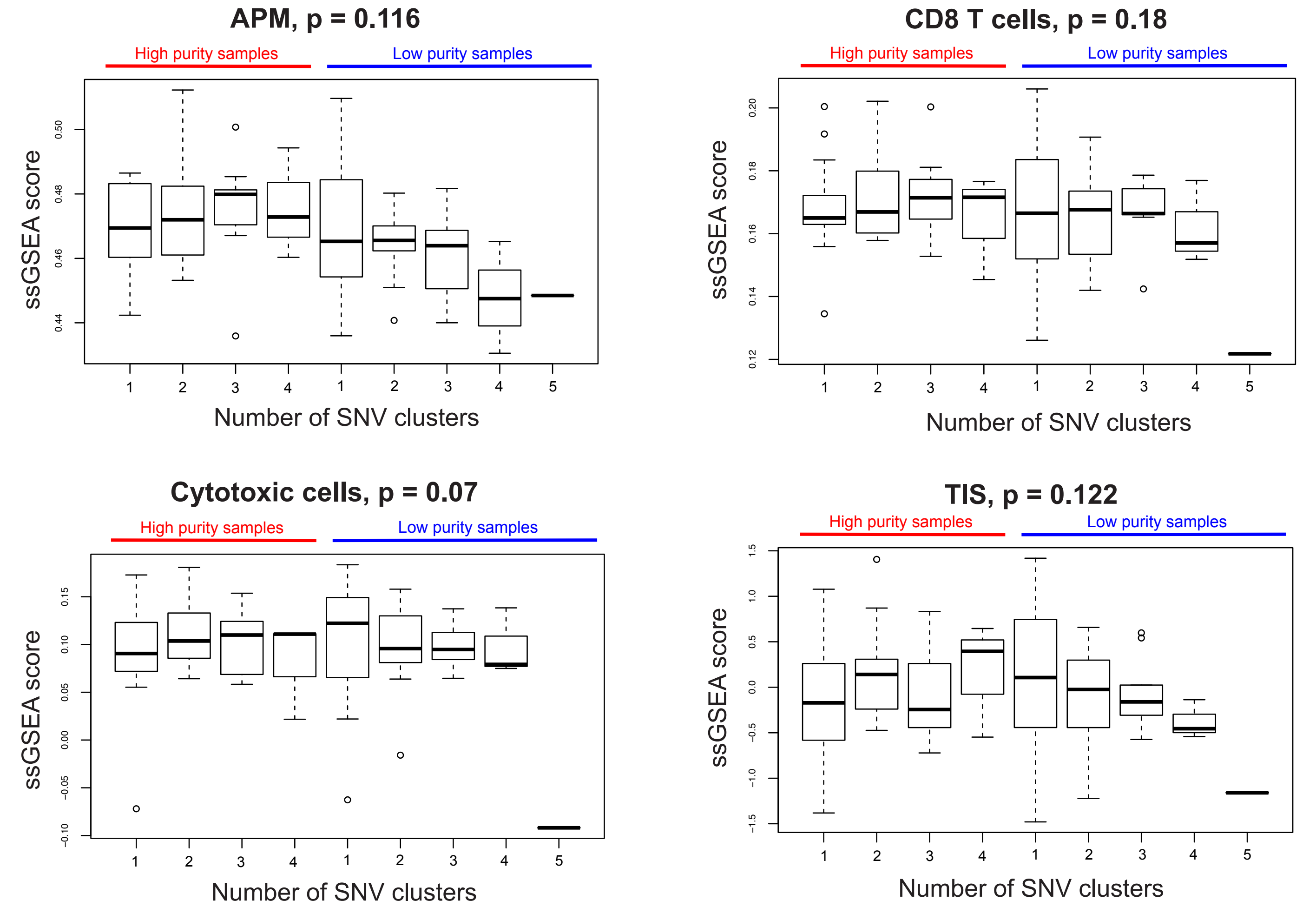

Figure S18. Immune cell score differences in gene expression-based ccRCC subtypes ccA (N=205) and ccB (N=175). Association of immune cell scores with previously defined molecular ccRCC subtypes (ccA and ccB). ccA exhibits significantly higher Th17 and $CD8^{+}$ T cell infiltration levels, but lower scores for Treg and Th2 cells. The former two cell types are associated with improved survival, and the latter two with poor survival (**Figure 6b**). These findings are consistent with reports that showed ccA has better prognosis compared with ccB[56]. Adjusted p (*i.e.* q) values are shown here as all 24 immune cell types were tested against molecular subtype.

Supplementary Figure 19

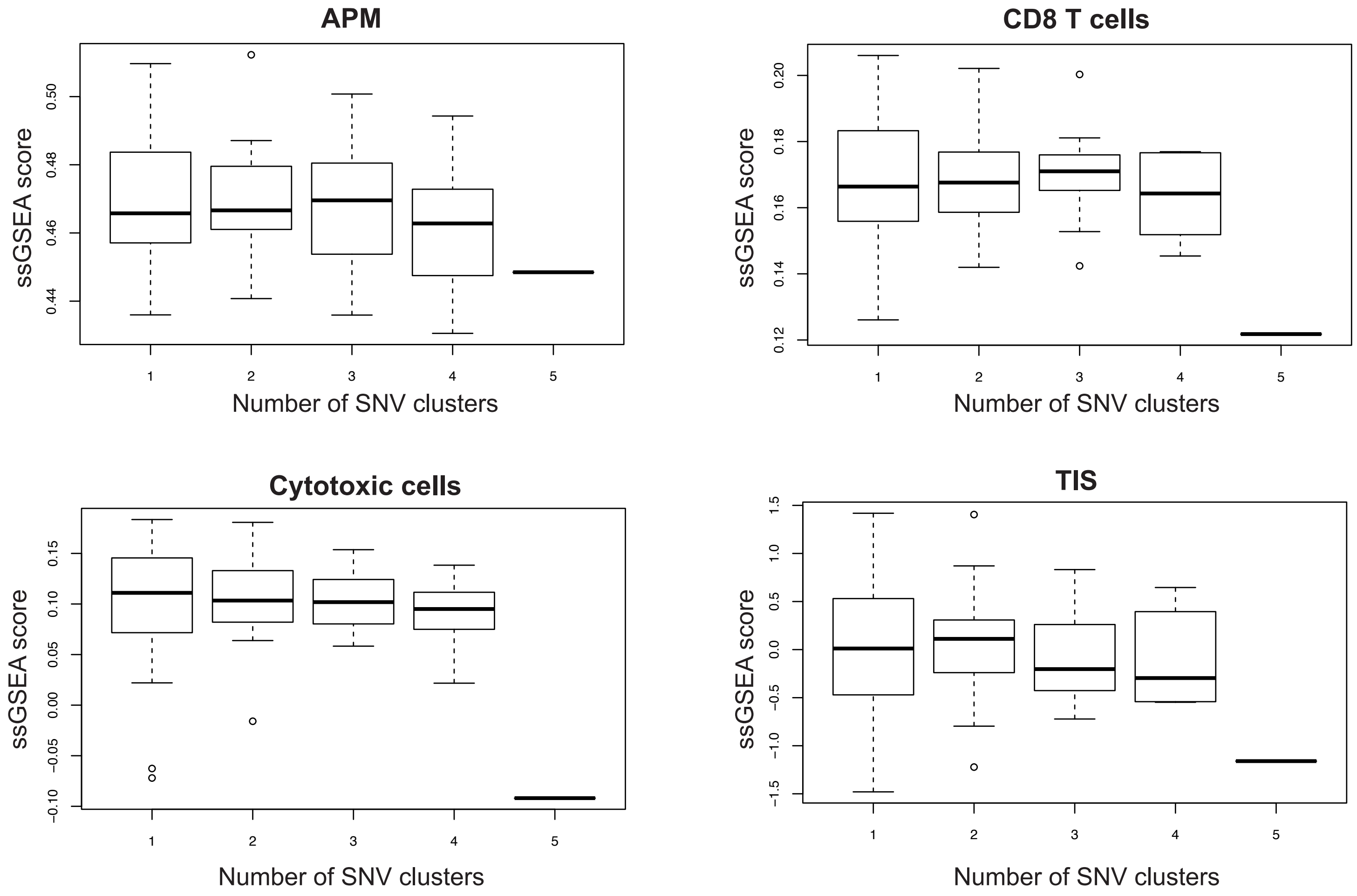

Figure S19. SciClone clonality analysis for SATO samples. The x axis shows the number of single nucleotide variant (SNV) clusters for each tumor where 1 corresponds to clonal tumors and higher number of clusters indicate subclonal architecture. The y axis shows the ssGSEA scores for immune signatures APM, CD8 T cells, cytotoxic cells, and TIS. A trend for an inverse association between immune infiltration and subclonal architecture is observed, although p-values do not reach significance (One-sided p-value = 0.12, 0.18, 0.07, 0.12 respectively). The fraction of samples for each SNV cluster number is 45.8% for 1 cluster (N=44), 26.0% for 2 clusters (N=25), 19.8% for 3 clusters (N=19), 7.3% for 4 clusters (N=7), 1.0% for 5 clusters (N=1).

Supplementary Figure 20

a. TCGA

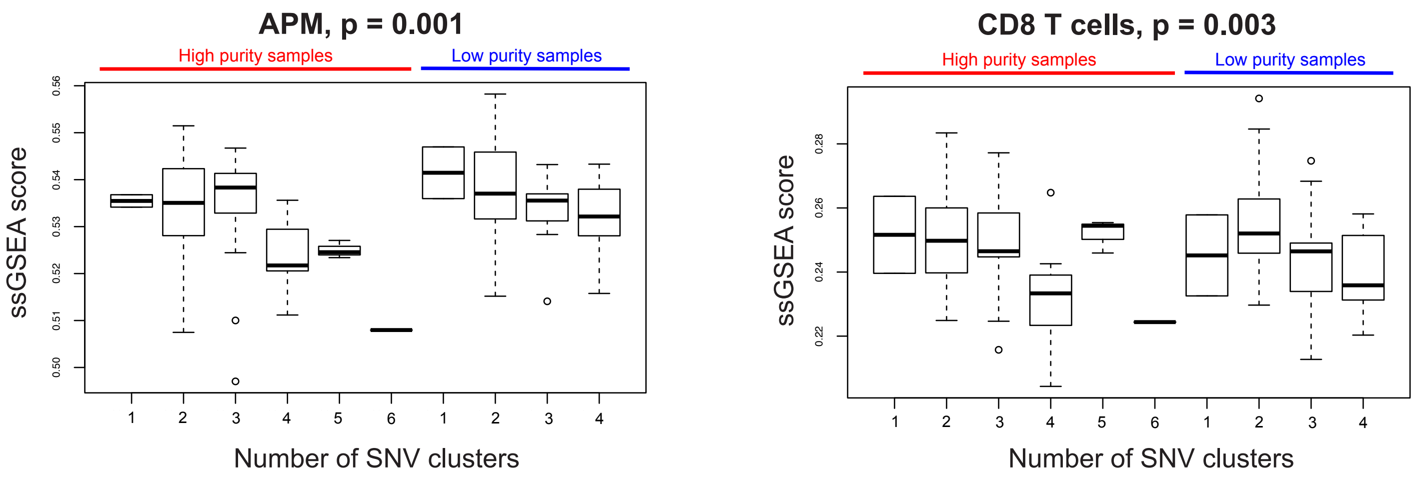

b. SATO

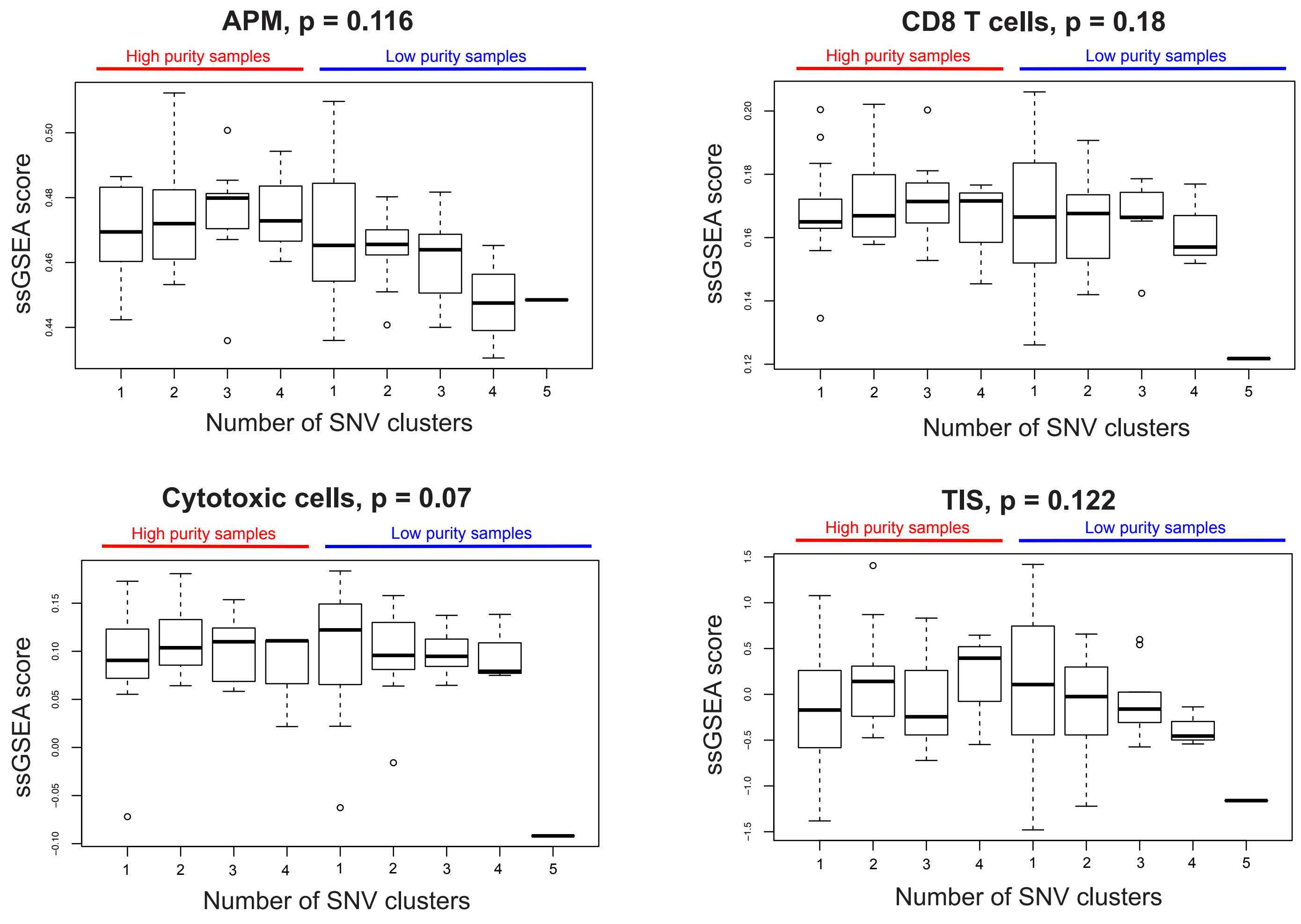

Figure S20. Purity-adjusted clonality analysis for TCGA and SATO samples.
Immune scores are adjusted for purity by regressing immune scores on purity estimates on obtaining the residuals. The association between purity-adjusted immune scores and clonality is investigated with a trend test, and p-values are shown in the subfigure titles. Each subfigure contains two groups of boxplots, one for high purity and and for low purity samples. The axes are the same as in **Figure S19**. (a) TCGA dataset, (b) SATO dataset.

Supplementary Figure 21

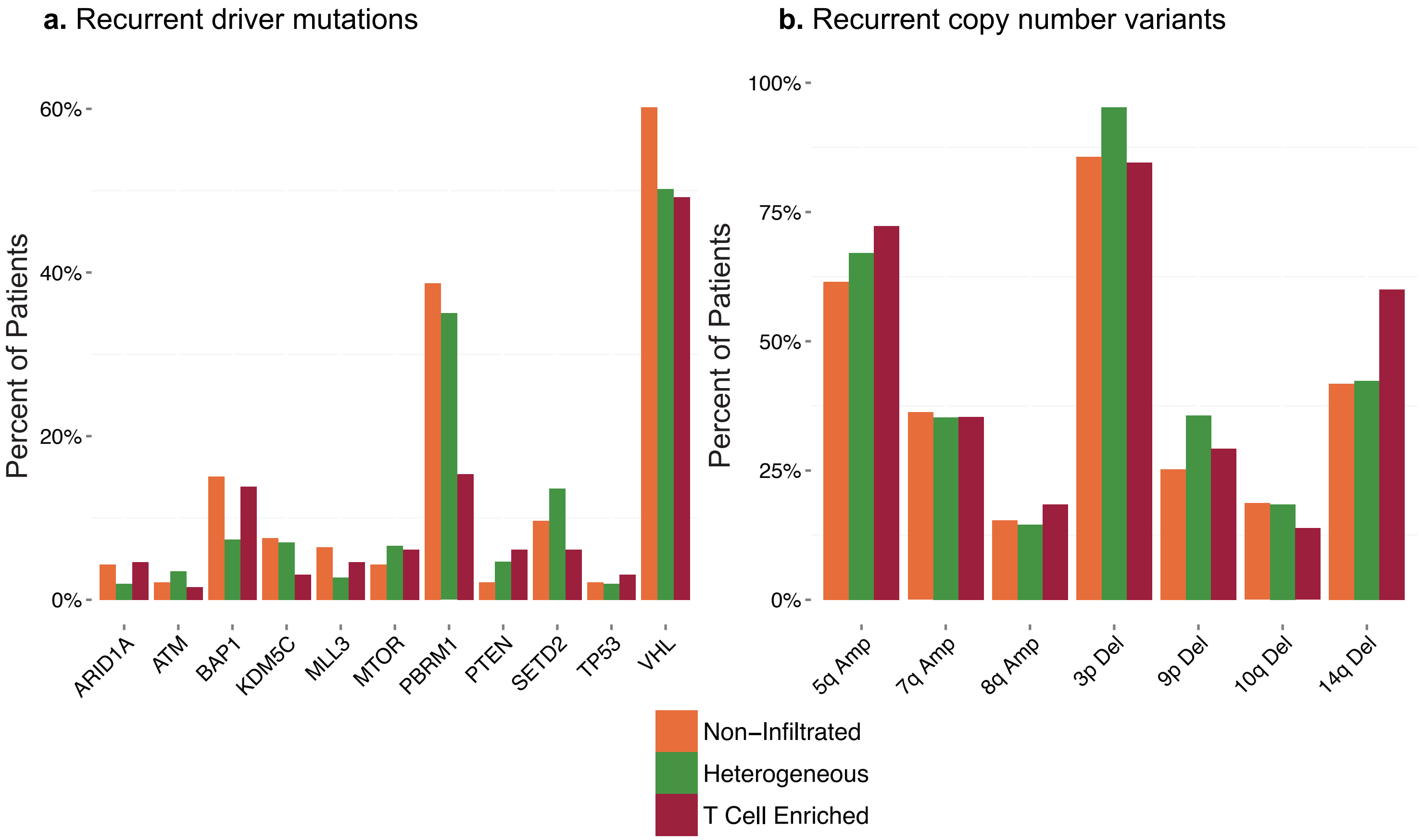

Figure S21. Association of ccRCC immune infiltration classes with recurrent driver mutations and copy number variants

Between the three ccRCC groups, we observed no significant differences in the frequency of recurrent **(a)** driver mutations or **(b)** copy number variants.

Supplementary Figure 22

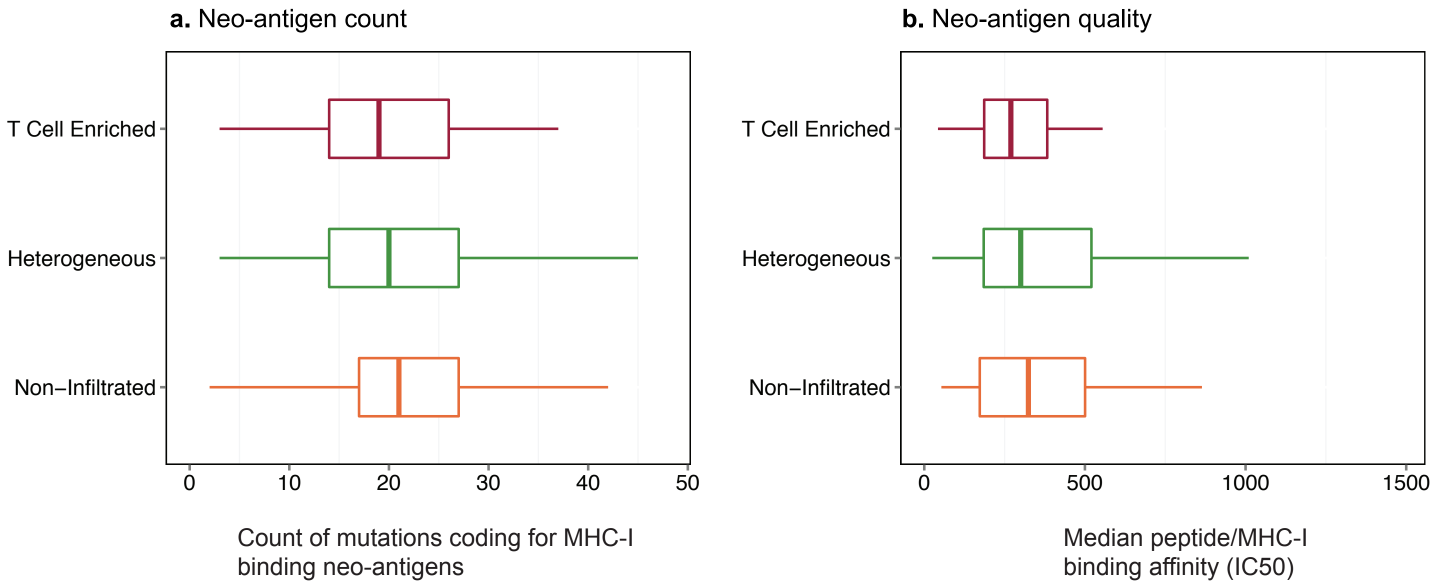

Figure S22. Association of ccRCC immune infiltration classes with neoantigenicity
There were no significant differences between the three ccRCC immune infiltration classes in **(a)** the count of mutations that code for at least one neo-antigen predicted to bind to MHC-I (IC50 < 500nM), or **(b)** the overall quality of neo-antigens. We assessed the overall quality of the neo-antigens found in each cluster by selecting the highest affinity pMHC for each mutation and taking the median of these IC50s (IC50 is inversely related to binding affinity).

Supplementary Figure 23

Noiseless mixture

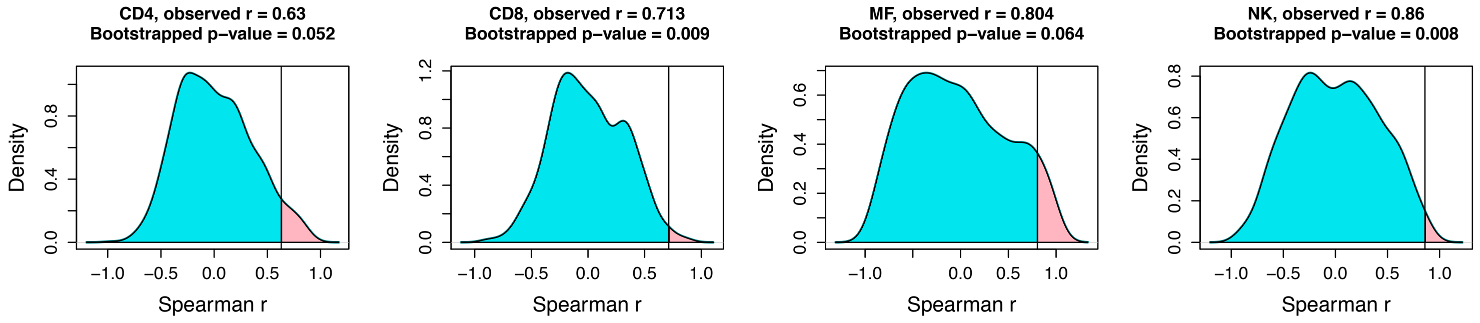

Noisy mixtures

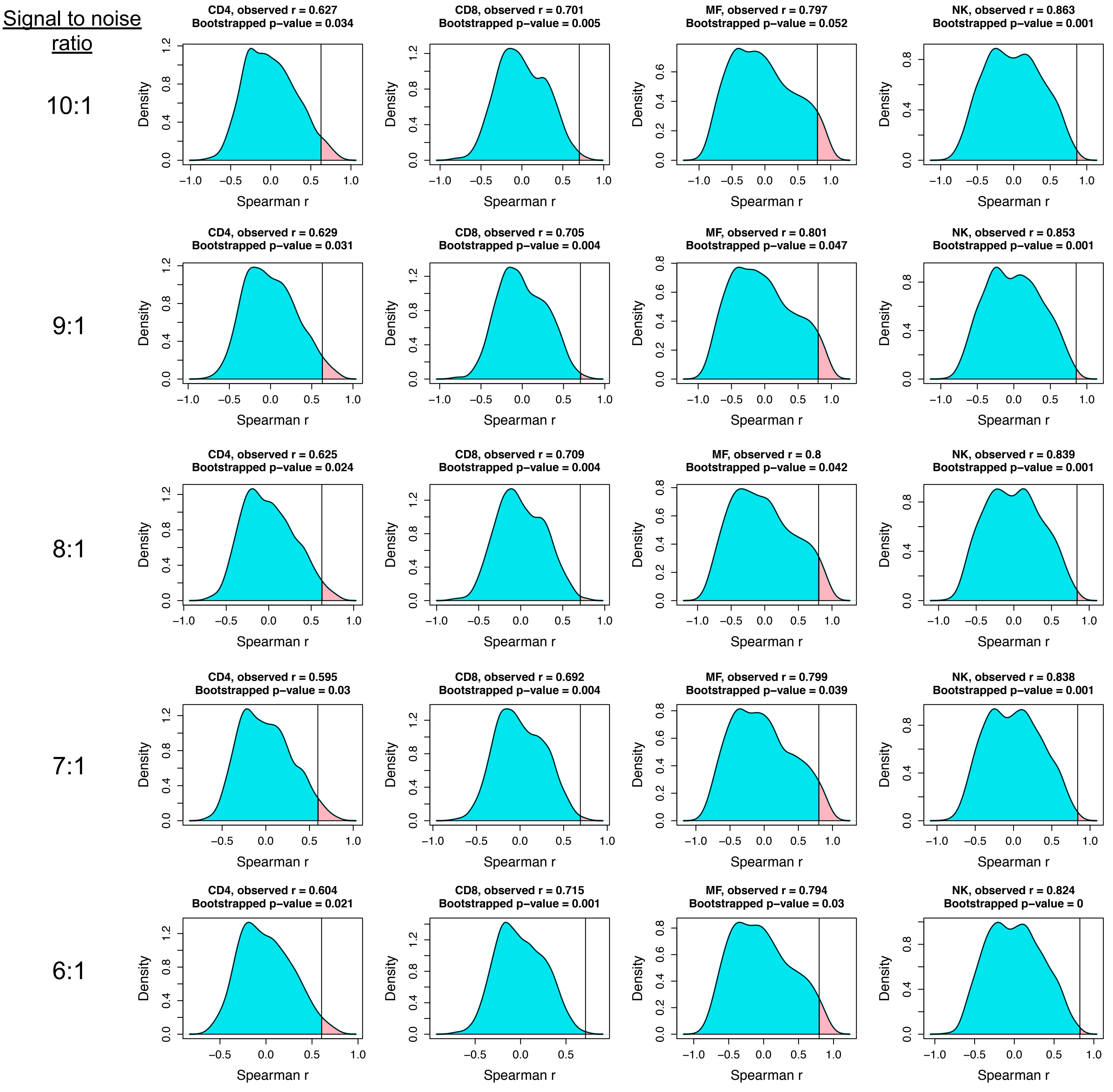

Noisy mixtures

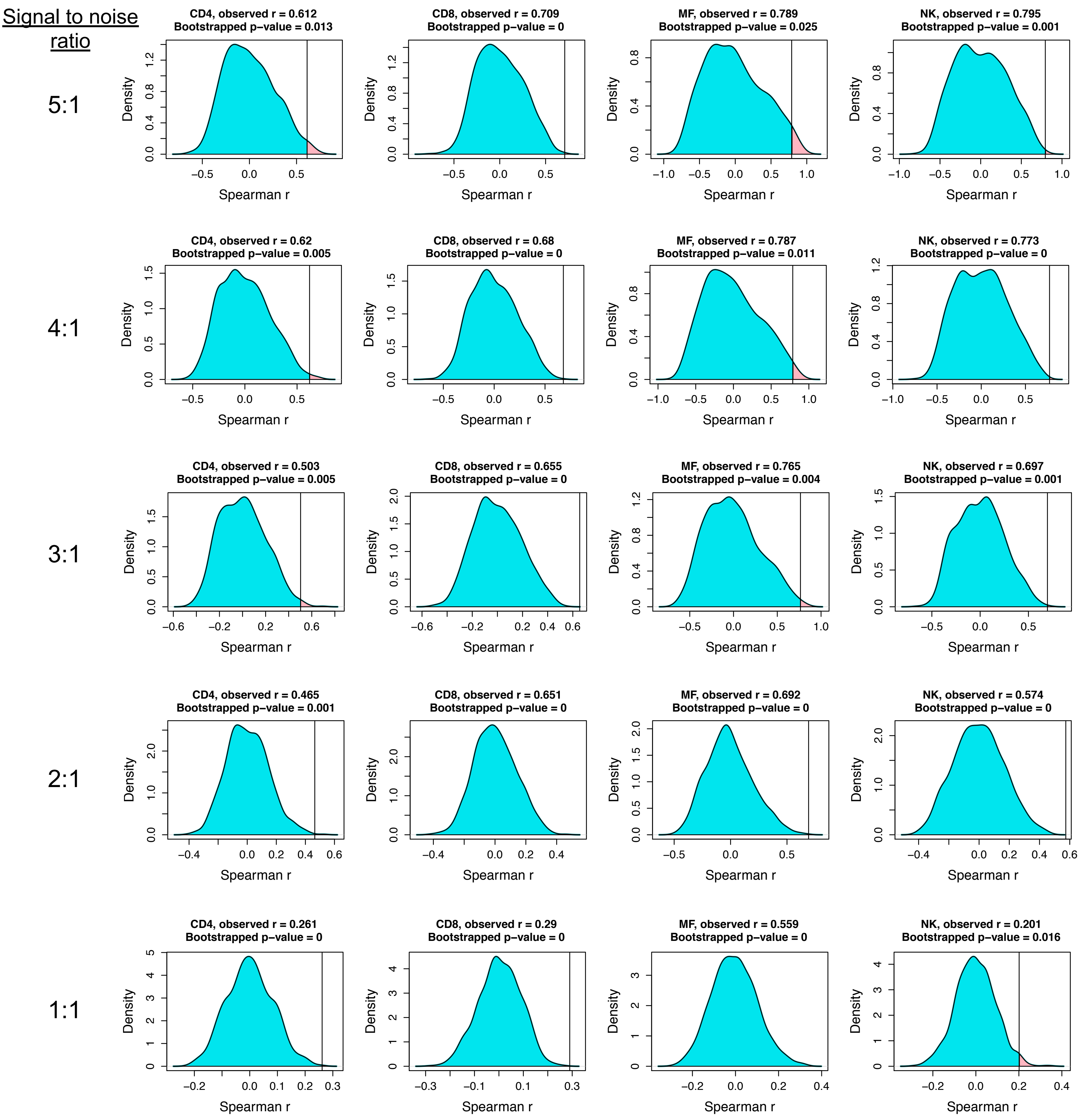

Figure S23. Empirical null distributions for the Spearman correlations between simulated and inferred immune cell levels

ssGSEA was run 1000 times on all *in silico* noiseless and noisy mixture datasets generated for **Figure 2a** top panel, each time with a different set of four random signatures that emulated the signatures for NK cells, macrophages, $CD4^{+}$ and $CD8^{+}$ T cells. Each run inferred the four types of scores for 200 samples, and then a Spearman correlation was computed for each cell type between the inferred scores and the simulated mixing proportions. At each noise level, 1000 correlation values from random bootstrap signatures formed an empirical null distribution (shown with density curves here) for the observed correlation from the actual signature. Bootstrap p-values are computed by using these empirical null distributions.

Supplementary Figure 24

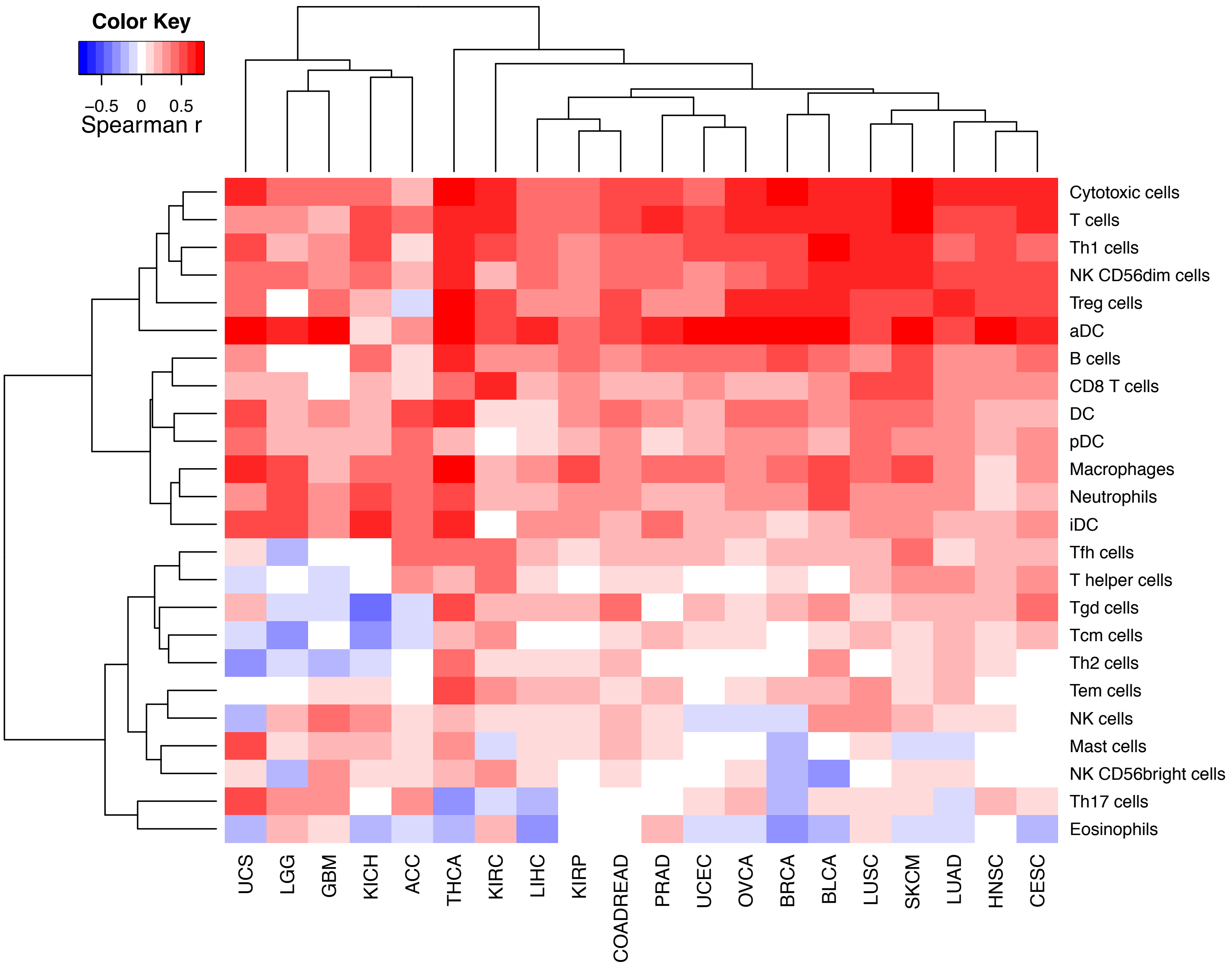

Figure S24. The correlation between the APM signature score and each infiltrating immune cell in TCGA samples. In cancer types with low TIS-APM correlations (GBM, LGG, KICH, ACC), APM is most strongly correlated with macrophages or dendritic cell subpopulations (DC, iDC, aDC).

Supplementary Figure 25

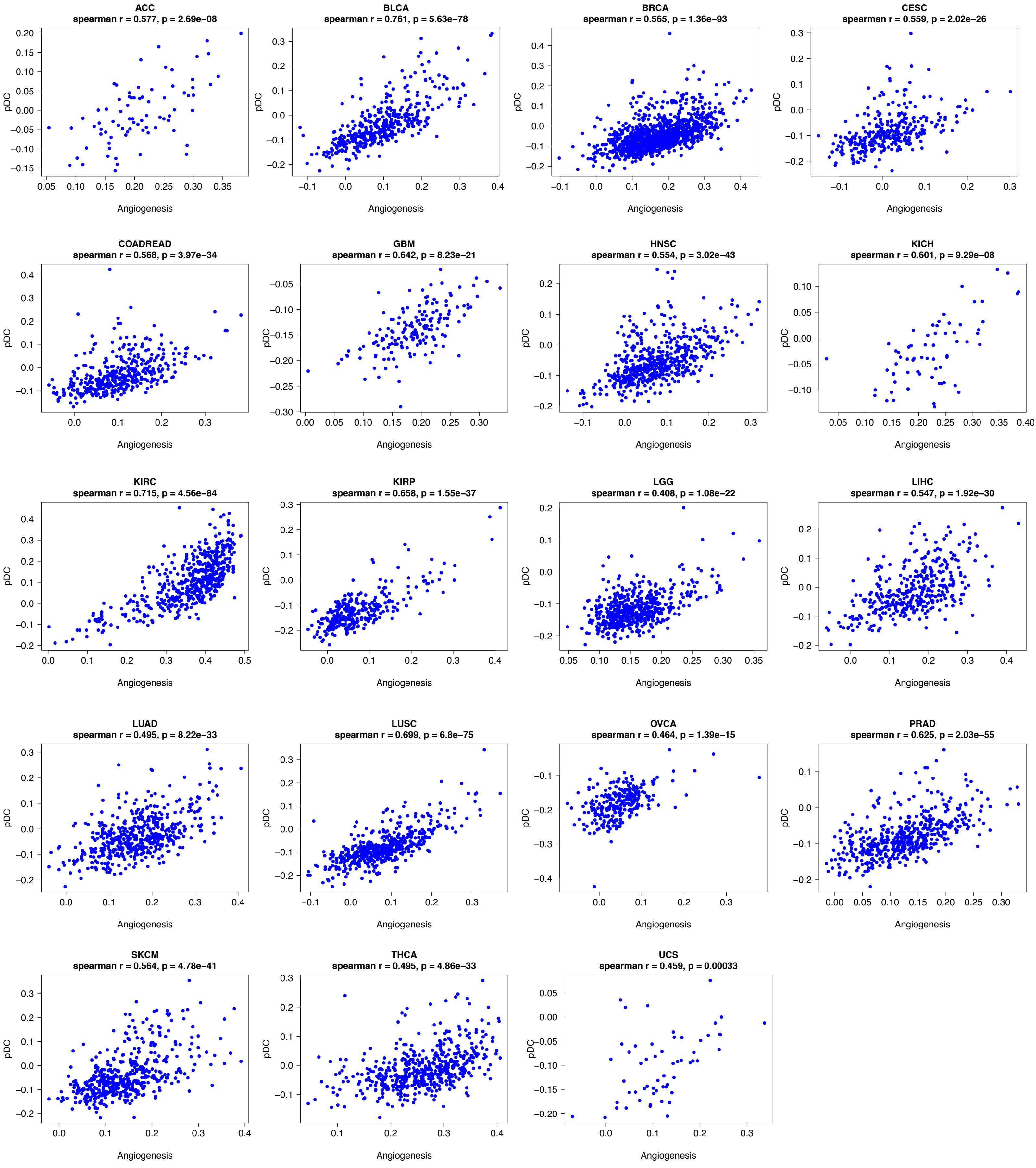

Figure S25. Correlation of angiogenesis and pDC scores across 19 cancer types.
The 1-gene pDC signature score is highly correlated with the 40-gene angiogenesis signature score across different cancer types.
